# Atomistic structures and dynamics of prenucleation clusters in MOF-2 and MOF-5 syntheses

**DOI:** 10.1038/s41467-019-11564-4

**Published:** 2019-08-23

**Authors:** Junfei Xing, Luca Schweighauser, Satoshi Okada, Koji Harano, Eiichi Nakamura

**Affiliations:** Department of Chemistry, The University of Tokyo, 7-3-1 Hongo, Bunkyo-ku, Tokyo 113-0033 Japan

**Keywords:** Transmission electron microscopy, Metal-organic frameworks, Coordination chemistry

## Abstract

Chemical reactions in solution almost always take place via a series of minute intermediates that are often in rapid equilibrium with each other, and hence hardly characterizable at the level of atomistic molecular structures. We found that single-molecule atomic-resolution real-time electron microscopic (SMART-EM) video imaging provides a unique methodology for capturing and analyzing the minute reaction intermediates, as illustrated here for single prenucleation clusters (PNCs) in the reaction mixture of metal–organic frameworks (MOFs). Specifically, we found two different types of PNCs are involved in the formation of MOF-2 and MOF-5 from a mixture of zinc nitrate and benzene dicarboxylates at 95 °C and 120 °C, respectively. SMART-EM identified a small amount of 1-nm-sized cube and cube-like PNCs in the MOF-5 synthesis, but not in the MOF-2 synthesis. In the latter, we instead found only linear and square PNCs, suggesting that the MOF-2/-5 bifurcation takes place at the PNC stage.

## Abstract (web summary)

Numerous techniques have been used to study the crystallization process of metal-organic frameworks, but little is known about their prenucleation clusters. Here the authors use single-molecule atomic-resolution real-time electron microscopic video imaging to identify prenucleation clusters in the synthesis of MOF-2 and MOF-5.

## Introduction

**Fig. 1:**
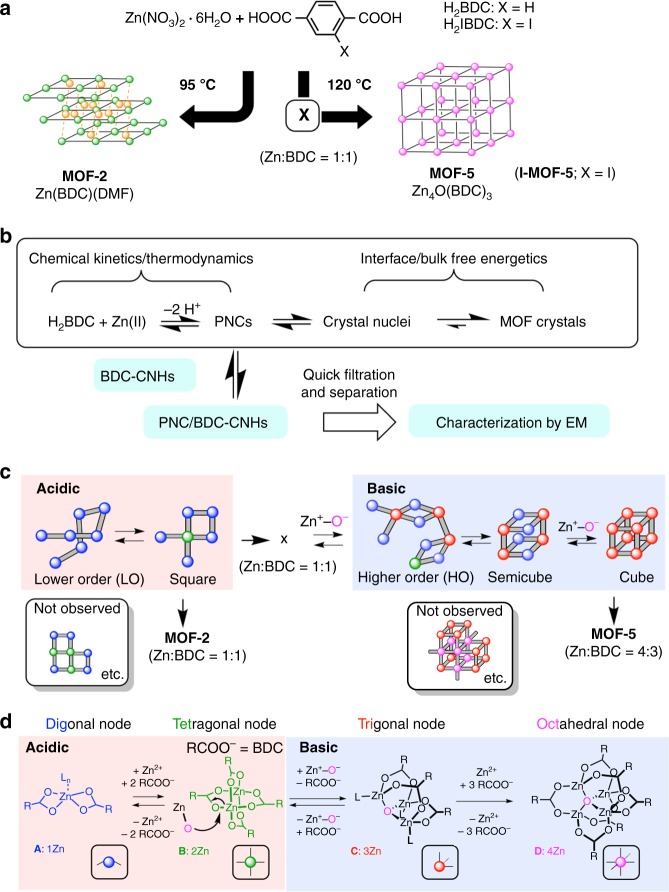
Formation mechanism of MOF-2 and MOF-5 crystals from Zn^2+^ and BDC. **a** Temperature-dependent bifurcation to MOF-2 and MOF-5 formed from zinc nitrate hexahydrate and H_2_BDC. H_2_IBDC yields isostructural I-MOF-5. See Supplementary Fig. 18 for crystal structure. **b** Overall pathway of MOF formation that consists of two stages, and capturing of PNCs on BDC-CNHs. **c** Schematic illustration of the reaction paths from LO PNCs to cube PNCs, and to MOF-2 and -5 crystals. **d** Structures of nodes consisting of Zn^2+^ and BDC

Metal-organic frameworks (MOFs) are porous minerals that consist of nodes comprising metal ions connected by organic linkers^1,2^. Their diversity in lattice structure, elemental composition and organic linkers offers tremendous opportunities in materials applications^3,4^. Easy access to diverse structures is an asset of MOF science and technology, but their synthesis often shows hints of mechanistic complexity. As a classic example, MOF-2 having square lattice and MOF-5 having cubic lattice (Fig. 1a)^5^ differ only in the conditions of the reaction between zinc nitrate and benzene dicarboxylic acid (H_2_BDC) in dimethylformamide (DMF): heating at 95 °C for several hours produces MOF-2 (Zn:BDC = 1:1, isolated in its DMF solvated form)^6^, and the mixture becomes acidic (Zn(NO_3_)_2_ + 2•RCOOH = Zn(RCOO)_2_ + 2•HNO_3_). Heating at 120 °C produces between 0 and 4 h non-porous precipitates of unknown structure (Zn:BDC = 1: 1, called herein as **X**)^7^, which gradually changes in situ to MOF-5 nanocrystallites as the solution changes from acidic to basic^8^ because of thermal decomposition of DMF that generates a formal water dianion (O^2−^ or Zn^+^−O^−^)^9^. For a few tens of hours after formation, the nanocrystallites undergo Ostwald ripening to produce MOF-5 cubic polycrystals (Zn:BDC = 4:3)^10^. Thus, the system conforms the kinetics and thermodynamics of the reaction intermediates that serve as prenucleation clusters (PNCs) of crystallisation controlled by interface and bulk free energetics (Fig. 1b)^11^. Although the MOF-2/-5 bifurcation suggests structural difference among the PNCs leading either MOF-2 or MOF-5 (Fig. 1c)^12,13^, little has been known for PNCs in solution at molecular level^14^. Earlier in situ studies by static light scattering^15^, extended X-ray absorption fine structure^16^, and liquid cell transmission electron microscopy (TEM)^17^ revealed small crystals but not PNCs^12,13^. PNCs were identified only by mass spectrometry^18^ and by computer simulation^19^. In this context, we focused on the MOF-2 and -5 formation^20^ through in situ capturing of PNCs and atomistic structural analysis by single-molecule atomic-resolution real-time electron microscopy (SMART-EM)^21,22^.

In Fig. 1d, we summarise the correlation between the node structures based on reported crystal structures. The mononuclear structure of a digonal node A is ubiquitous among zinc carboxylates^23^ and forms linear polymers of Zn-BDC as found commonly in the MOF-2 and -5 synthesis. The dinuclear complex B is simply a dimer of A responsible for the formation of square PNCs, and represents a tetragonal node in MOF-2. The structural relationship between the mononuclear node A and a tetranuclear node D parallels the one between zinc acetate and basic zinc acetate (Zn_4_O(CH_3_COO)_6_), the latter converted readily to the former upon acidification^20^. The trinuclear node C is an intermediate to D formed by replacement of one RCOO^–^ group on B by Zn^+^−O^−^^24^. Thus, the formation of D (MOF-5 node) from C requires the addition of one zinc cation and three molecules of BDC, with a considerable entropy loss^25,26^. This chemical diagram suggests that the linear and square (lower order, LO) PNCs made only of Zn and BDC should form readily under mild conditions, while the cube and cube-like (higher order, HO) clusters requiring nodes C and D should increase in number as DMF decompose upon prolonged heating at 120 °C. We verified this hypothesis experimentally by SMART-EM studies of the clusters isolated from the reaction mixture as described below.

Here, we report that the MOF-2 synthesis produces square-shaped clusters, while the MOF-5 synthesis produces cube and cube-like clusters as structurally the most complex PNCs (Fig. 1c). Commonly found in both cases were linear clusters of considerable structural flexibility - zinc carboxylate oligomers (Fig. 1c, d (A)). In the synthesis of iodinated MOF-5 (I-MOF-5) from 2-iodoterephthalic acid (H_2_IBDC), we established the structure of a slowly rotating 1.3-nm-sized cube cluster by determining the spatial locations of all 12 iodine atoms in sequential 2-D video images with approximately 1 Å precision. The SMART-EM technique recently revealed the feasibility of single-molecule level kinetics^27–30^, and now allows us to investigate atomistic structures of minute intermediates of chemical reactions.

## Results

### Capturing PNCs of MOF-2 and MOF-5 on BDC-CNH

**Fig. 2:**
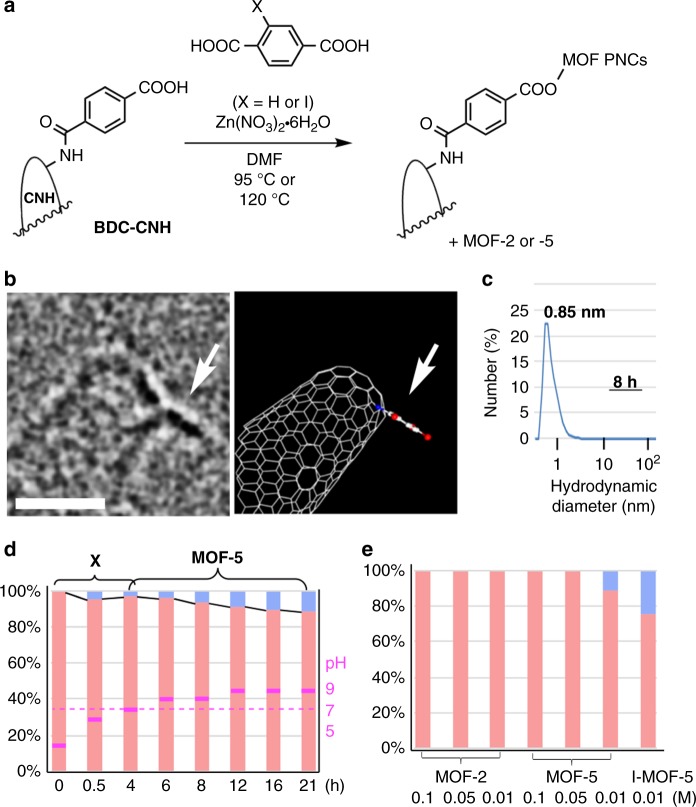
BDC-CNH fishhook for capturing PNCs of MOF-2 and MOF-5. **a** Procedure to capture PNCs of MOF-2 and MOF-5 on a ‘fishhook’ of BDC-CNH. **b** TEM image (left) and molecular model (right) of a BDC moiety (indicated by an arrow) bound to the tip of the CNH. The scale bar is 1 nm. See Supplementary Fig. 7 for details. **c** DLS analysis (particle number average) of the filtrate of a reaction mixture of MOF-5 after 8 h. **d** The ratio between LO (pink) and HO (blue) clusters in the formation of **X** and MOF-5 at 120 °C. The data in purple indicate the pH of reaction mixture after dilution with water. The total number of the PNCs examined in the eight experiments shown are the following from the left to the right: 47, 27, 132, 133, 67, 58, 69 and 36. See in Supplementary Table 2 for the original data. **e** The LO/HO ratio at various Zn^2+^ concentrations, as captured on BDC-CNH from the reaction of a mixture of H_2_BDC (or H_2_IBDC), Zn(NO_3_)_2_•6H_2_O (2 equiv), and BDC-CNH heated for 21 h at 95 °C (MOF-2 formation) and 120 °C (MOF-5 and I-MOF-5). The total number of the PNCs examined in the seven experiments shown are the following from the left to the right: 45, 48, 56, 73, 33, 36 and 54. See in Supplementary Table 3 for the original data

For this study, we designed a ‘fishhook’ for the in situ ‘fishing’ of PNCs, and connected BDC molecules as such a fishhook via an amide linkage onto carbon nanohorn aggregates (BDC-CNH; Fig. 2a, b). Being a part structure of the covalent network of MOF crystals, the BDC group on CNH serves as a chemically powerful “fishhook” for capturing PNCs in solution so that we can study their structure one by one by EM. Thus, we heated a mixture of H_2_BDC (or H_2_IBDC), Zn(NO_3_)_2_•6H_2_O (2 equiv), and BDC-CNH (1 × 10^−2^−10^−3^ molar equiv –NH_2_ units/BDC) in dry DMF at 95 °C and 120 °C for MOF-2 and -5 synthesis over a period between 0 h and 21 h. To stop the MOF forming reactions taking place at high temperature, we cooled down the reaction mixture quickly to 25 °C, immediately followed by removal by filtration of the solvent and the soluble materials to prevent further reactions. A mixture of white crystals and a BDC-CNH black powder was isolated on a 200-nm-pore filter, and directly transferred to a TEM sample grid (dry transfer) for observation by SMART-EM (Supplementary Fig. 14) and scanning electron microscopy (SEM, Supplementary Figs. 10 and 13). Powder X-ray diffraction (PXRD) analysis of the solid sample (Supplementary Figs. 8, 9, 11, and 12), and dynamic light scattering (DLS) analysis of the filtrate were also carried out.

The MOF-5 synthesis first produced **X** after 0.5–4 h as white precipitates, which was converted in situ to MOF-5 nanocrystallites at 120 °C for a few hours, and then to cubic MOF-5 polycrystals after 21 h. Throughout the 21 h reaction time, DLS analysis of solution showed only one peak at 0.78–0.98 nm hydrodynamic diameter (Fig. 2c for 8 h, MOF-5; further data in Supplementary Fig. 15 and 16). We found no evidence of PNCs in solution >1 nm and smaller than sub μm crystals, such as those shown in black boxes in Fig. 1c. In agreement with the DLS data, the clusters captured on BDC-CNHs and identified by SMART-EM had ~1 nm in size.

SMART-EM study of LO and HO clusters captured on the BDC-CNHs from the MOF-5 synthesis (120 °C, 0.01 M zinc concentration) showed that the ratio changes along the reaction time (Fig. 2d). Thus, the amount of the HO clusters relative to the LO ones increased steadily throughout the reaction as DMF continued to decompose, in particular, after 6 h when all **X** was converted to MOF-5 and the solution changed from acidic to basic (Fig. 2d). Figure 2e shows the LO/HO ratio after 21 h at zinc concentration between 0.01 and 0.1 M. We see that the MOF-2 synthesis (95 °C) produced only the LO clusters, whereas the MOF-5 and I-MOF-5 synthesis (120 °C) produced a substantial amount of HO clusters at 0.01 M conditions (Supplementary Fig. 17). The observed difference between MOF-2 and -5 is statistically significant (*p* = 0.011 in the two-sample *t*-test). With 27–133 samples examined in each analysis, we expect 8–19% statistical error for the reported HO/LO ratio.^31^

**Fig. 3:**
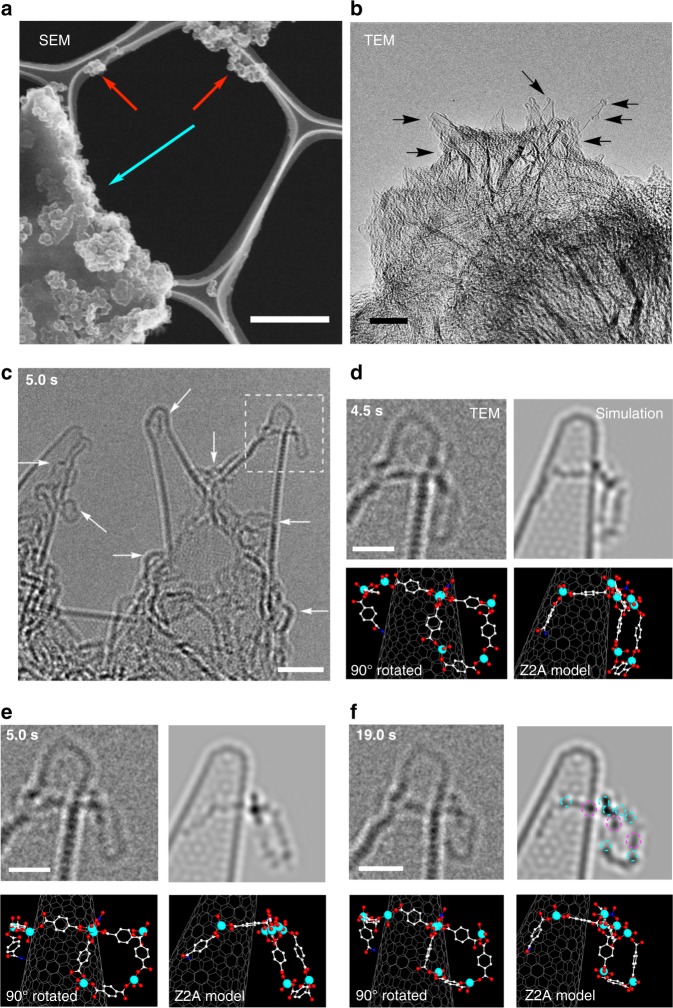
Electron microscopic imaging of PNCs in MOF-2 and MOF-5 syntheses at 0.01 M of Zn^2+^ for 21 h. **a** SEM image of a MOF-5 crystal (blue arrow) and agglomerates of BDC-CNH particles (red arrows) in a sample dry transferred to a TEM microgrid. The scale bar is 1 μm. **b** TEM image of a BDC-CNH particle trapping PNCs (arrows) of I-MOF-5 (dry transfer). The scale bar is 10 nm. **c** Numerous 1-D and 2-D PNCs (arrows) of MOF-2 on BDC-CNH (methanol wash). The scale bar is 2 nm. **d–f** A TEM video frame, TEM simulation, and Z2A model of the 2-D PNC of the boxed area in **c** taken at various times after starting the video recording (the same for time stamps of all video frames below): **d** 4.5 s, **e** 5.0 s, and **f** 19.0 s. Colour coding: O, red; Zn, light blue; N, blue; C, grey (the same colour codes for all models below). See Supplementary Fig. 3 for details. The original video is in Supplementary Movie 1. The scale bar is 1 nm. Positions of zinc atoms (light blue) and benzene rings parallel to the electron beam (purple) are circled in **f**

The SEM image of the dry transfer sample of MOF-5 synthesis in Fig. 3a shows a several μm sized rectangular MOF-5 crystal covered by CNH aggregates (light blue arrow), and agglomerates of CNH aggregates on the grid (red arrows). No intermediary-sized objects were detected. Similarly, in the low magnification TEM image of the CNH surfaces in Fig. 3b, we observed only nanometre-sized molecular PNCs, and nothing larger growing from the CNH surfaces (further data given below). Details of TEM analysis of MOF-5 clusters will be described later.

### Imaging of PNCs in MOF-2 synthesis

In the MOF-2 synthesis in the presence of BDC-CNH at 95 °C, we found numerous LO clusters (Fig. 3c and Supplementary Fig. 19), and nothing more complex (cf. Fig. 2e). The CNH particles and the clusters in dry transfer samples are often very mobile at an angstrom level (vide infra). Brief washing of the CNH specimens with methanol (for ca. 20 s) suppressed such motions of the CNHs and the clusters, and allowed us to reproducibly obtain atomic-resolution TEM images without apparent change in the appearance of the clusters. Methanol being an inert solvent for MOF-5,^32^ the methanol wash did not wash away the clusters made of a covalent Zn–O network, while it may have replaced the weakly coordinated ligands on zinc (such as DMF and nitrate anions, L in Fig. 1d). Figure 3d–f shows TEM video images of a swinging square (box in Fig. 3c) taken at intervals of 4.5, 5.0, and 19.0 s after the start of the observation (two frames per second, throughout; all frames shown in Supplementary Fig. 26), together with their simulations and molecular models.

**Fig. 4:**
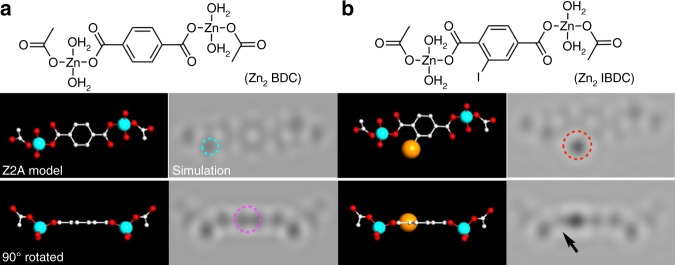
Molecular models and TEM simulation images of Zn-BDC complexes. **a** dinuclear Zn-BDC. **b** dinuclear Zn-IBDC. Positions of zinc atoms (light blue), benzene rings parallel to the electron beam (purple), and iodine atoms (red) are circled in **a** and **b**

For structural analysis and presentation below, we used a ball-and-stick model using atomic radii ∝ *Z*^2/3^ (atomic number *Z*; hence denoted as *Z*^2^ adjusted model or Z2A model), because the intensity of an atomic image is approximately proportional to *Z*^2^^33^. In this model, a heavier atom is seen as a larger sphere, providing a visual impression close to the corresponding TEM image. TEM simulations for dinuclear Zn complexes of BDC and IBDC are shown in Fig. 4a, b. A zinc atom (blue circle; *Z* *=* 30), a benzene ring parallel to the electron beam (purple), and particularly, an iodine atom (red) produce characteristic dark and bright Fresnel fringes, which enhance or attenuate the contrast of neighbouring atoms (black arrow in Fig. 4b). Details of TEM image simulation are described in the Supplementary Methods and Supplementary Figs. 1 and 2.

### Imaging of PNCs in MOF-5 synthesis

**Fig. 5:**
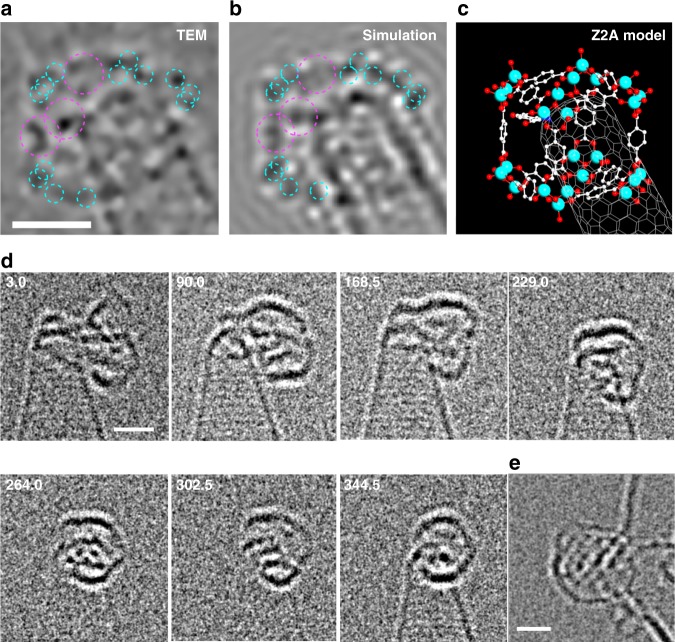
TEM imaging of MOF-5 and I-MOF-5 PNCs at 0.01 M of Zn^2+^ for 21 h. **a** TEM image of a cubic PNC of MOF-5 (methanol wash), **b** a simulated image, and **c** a Z2A model corresponding to **b**. Zinc atoms and benzene rings are circled in the same manner as in Fig. 4. The original video is Supplementary Movie 2. The scale bar is 1 nm. **d** Seven frames from a 345-s video showing the structural reorganisation of a HO I-MOF-5 PNC (dry transfer). The original video is Supplementary Movie 3. The scale bar is 1 nm. **e** A hexagonal-shaped PNC recovered from I-MOF-5 synthesis found in a dry transferred sample

Although the clusters captured on BDC-CNH in the synthesis of MOF-5 were largely LO clusters (Supplementary Figs. 20 and 25), there also formed a small number of cube-shaped clusters (Fig. 2e and Supplementary Fig. 21). Video image of one of the cubes was taken for a period of 37.2 s (Supplementary Fig. 27; image of another cube captured at 16 h is shown in Supplementary Fig. 24). Careful analysis of a frame at 22.8 s (Fig. 5a) shows that the image best fits in a cube structure, seen roughly from an isometric direction. Figure 5a–c shows the correlation of the experimental image to a Z2A model via its simulation. The structural stability of this cluster over 37.2 s, compared with a structurally mobile cluster (Fig. 5d) agree with the cube structure. The cubic cluster lacks any discernibly large contrast at the corners of the hexagon, suggesting that the corner consists of a trigonal node **C** (Fig. 1d) instead of an octahedral node **D** that ought to have extra BDC groups (R) on every corner. A number of dots in the hexagon (except the area overlapping with the CNH) can be rationalised in terms of zinc atoms (blue) and benzene rings parallel to the electron beam (purple), as compared with a simulation (Fig. 5b) based on a molecular model (Fig. 5c). Figure 5e shows another complete cube, found in the I-MOF-5 synthesis^34^. As summarised by a histogram in Fig. 2e, cube and cube-like PNCs formed only at 0.01 M zinc nitrate concentration, suggesting that they are less abundant in solution because they quickly grow into MOF-5 crystals at higher concentrations. This concentration dependence is consistent with the large entropic penalty of the conversion of the trigonal node C to the octahedral node D.

Similar to the MOF-2 mixture, the MOF-5 mixture also contained numerous LO clusters (Fig. 2e), while they appeared to be structurally more complex than those in the MOF-2 synthesis. Particularly noteworthy was spontaneous conversion to what appeared to be cube-like structures as observed for three cases out of several tens of clusters observed clearly. In one example shown in Fig. 5d, a few large rings identifiable in the 3.0-s image started to move after 25 s, forming a stable 3-D structure after 264.0 s (Supplementary Fig. 28). The video suggests that a similar process may take place in solution. This conversion obviously requires a large entropic penalty and hence suggests enthalpic advantage. We found two additional examples of such conversion for samples after methanol wash (Supplementary Figs. 29 and 30; see also Supplementary Movies 4 and 5). LO clusters accounted for approximately 80% of all PNCs in the I-MOF-5 synthesis (Fig. 2e, Supplementary Figs. 22 and 23).

### Structural identification of PNCs in I-MOF-5 synthesis

**Fig. 6:**
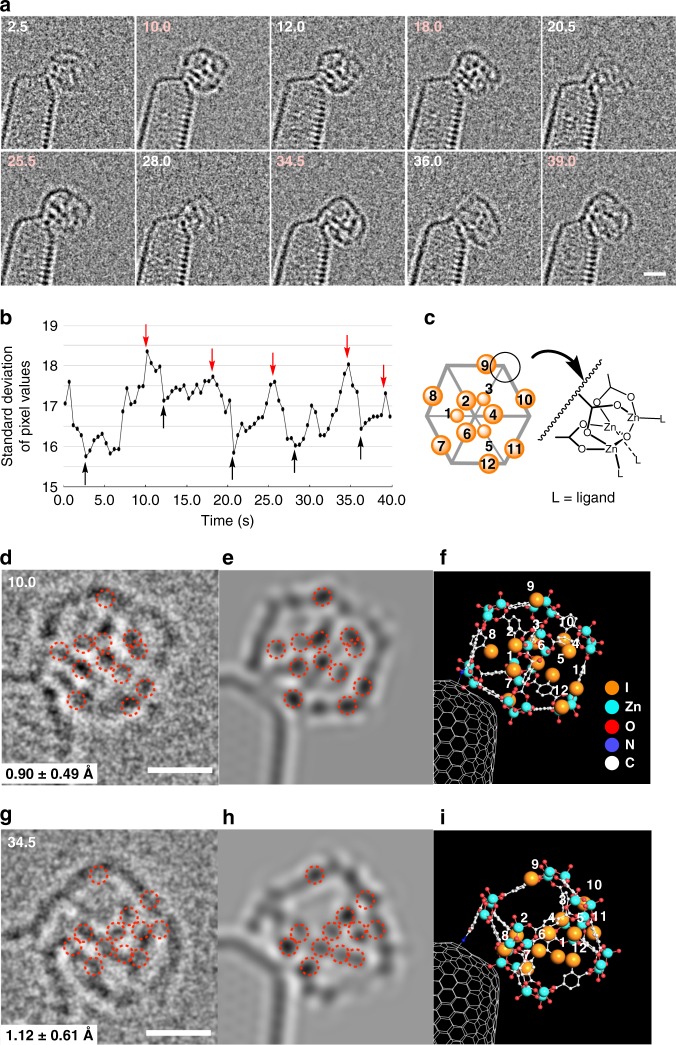
TEM imaging and structure assignment of PNCs in I-MOF-5 synthesis at 0.01 M of Zn^2+^ for 21 h. **a** Ten SMART-EM images out of 80 video frames of a cubic PNC made of IBDC rotating in a stochastic manner (methanol wash). The original video is Supplementary Movie 6. **b** Time-dependent change of the standard deviation of the pixel values in the video frames. Five high contrast frames, shown in **a**, are indicated by red arrows and five low contrast frames by black arrows. **c** A model structure of the cubic PNC at 10.0 s, where the 12 iodine atoms are numbered and their positions in 2-D projections are highlighted. **d** An isometric projection of the cube at 10.0 s, where 12 spots of iodine atoms are highlighted by red circles. **e** A simulated image and **f** a Z2A model corresponding to **e**. **g** A dimeric projection at 34.0 s, **h** simulated image, and **i** Z2A model corresponding to **h**. Figures **c**, **f** and **i** share the same numbers for assignment of the 12 iodine atoms

Labelling of MOF-5 with iodine (*Z* = 53) facilitated the structure assignment of MOF-5. Figure 6 shows a series of images of a PNC, ~1.3 nm in size, formed during I-MOF-5 synthesis. During an observation period of 40 s over 80 frames (all frames shown in Supplementary Fig. 31), the cluster rotated slowly and stochastically, and the rotation of the whole PNC is illustrated across 10 frames in Fig. 6a. We found that the rotational motion of the whole cluster can be quantified by the standard deviation of the pixel values (Fig. 6b)^35^. Thus, larger values indicated higher contrast (red arrows, less motion), and smaller deviation values indicated lower contrast (i.e., rapid motion during the 0.5-s exposure time). In agreement with this quantified data in Fig. 6b, the molecule largely stood still at 10.0 s, 18.0 s, 25.5 s, 34.5 s, and 39.0 s (Fig. 6a, pink), allowing us to view the cube from five different directions, and hence enabling us to determine the locations of the iodine atoms in the 3-D space. During the rotation of the whole molecule, a few of the IBDC linkers also underwent a small rotational motion during the 0.5-s observation time to cause blurring of some iodine images, allowing us to assign a cube structure having 12 iodine atoms to the cluster, and the regiochemistry of the iodine atoms on the BDC linker, but not the structural details of the rotational isomerism (Supplementary Fig. 5)^34,36.^ Fig. 6c illustrates our regiochemical assignment of the IBDC linkers together with the trigonal node model that we used (L = H_2_O and OH^−^; the precise identification of L is impracticable because of bond rotations).

Structure assignment using the 10.0-s image (Fig. 6d) is illustrative. We started from a crystal lattice of MOF-5^37^ and replaced 12 hydrogen atoms with 12 iodine atoms. To begin the analysis, we focused on the six or seven dark iodine spots at the centre of the image at 10.0 s and 34.5 s, which then limited the number of regioisomers from 4096 to 14 (including the one in Fig. 6c and various enantiomers in Supplementary Fig. 4. See Supplementary Methods)^38^. We rotated all IBDC linkers to create numerous simulations and identified one (Fig. 6e, h) to be as close as the experimental images (Fig. 6d, g, and three others in Supplementary Fig. 6). We identified the iodine atoms in the simulation and the experimental images by using the iodine positions in the molecular model (Fig. 6f, i) as reference (red circles), and measured the difference between the experimental and simulation images to be 0.90 ± 0.49 Å, 1.15 ± 0.75 Å, 1.23 ± 0.74 Å, 1.12 ± 0.61 Å and 1.15 ± 0.41 Å for 10.0 s, 18.0 s, 25.5 s, 34.5 and 39.0 s, respectively (see Supplementary Fig. 5 for details). In light of the spatial resolution of our TEM (1 Å), we considered that the iodine locations determined in five different 2-D projections serve as a reliable measure for assigning the spatial location of the iodine atom and, hence, for assigning a cube structure to the observed PNC.

## Discussion

Through the combined use of DLS analysis in solutions and microscopic analysis of the solvent-free products, we found that only two kinds of molecular assemblies formed in the zinc MOF syntheses: MOF crystals and 1-nm-sized PNCs. From the data we obtained for the size distribution, the mobility, the intracluster conversion and the atomistic structures of PNCs, we consider that the MOF-2/-5 synthesis involves a series of equilibria among the starting materials, PNCs, and MOF crystals, and the square and the cubic feature of the MOF-2 and -5 lattices already appear among the reaction intermediates found in solution. The data set provided reasons for us to consider that the covalent skeleton of the clusters captured on the CNH fishhooks represents a good approximation of their structure in solution, while they likely differ in details as to the rotational isomerism of the BDC rotors, and the ligands weakly coordinated on the zinc atoms; namely, we expect a priori that the clusters on BDC-CNH seen by EM have stoichiometry and covalent structure closely related to those in the reaction mixture. Such a dataset is uniquely available by the use of the ‘fishing’ strategy described, combined with the TEM video imaging, and highlight the potential of the SMART-EM technology in the study of the mechanisms of chemical reactions in solution.

## Methods

### General

Unless otherwise noted, all of the reactions were conducted in a dry reaction flask made of glass under argon or nitrogen at atmospheric pressure. The water content of the solvent was confirmed with a Karl-Fischer Moisture Titrator (MKC-210, Kyoto Electronics Company) to be <100 ppm. Distilled water was further purified with Millipore Milli-Q. Bath sonication for dispersion of CNHs was carried out on a Honda Electronics WT-200-M instrument. Powder X-ray diffraction (PXRD) measurements were carried out on Rigaku SmartLab. Dynamic laser light scattering (DLS) was carried out on a Malvern Zetasizer Nano ZS machine. pH measurement was carried out by using pH paper (UNIV, Toyo Roshi Co. Ltd) or HORIBA pH metre D-51 with an electrode 9680 S. FE-SEM observation was carried out on a FEI Magellan 400 L operating with acceleration voltage of 1 kV and beam current of 1.3–25 pA. The size analysis of TEM and SEM images was conducted by ImageJ 1.47t software.

### Materials

Unless otherwise noted, materials were purchased from Tokyo Kasei Co., Aldrich Inc. and other commercial suppliers, and used after appropriate purification. All other solvents were purified by distillation and stored over molecular sieves 4 Å. TEM grids precoated with a lacy carbon (NS-C15, pore size 1.5–8 μm) were purchased from Okenshoji Co., Ltd. Carbon nanohorn was purchased from NEC Corporation and used as received. Amino carbon nanohorn^39^, 2-iodoterephthalic acid^40^, and 1,4-disuccinimidyl-terephthalic acid^41^ was prepared according to previous reports.

### Preparation of BDC-CNH

1,4-Disuccinimidyl-terephthalic acid (9.6 mg, 26 µmol, 10 eq vs. -NH_2_) was mixed with a powder of amino carbon nanohorn (264 nmol/mg of -NH_2_ groups, 10 mg) in 2 mL of DMF/H_2_O (1:1, v/v) in a 10-mL flask. After stirring at room temperature for 24 h, LiOH•H_2_O (1.1 mg, 10 eq vs. -NH_2_ groups) was added to the mixture and stirred for 24 h. 1 M HCl (2.0 mL) was then added to the mixture and stirred for 5 min. The resulting suspension was filtered through a PTFE membrane filter (ADVANTEC, pore size: 100 nm) and washed with H_2_O (1 mL × 3) and DMF (1 mL × 3), to obtain 9.2 mg of BDC-CNH as a black powder after filtration through and vacuum drying (60 Pa) for 12 h. The attachment of the BDC moiety to CNH was ascertained by TEM imaging (Supplementary Fig. 7).

### Formation of MOF-2 in the presence of BDC-CNH at c(Zn^2+^)=0.01M

Zn(NO_3_)_2_•6H_2_O (15 mg, 0.050 mmol) was mixed with terephthalic acid (BDC, 4.2 mg, 0.025 mmol) and BDC-CNH (264.0 nmol/mg of -NH_2_ groups, 0.95 mg, 5 × 10^−3^ eq vs Zn^2+^) and stirred in DMF (5.0 mL) in a 10-mL flask for 21 h at 95 °C. After quickly cooling with a water bath at room temperature, the reaction mixture was filtered through a PTFE membrane filter (ADVANTEC, pore size: 100 nm) and washed with DMF (1 mL × 3) to obtain 6.0 mg of a black powder (a mixture of MOF-2 crystals and BDC-CNH) after vacuum drying (60 Pa) for 12 h. The solid sample was analysed by TEM and PXRD. Formation of MOF-2 was confirmed by PXRD measurement (Supplementary Fig. 8). The experiment without BDC-CNH was conducted in same condition. The solution was filtered and analysed by DLS. The yield is shown in Supplementary Table 1.

### Preparation of MOF-5 in the presence of BDC-CNH at c(Zn^2+^)=0.01M

Zn(NO_3_)_2_•6H_2_O (15 mg, 0.050 mmol) was mixed with terephthalic acid (4.2 mg, 0.025 mmol) and BDC-CNH (264.0 nmol/mg of -NH_2_ groups, 0.95 mg, 5 × 10^−3^ eq vs Zn^2+^) and stirred in DMF (5.0 mL) in a 10 mL flask for 21 h at 120 °C. After quickly cooling with a water bath at room temperature, the reaction mixture was filtered through a PTFE membrane filter (ADVANTEC, pore size: 100 nm) and washed with DMF (1 mL × 3) to obtain 9.5 mg of a black powder (a mixture of MOF-5 crystals and BDC-CNH) after vacuum drying (60 Pa) for 12 h. The solid sample was analysed by SEM, TEM and PXRD. Formation of MOF-5 crystal was confirmed by PXRD measurement (Supplementary Fig. 9). The experiment without BDC-CNH was conducted under the same condition. The solution was filtered and analysed by DLS. The yield is shown in Supplementary Table 1.

### Preparation of samples for EM

Dry transfer: The overall procedure from the MOF synthesis to TEM the imaging is shown in Supplementary Fig. 14. After MOF synthesis in the presence of BDC-CNH, a small portion of the mixture of MOF crystals and BDC-CNH was transferred manually to a SEM substrate or TEM grid to obtains images such as Fig. 3a, b. These low magnification images attested to the entire absence of objects of sizes between nm-sized PNC and MOF crystals, which agrees with the DLS analysis of the MOF synthesis solution. Methanol wash: The CNH particles and PNCs obtained by dry transfer samples are often mobile at an Å level (cf. Figure 5d), and atomic-resolution TEM images were obtained reproducibly after suspending the mixture in methanol. The methanol wash did not change the appearance of PNCs as analysed at the present TEM resolution. The MOF/BDC-CNH mixture was dispersed in methanol (1 mg/mL) by bath sonication (2 seconds × 5). After sonication, a crystalline powder of MOF precipitated and a part of BDC-CNH particles was suspended in an upper layer. Then, 5 μL of the suspension was collected with a pipette and drop casted onto a TEM microgrid. The TEM grid was dried in vacuum (60 Pa) to remove solvent for 12 h.

### TEM observation

Atomic-resolution TEM observation was carried out on a JEOL JEM-ARM200F instrument equipped with an aberration corrector (point resolution: 0.10 nm) at 298 K and at acceleration voltages of *E* = 80 or 120 kV, under 1 × 10^−5^ Pa in the specimen column. We adjusted spherical aberration (*C*_*s*_) values to 1–3 μm and an electron dose rate (EDR; the number of electrons per second per nm^2^) of 1.1−15.3 × 10^5^ e^–^ nm^−2^ s^−1^ at × 800,000–2,000,000 magnification. For 80 kV observation, a series of TEM images were continuously recorded every 0.5 s on a CMOS camera (Gatan OneView, 4096 × 4096 pixels) and operated in binning 2 mode (output image size: 2048 × 2048 pixels, pixel resolution 0.01 nm at × 2,000,000). For 120 kV observation, a series of TEM images was obtained at intervals of 1.2 s with an exposure time of 0.4 s followed by a readout time of 0.8 s (non-irradiated) on a charge-coupled device camera (Gatan multiscan). All the images were automatically processed on Gatan DigitalMicrograph software. To record the atomic-resolution videos of PNCs, we first surveyed the whole CNHs agglomerates on the grid at × 400,000 magnification to find CNHs for PNCs observation. Then, we changed magnification to ×800,000–2,000,000 and started observation and video recording. Focusing was carried out during the collection of images, which was recorded at overfocus or underfocus conditions (defocus value: 10–20 nm). Most of the PNCs changed their shape during observation. The images were collected as a .dm3 format file on Gatan DigitalMicrograph software and processed using ImageJ 1.47t software. All images were filtered by a bandpass filter (filtering structures smaller than 3 pixels and larger than 40 pixels, tolerance of direction: 5%). If necessary and possible, the data sets were aligned to minimise specimen drift by correcting the cross-correlation of each image using StackReg and TurboReg plug-in software on ImageJ^42^. Images taken under an overfocus condition were contrast-inverted for consistency in presentations throughout this work. The images were adjusted with brightness and contrast. Finally, the orientation of CNHs in all TEM images, simulations and models were fixed into upright.

### Simulation of TEM images

TEM simulation images were generated for the structure by using a multi-slice procedure implemented in a Bionet elbis software^43^, where a defocus value of 10–20 nm and a spherical aberration coefficient (*Cs*) of 1–3 µm were used, and Gaussian-blurred to reproduce the effect of vibration.

## Supplementary information

Supplementary Information

Description of Additional Supplementary Files

Supplemmentary Movie 1

Supplemmentary Movie 2

Supplemmentary Movie 3

Supplemmentary Movie 4

Supplemmentary Movie 5

Supplemmentary Movie 6

Peer Review File

## Data Availability

The authors declare that all the other data supporting the findings of this study are available within the Article and its Supplementary Information files and from the corresponding author upon request.
