# Supplementary material for: Atomistic structures and dynamics of prenucleation clusters in MOF-2 and MOF-5 syntheses
Source: Nat Commun. 2019 Aug 23;10:3608. doi: 10.1038/s41467-019-11564-4 (PMC6707309; doi:10.1038/s41467-019-11564-4)
Supplement: Supplementary file 1 — Supplementary Information [file 41467_2019_11564_MOESM1_ESM.pdf]

Supplementary Information for

Atomistic structures and dynamics of prenucleation clusters in MOF-2 and MOF-5 syntheses

Xing et al.

This PDF file contains:

Supplementary Methods
Supplementary Figures: Supplementary Fig. 1–31
Supplementary Tables: Supplementary Table 1–3
Supplementary References

Supplementary Methods

1. Z^2-adjusted model (Z2A model) for analysis of TEM images

The CPK model (Supplementary Fig. 1 left) represents a van der Waals sphere of a molecule as shown for a solvated zinc BDC complex below. This model is unsuitable for analysis of TEM images, however, because the scattering cross section of an electron is proportionate to Z^2 (Z = atomic number)[1]. Instead we developed a Z^2-adjusted model (Z2A model) shown in Supplementary Fig. 1 middle, where the radii of atoms are set to be proportional to $Z^{2/3}$. The Z2A model provides a visual impression much closer to the TEM image (Supplementary Fig. 1 right) than the CPK model.

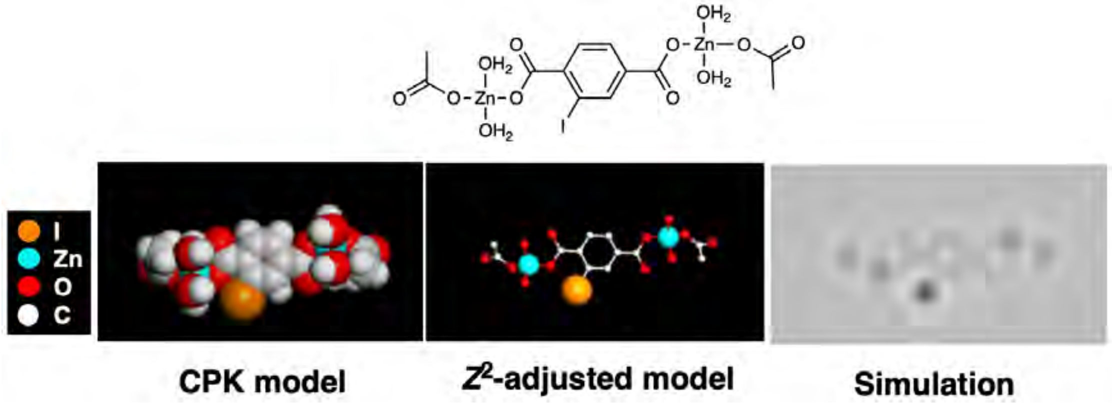

Supplementary Fig. 1. A CPK model, a Z^2-adjusted model and a corresponding TEM simulation image of dinuclear Zn^{2+} complex of IBDC.

The model construction from experimental images was achieved in a manner similar to the one by which chemists make a stereo model based on NMR. First, we guess at a plausible structure based on the experimental data, make a simulation based on the models, compare the experimental and the simulated data, and iterate the whole process until we find the best stereochemical model that accounts for important features of the experimental data. For images of static molecules (i.e., not blurred), the procedure produces a simulation close to the experimental image, for instance, within 1 Å difference of position of iodine atoms as show in the I-MOF-5 PNC shown in Fig. 6. The following atomic radii (in Å and nm) were used for the Z2A model:

H: Z = 1, not shown; C: Z = 6, 0.3 Å, 0.03 nm; N: Z = 7, 0.3 Å, 0.03 nm ; O: Z = 8, 0.4 Å, 0.04 nm; Zn: Z = 30, 0.9 Å, 0.09 nm; I: Z = 53, 1.3 Å, 0.13 nm

cf. CPK atomic radii

H: 1.20 Å, 0.120 nm; C: 1.70 Å, 0.170 nm; N: 1.55 Å, 0.155 nm ; O: 1.52 Å, 0.152 nm; Zn: 1.39 Å, 0.139 nm ; I: 1.98 Å, 0.198 nm

2. Structure assignment from TEM images

TEM images generated by electron interference contain a wealth of structural information. In Supplementary Fig. 2 (taken from Fig. 4a and b), we summarize a protocol of structural assignment. The image contrast reflecting square of an atomic number (Z), a zinc atom (Z = 30) creates a rather dark spot (blue circle in Fig. 4b) By the same token, a single iodine atom in IBDC (Fig. 4b red; Z = 53) produces a very dark contrast and a white fringe, which affect the contrast of neighboring images (enhance or attenuate; black arrow in Fig. 4b). A benzene ring that lies parallel to the

electron beam (purple; carbon, $Z = 6$) is seen as a dark spot. Upon 90° rotation, the benzene image fades away. The carbon spots are weaker and may be faded away by the presence of a nearby iodine atom (arrow). A carboxylate group may also give a rather dark spot when it lies parallel to the electron beam.

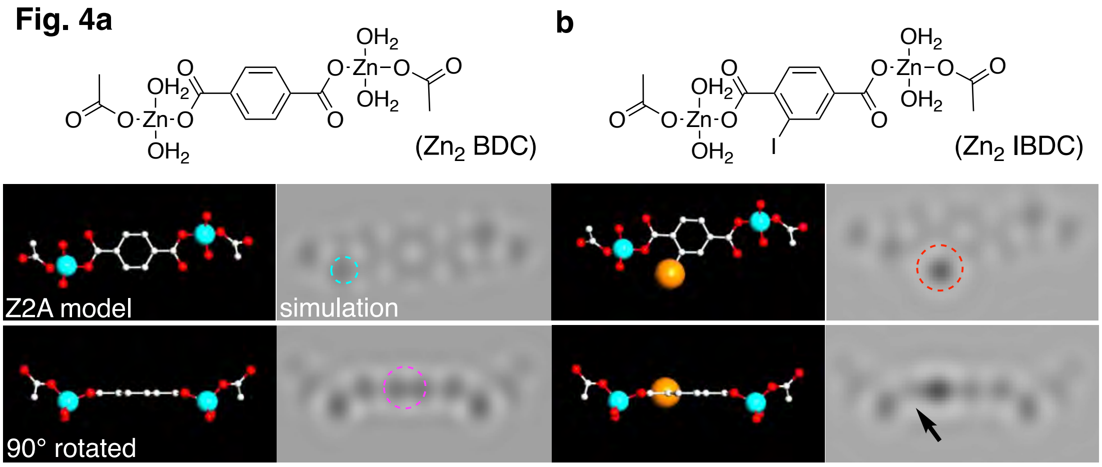

Supplementary Fig. 2. Molecular models and TEM simulation images of dinuclear Zn-BDC and Zn-IBDC complexes. Positions of zinc atoms (light blue), benzene rings parallel to the electron beam (purple) and an iodine atom (red) are circled.

Structure assignment of a PNC of MOF-2 on CNH (Fig. 3d–f).

According to the above protocol, we assigned a square structure to the swinging cyclic object shown in Fig. 3d–f. Four TEM images corresponding those in Fig. 3d–f are shown below together with simulation and Z2A models.

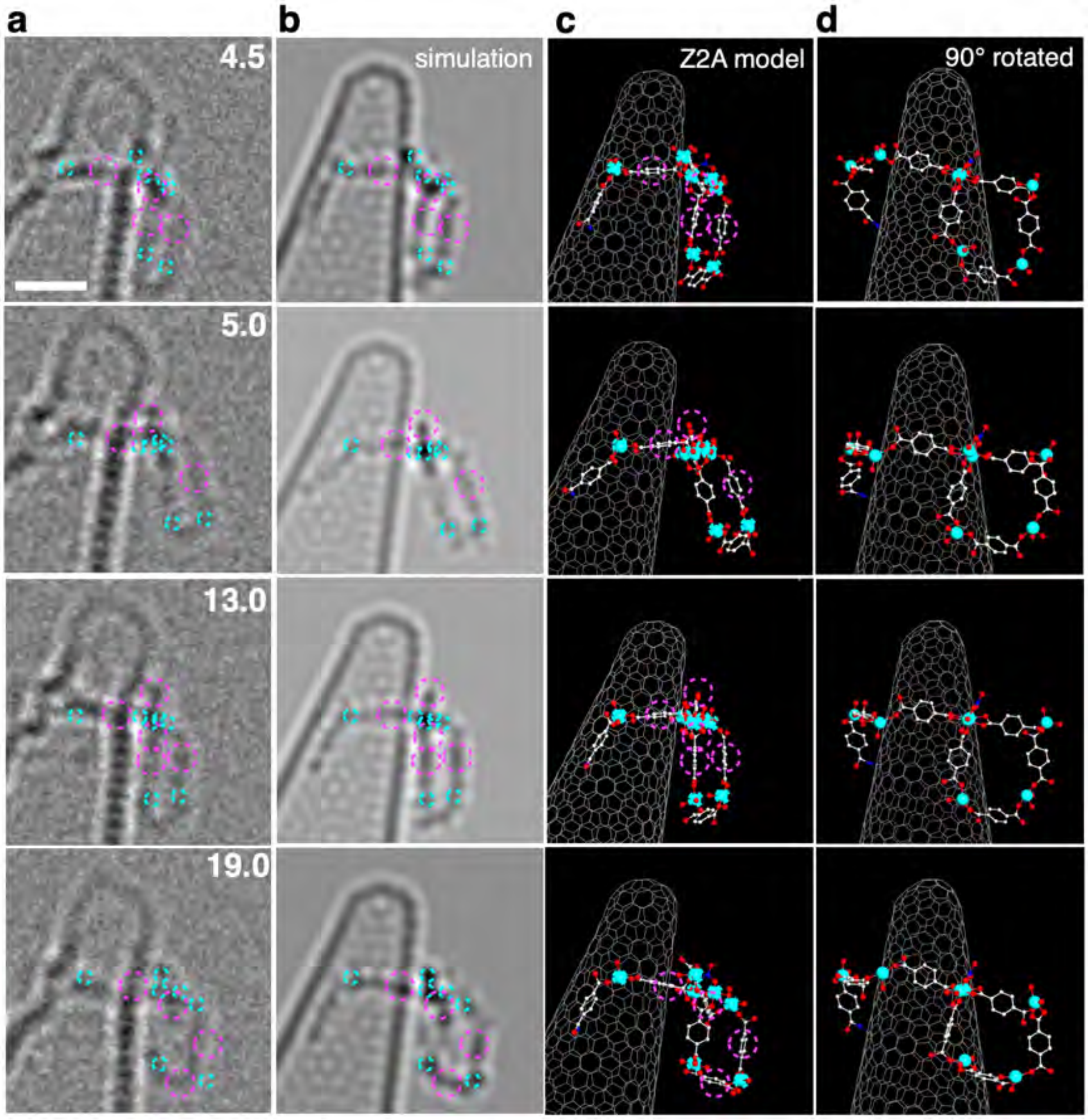

Supplementary Fig. 3. TEM image of a PNC taken from reaction of MOF-2 at 0.01 M of Zn^{2+} for 21 h (Fig. 3d–f) (methanol wash). (a) TEM image, (b) TEM simulation, (c) Z2A model, and (d) 90° rotated model. Positions of zinc atoms (light blue) and benzene rings parallel to the electron beam (purple) are circled. Scale bar is 1 nm.

Structure assignment of a PNC of I-MOF-5 on CNH (Fig. 6).

Here we further illustrate the assignment for the cube isolated from the I-BDC-5 synthesis mixture shown in Fig. 6. The 2-iodo-1,4-phenylene linkers rotated during observation, and we needed to modify the rotational angles for every image.

We first determined what images are worthy of analysis and what are not, by the use of a quantitative method to differentiate still images from blurred images by contrast analysis as shown in Fig. 6b.

To start the analysis, we focused on the six to seven dark iodine spots in the center of the image at 10.0 s and 34.5 s, which limits the number of regioisomers from 4096 to 14, whose structures are shown below. We examined several guess structures for each of the pictures at 10.0 s, 18.0 s, 25.5 s, 34.5 s, and 39.0 s, and concluded that

the one in a red box in Supplementary Fig. 4 is the only isomer that accounts for all five images with least difference between the experimental and simulated images (shown in the simulated images in Supplementary Fig. 6 such as 0.90 ± 0.49 Å).

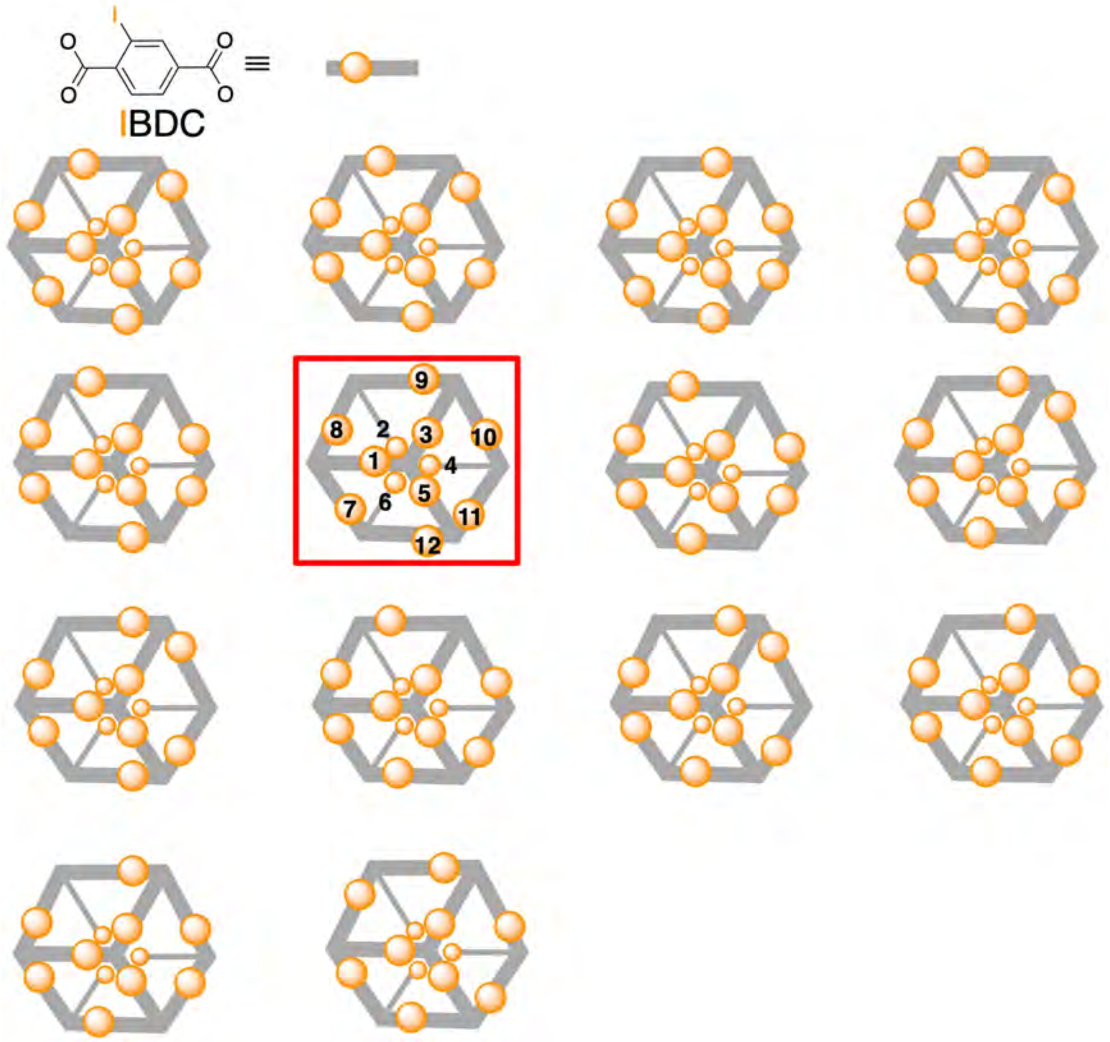

Supplementary Fig. 4. Schematic illustration of possible isomers for the cubic PNC of I-MOF-5 shown in Fig. 6 (which is the one in a red box).

We replaced one hydrogen with iodine in all 12 BDC molecules consisting of the cubic unit structure in MOF-5 crystal with eight Zn_3O node. By slightly rotating the whole structure and adjusting the orientation of phenyl group, we obtained different isomeric structure for PNC at different time.

We identified the iodine atoms in the simulation and the experimental images by using the iodine positions in the molecular model as reference, as we show an example of images at 10.0 s in Supplementary Fig. 5. We obtained the average deviation of the locations of the simulated iodine positions from the experimental ones in each image to be 0.90 ± 0.49 Å, 1.15 ± 0.75 Å, 1.23 ± 0.74 Å, 1.12 ± 0.61 Å, 1.15 ± 0.41 Å for 10.0, 18.0, 25.5, 34.5 and 39.0 s., respectively. In light of the spatial resolution of our TEM (1.0 Å) and the rotation of the IBDC linkers, we consider that the iodine locations determined in each 2-D projection serve as a reliable measure for

assigning spatial location of the iodine atom, and hence for the assigning a cube structure to the observed PNC.

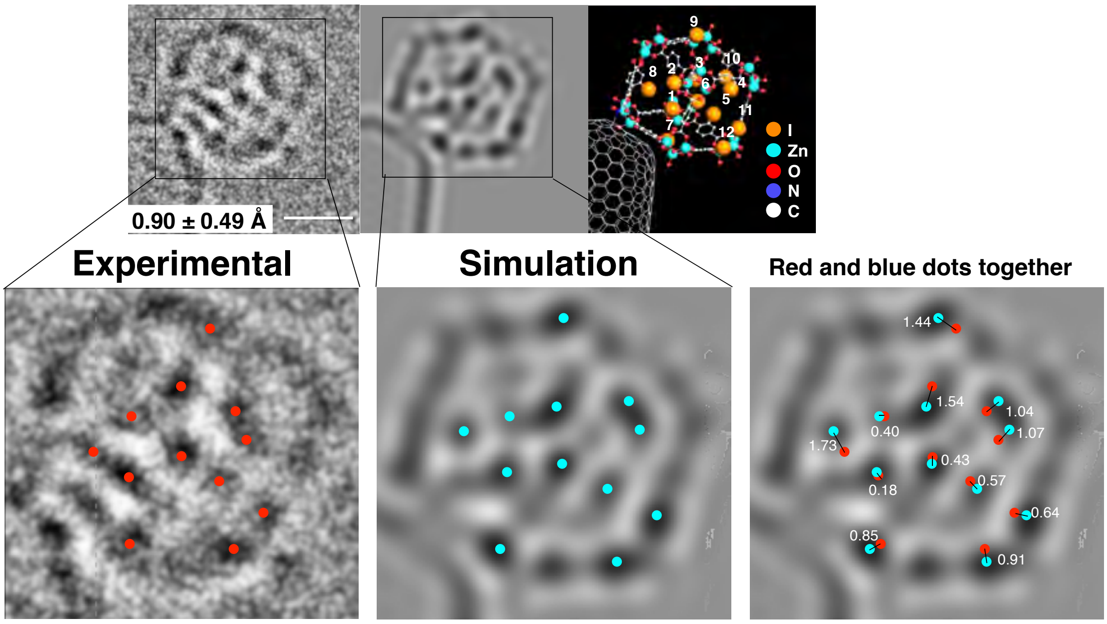

Supplementary Fig. 5. Measurement of average deviation in Å between simulation and TEM image shown in Fig. 6d and e. The centers of dark spots in TEM image are shown as red spots, and the centers of dark spots in the simulation image with light blue spots.

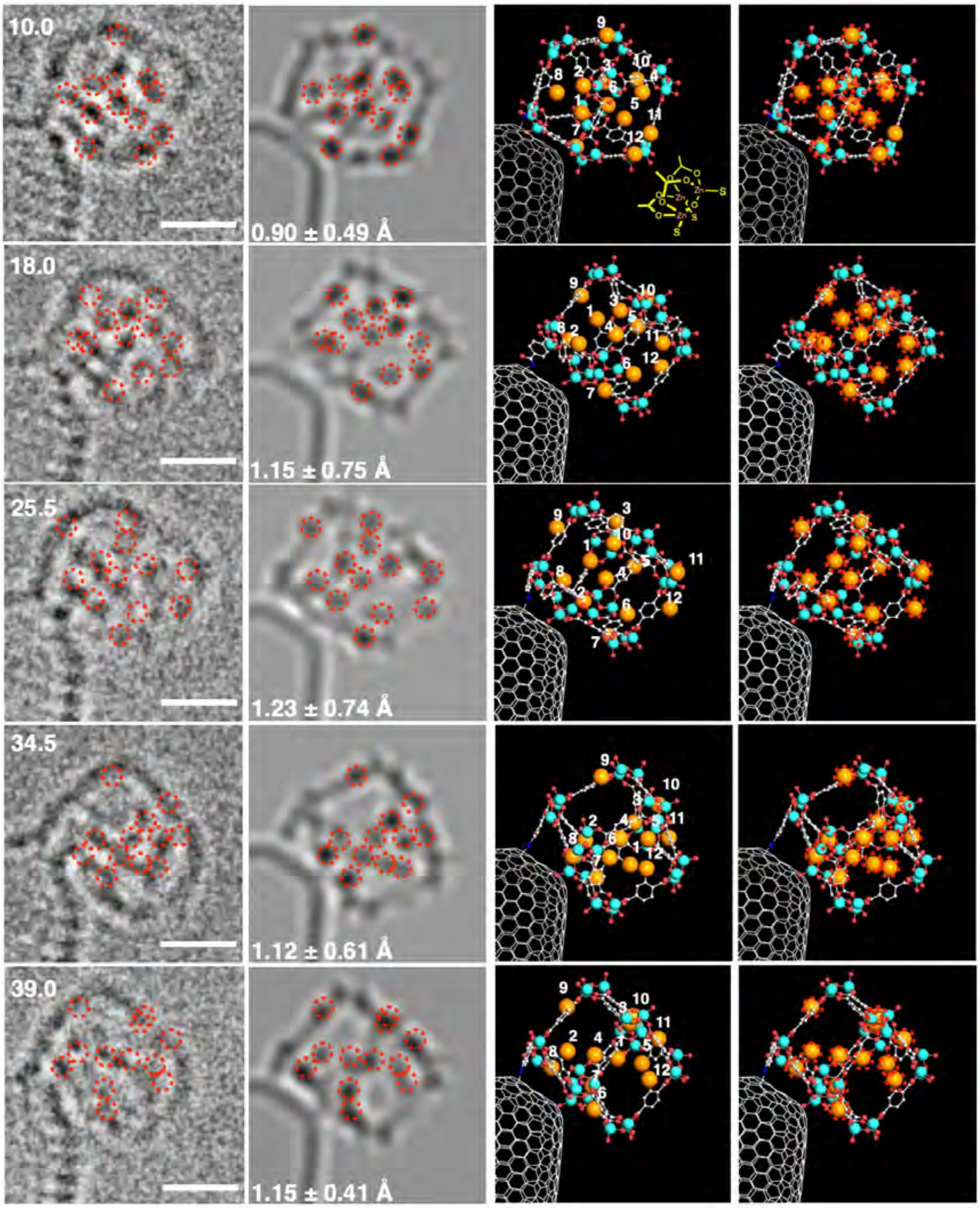

Supplementary Fig. 6. The TEM image of the cubic PNC of I-MOF-5 at 0.01 M of Zn^{2+} for 21 h (methanol wash) seen from five different direction, together with simulation and model (I orange, O red, Zn light blue, N blue, C grey, H white). The structural model of the corner is shown as an inset of 10.0 s molecular model (second from right). Numbers in the TEM images denote time in second after starting of the video recording. Average deviations of the locations of the simulated iodine positions from the experimental ones in each image are shown in the simulation. Scale bar is 1 nm.

3. Chemical Reactions
3-1. Preparation of BDC-CNH

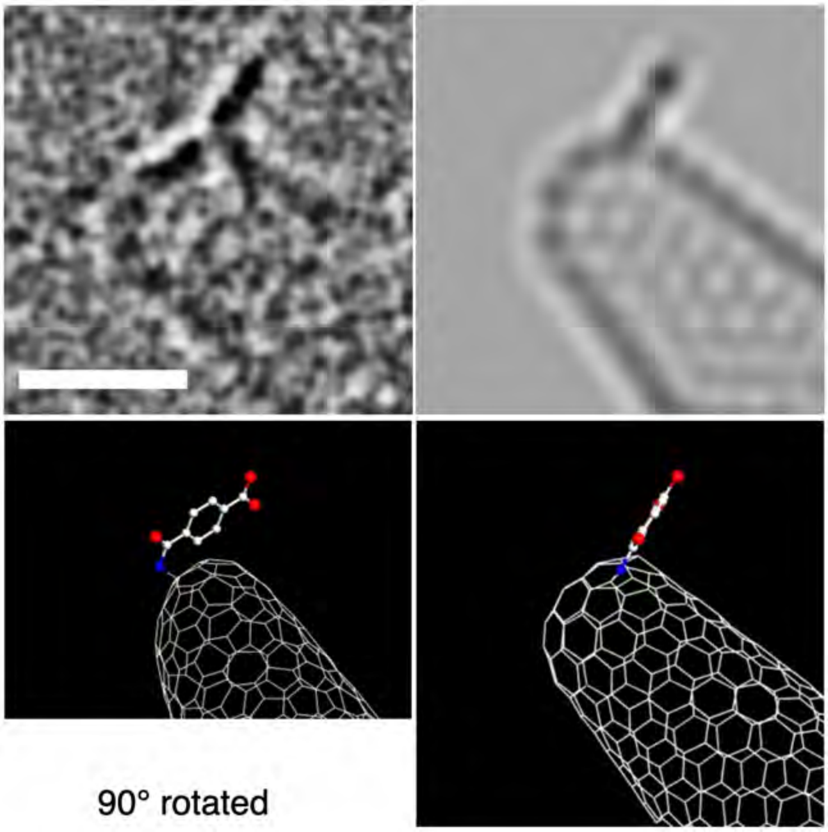

Supplementary Fig. 7. TEM image (top left), simulated TEM image (top light) and a molecular model (bottom) of BDC-CNH. The scale bar is 1 nm.

3-2. Preparation of MOF-2 and MOF-5 in the presence of BDC-CNH
3-2-1. MOF-2 in the presence of BDC-CNH
a. c(Zn^{2+}) = 0.05 M

$Zn(NO_3)_2$•$6H_2O$ (75 mg, 0.25 mmol) was mixed with terephthalic acid (21 mg, 0.13 mmol) and BDC-CNH (264.0 nmol / mg of -NH_2 groups, 0.95 mg, 1×10^{-3} eq vs Zn^{2+}) and stirred in DMF (5.0 mL) in a 10 mL flask for 21 h at 95 ˚C. After quickly cooling with a water bath at room temperature, the reaction mixture was filtered through a PTFE membrane filter (ADVANTEC, pore size: 100 nm) and washed with DMF (1 mL × 3) to obtain 32.0 mg of a gray powder (a mixture of MOF-2 crystals and BDC-CNH) after vacuum drying (60 Pa) for 12 h. The solid sample was analyzed by TEM and PXRD. Formation of MOF-2 was confirmed by PXRD measurement (Supplementary Fig. 8). The experiment without BDC-CNH was conducted in same condition. The yield is shown in Supplementary Table 1.

b. c(Zn^{2+}) = 0.1 M

$Zn(NO_3)_2$•$6H_2O$ (149 mg, 0.50 mmol) was mixed with terephthalic acid (42 mg, 0.25 mmol) and BDC-CNH (264.0 nmol / mg of -NH_2 groups, 0.95 mg, 5×10^{-4} eq vs Zn^{2+}) and stirred in DMF (5.0 mL) in a 10 mL flask for 21 h at 95 ˚C. After quickly cooling with a water bath at room temperature, the reaction mixture was filtered through PTFE membrane filter (ADVANTEC, pore size: 100 nm) and washed with DMF (1 mL × 3) to obtain 59.3 mg of a gray powder (a mixture of MOF-2

crystals and BDC-CNH) after vacuum drying (60 Pa) for 12 h. The solid sample was analyzed by TEM and PXRD. Formation of MOF-2 was confirmed by PXRD measurement (Supplementary Fig. 8). The experiment without BDC-CNH was conducted in same condition. The yield is shown in Supplementary Table 1.

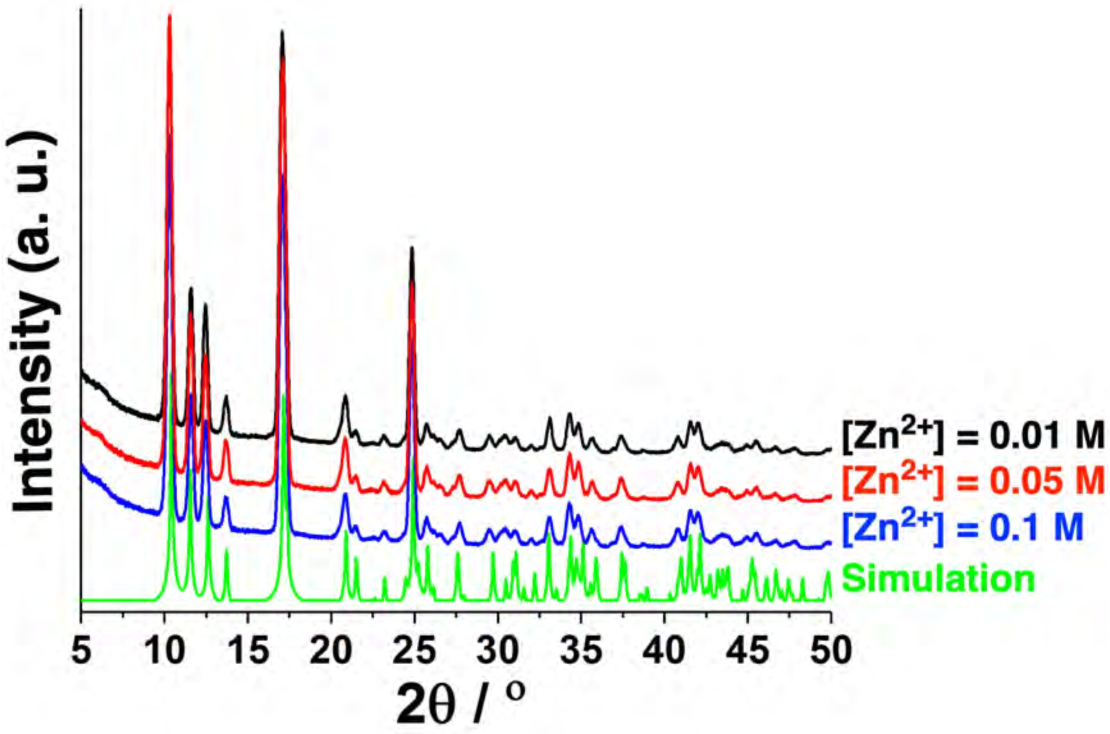

Supplementary Fig. 8. PXRD patterns of MOF-2 prepared with BDC-CNH at different Zn^{2+} concentrations (black, 0.01 M of Zn^{2+}; red, 0.05 M; blue, 0.1 M). The reaction time is 21 h. A simulation pattern from single crystal data of MOF-2[2] is shown in green. The diffraction patterns match well with the simulation pattern, which indicates that the presence of BDC-CNH in MOF-2 reaction system does not hamper the formation of MOF-2.

3-2-2. MOF-5 in the presence of BDC-CNH

a. c(Zn^{2+}) = 0.05 M

$Zn(NO_3)_2$•$6H_2O$ (75 mg, 0.25 mmol) was mixed with terephthalic acid (21 mg, 0.13 mmol) and BDC-CNH (264.0 nmol / mg of-NH_2 groups, 0.95 mg, 1×10^{-3} eq vs Zn^{2+}) and stirred in DMF (5.0 mL) in a 10 mL flask for 21 h at 120 ˚C. After quickly cooling with a water bath at room temperature, the reaction mixture was filtered through PTFE membrane filter (ADVANTEC, pore size: 100 nm) and washed with DMF (1 mL × 3) to obtain 46.9 mg of gray powder (a mixture of MOF-5 crystals and BDC-CNH) after vacuum drying (60 Pa) for 12 h. The solid sample was analyzed by TEM and PXRD. Formation of MOF-5 crystal was confirmed by PXRD measurement (Supplementary Fig. 9). The experiment without BDC-CNH was conducted in same condition. The yield is shown in Supplementary Table 1.

b. c(Zn^{2+}) = 0.1 M

$Zn(NO_3)_2$•$6H_2O$ (149 mg, 0.50 mmol) was mixed with terephthalic acid (42 mg, 0.25 mmol) and BDC-CNH (264.0 nmol / mg of -NH_2 groups, 0.95 mg, 5×10^{-4}

eq vs Zn^{2+}) and stirred in DMF (5.0 mL) in a 10 mL flask for 21 h at 120 ˚C. After quickly cooling with a water bath at room temperature, the reaction mixture was filtered through PTFE membrane filter (ADVANTEC, pore size: 100 nm) and washed with DMF (1 mL × 3) to obtain 72.7 mg of a gray powder (a mixture of MOF-5 and BDC-CNH) after vacuum drying (60 Pa) for 12 h. The solid sample was analyzed by TEM and PXRD. Formation of MOF-5 crystals was confirmed by PXRD measurement (Supplementary Fig. 9). The experiment without BDC-CNH was conducted in same condition. The yield is shown in Supplementary Table 1.

The yield of MOF-2 and MOF-5 is summarized in Supplementary Table 1. We removed absorbed DMF by immersing MOF crystals into $CHCl_3$ for 48 h followed by vacuum drying (60 Pa) for 12 hour three times according to the literature[3]. The yield of MOF-2 and MOF-5 crystals did not change much regardless of the presence of BDC-CNH, suggesting that BDC-CNH does not hamper or enhance the crystal formation process.

Supplementary Table 1. Yield of MOF-2-DMF and MOF-5 at different Zn^{2+} concentrations.

Conditions	0.01 M Zn^{2+}	0.05 M Zn^{2+}	0.1 M Zn^{2+}
MOF-2 (21 h)	53%	54%	47%
BDC-CNH + MOF-2 (21 h)	55%	55%	46%
MOF-5 (21 h)	84%	79%	74%
BDC-CNH + MOF-5 (21 h)	82%	69%	75%

Supplementary Table 2. Summary of number of PNCs with different structure of MOF-2, MOF-5 and I-MOF-5 at different Zn^{2+} concentrations.

Conditions	Lower ordered	Higher ordered	Ratio of higher ordered
0.01 M Zn^{2+} MOF-2 (21 h)	56	0	0%
0.05 M Zn^{2+} MOF-2 (21 h)	48	0	0%
0.1 M Zn^{2+} MOF-2 (21 h)	45	0	0%
0.01 M Zn^{2+} MOF-5 (21 h)	32	4	11%
0.05 M Zn^{2+} MOF-5 (21 h)	33	0	0%
0.1 M Zn^{2+} MOF-5 (21 h)	73	0	0%
0.01 M Zn^{2+} I-MOF-5 (21 h)	41	13	24%

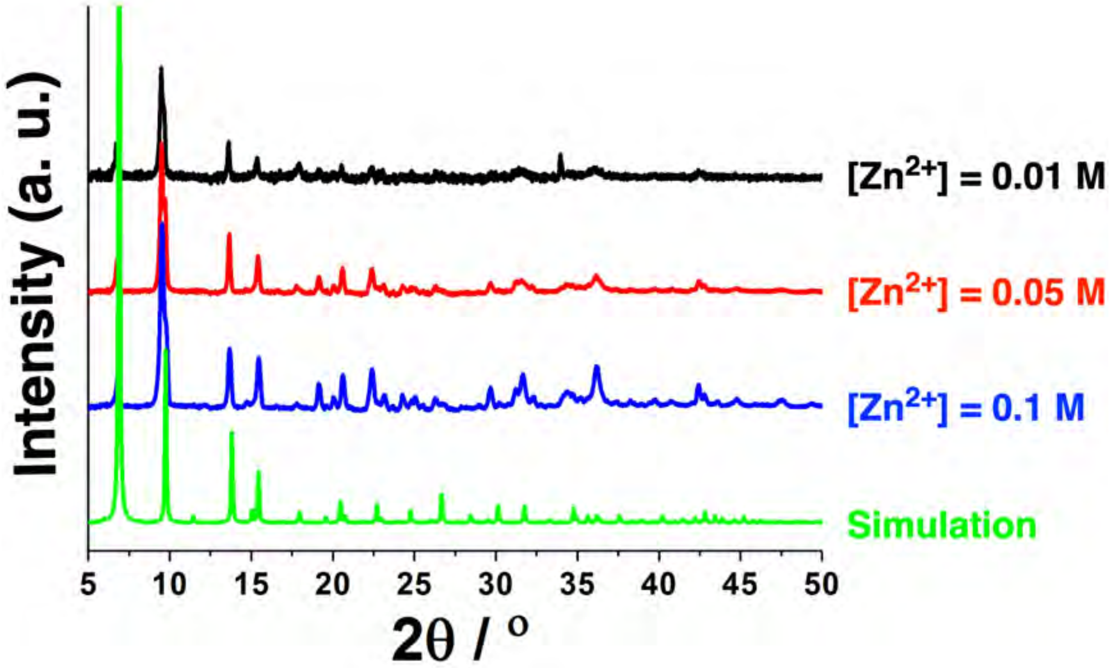

Supplementary Fig. 9. PXRD patterns of BDC-CNH + MOF-5 with different Zn^{2+} concentration (black, 0.01 M of Zn^{2+}; red, 0.05 M; blue, 0.1 M). The reaction time is 21 h. A simulation pattern from single crystal data of MOF-5[4] is shown in green. The diffraction patterns match well with the simulation pattern.

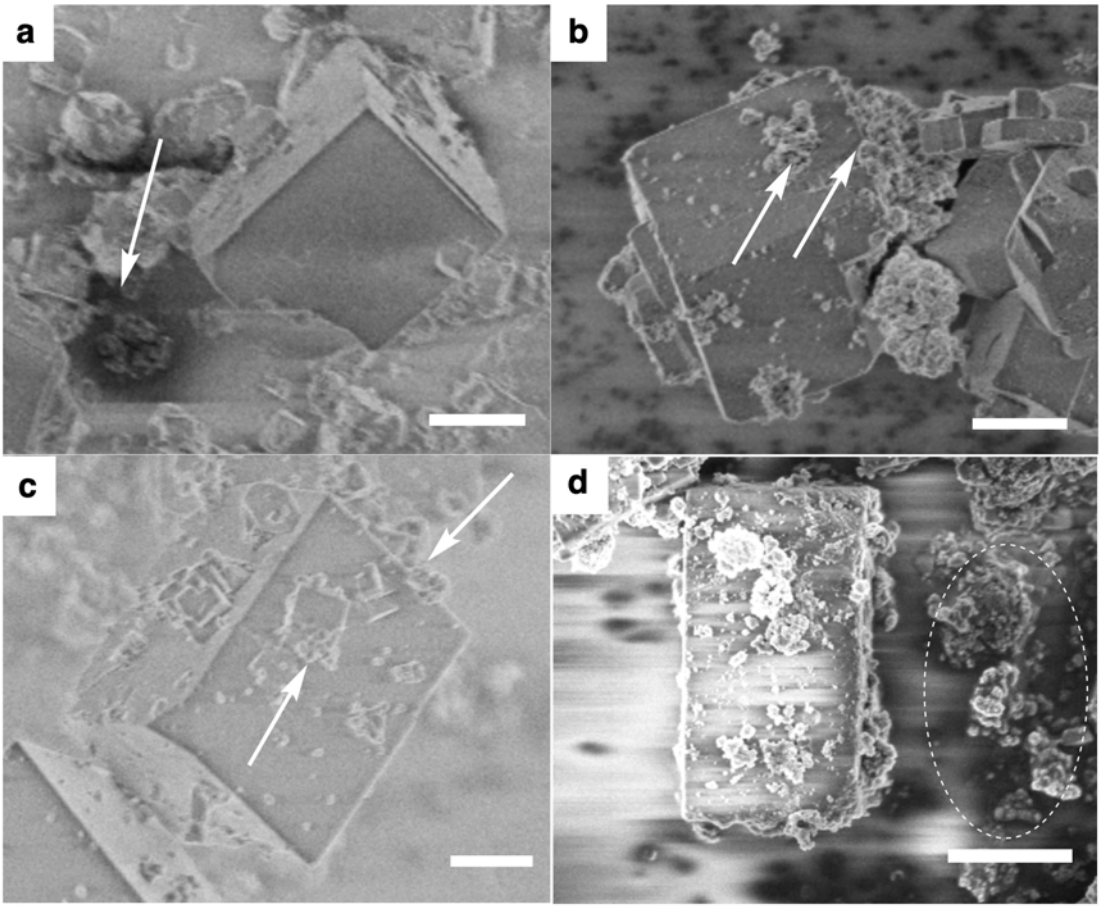

Supplementary Fig. 10. SEM images of isolated MOF-5 prepared in the presence of pristine CNH (a) and BDC-CNH with Zn^{2+} concentration = (b) 0.1, (c) 0.05 and (d) 0.01 M. In image a, the surface of the pristine CNH remains clean. In images b-d, however, agglomerates of BDC-CNH particles stick to the surface of MOF-5, suggesting that the BDC-CNH has high affinity to the MOF-5 surface because of the BDC appendage. For SMART-EM studies of PNCs, the CNH agglomerates of BDC-CNH particles circled in image d were analyzed after transfer to a TEM grid. Scale bars: 2 μm for a and b, 1 μm for c and d.

3-2-3. Time-dependent experiments of MOF-5 formation in the presence of BDC-CNH

a. 0 h

The pre-heated DMF solution (2.5 mL) of $Zn(NO_3)_2$•$6H_2O$ (15.0 mg, 0.050 mmol) was mixed with the pre-heated DMF solution (2.5 mL) of terephthalic acid (4.2 mg, 0.025 mmol) and BDC-CNH (264.0 nmol / mg of -NH_2 groups, 0.95 mg, 5 × 10^{-3} eq vs Zn^{2+}) in a 10 mL flask at 120 ˚C. After quickly cooling with a water bath at room temperature, the mixture was filtered through a PTFE membrane filter (ADVANTEC, pore size: 100 nm) and washed with DMF (1 mL × 3) to obtain 1.1 mg of black powder after vacuum drying (60 Pa) for 12 h. The solid sample was analyzed by TEM.

b. 0.5–16 h

$Zn(NO_3)_2$•$6H_2O$ (15.0 mg, 0.050 mmol) was mixed with terephthalic acid (4.2 mg, 0.025 mmol) and BDC-CNH (264.0 nmol / mg of- NH_2 groups, 0.95 mg, 5 × 10^{-3}

eq vs Zn^{2+}) and stirred in DMF (5.0 mL) in a 10 mL flask for different reaction time (0.5 h, 4 h, 6 h, 8 h, 12 h, and 16 h) at 120 ˚C. After quickly cooling with a water bath at room temperature, the reaction mixture was filtered through PTFE membrane filter (ADVANTEC, pore size: 100 nm) and washed with DMF (1 mL × 3) to obtain black powder (0.5 h, 2.6 mg; 4 h, 9.2 mg; 6 h, 9.3 mg; 8 h, 9.1 mg; 12 h, 9.3 mg; 16 h, 9.2 mg) after vacuum drying (60 Pa) for 12 h. The solid sample was analyzed by TEM and PXRD.

A part of the reaction mixture (1.2 mL) was diluted with water (4.8 mL) and the pH value was monitored by pH paper. The pH value increased by elongating reaction time. A pH meter do not give an accurate pH value of DMF/water mixture but showed the same trend as pH paper.
pH paper: 0 h, pH 3; 0.5 h, pH 6; 4 h, pH 7; 6 h, pH 8; 8 h, pH 8; 12 h, pH 9; 16 h, pH 9; 21 h, pH 9.
pH meter: 0 h, pH 3.69; 0.5 h, pH 4.14; 4 h, pH 5.44; 6 h, pH 5.47; 8 h, pH 6.45; 12 h, pH 6.90; 16 h, pH 6.91; 21 h, pH 7.02.

Supplementary Table 3. Summary of number of PNCs with different structure of MOF-5 with different reaction time.

Conditions	Lower ordered	Higher ordered	Ratio of higher ordered
0.01 M Zn^{2+} MOF-5 (0 h)	52	0	0%
0.01 M Zn^{2+} MOF-5 (0.5 h)	45	2	4%
0.01 M Zn^{2+} MOF-5 (4 h)	128	4	3%
0.01 M Zn^{2+} MOF-5 (6 h)	128	5	4%
0.01 M Zn^{2+} MOF-5 (8 h)	63	4	6%
0.01 M Zn^{2+} MOF-5 (12 h)	53	5	9%
0.01 M Zn^{2+} MOF-5 (16 h)	62	7	10%
0.01 M Zn^{2+} MOF-5 (21 h)	32	4	11%

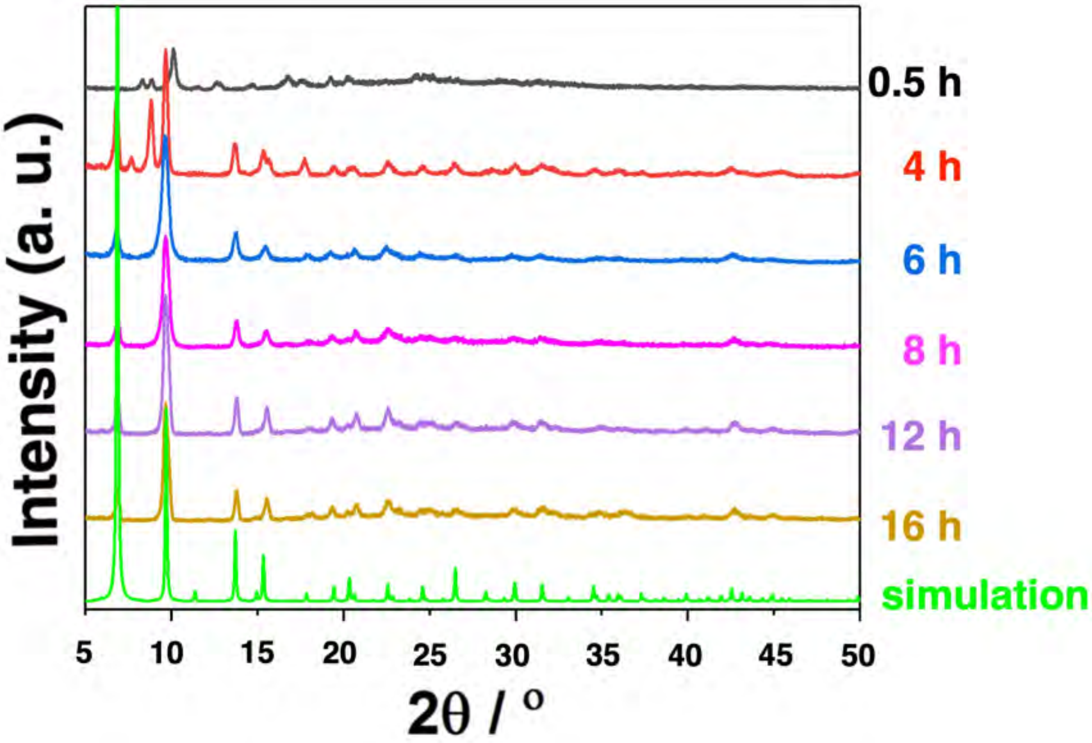

Supplementary Fig. 11. PXRD patterns of BDC-CNH + MOF-5 with different reaction time (black, 0.5 h; red, 4 h; blue, 6 h; pink, 8 h; purple, 12 h; brown, 16 h) at 0.01 M concentration of Zn^{2+}. A simulation pattern from single crystal data of MOF-5[4] is shown in green.

3-2-4. I-MOF-5

$Zn(NO_3)_2 \bullet 6H_2O$ (30.0 mg, 0.10 mmol) was mixed with 2-iodoterephthalic acid (15.0 mg, 0.050 mmol) and BDC-CNH (264.0 nmol/mg of $-NH_2$ groups, 1.9 mg, 5×10^{-3} eq vs Zn^{2+}) and stirred in DMF (10.0 mL) in a 20 mL flask for 21 h at 120 ˚C. After quickly cooling with a water bath at room temperature, the reaction mixture was filtered through a PTFE membrane filter (ADVANTEC, pore size: 100 nm) and washed with DMF (1 mL × 3). 11.4 mg of black powder (mixture of I-MOF-5 crystals and BDC-CNH) was obtained after vacuum drying (60 Pa) for 12 h. The solid sample was analyzed by TEM and PXRD. Formation of I-MOF-5 was confirmed by PXRD measurement (Supplementary Fig. 12).

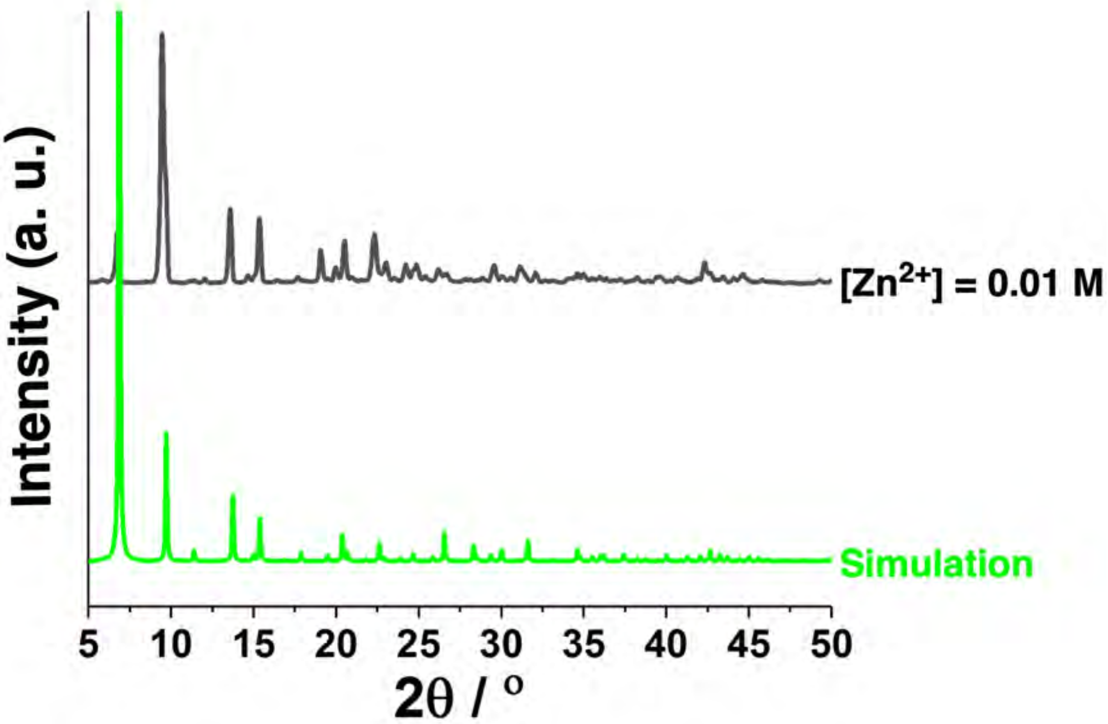

Supplementary Fig. 12. PXRD pattern of I-MOF-5 prepared with BDC-CNH of I-MOF-5 at 0.01 M concentration of Zn^{2+}. The diffraction patterns match well with the diffraction pattern of I-MOF-5 reported in the literature[5] shown in green.

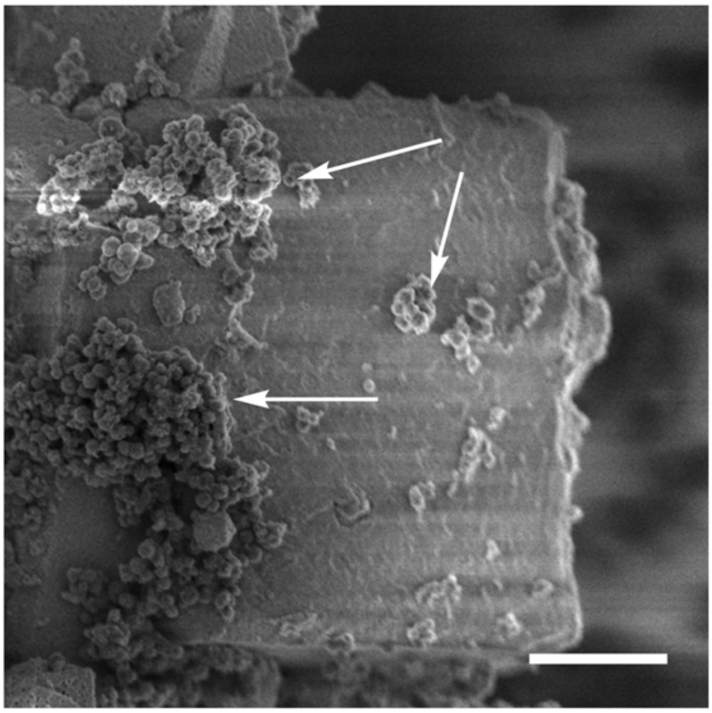

Supplementary Fig. 13. SEM image of isolated I-MOF-5 prepared in the presence of BDC-CNH. A number of BDC-CNH particles (indicated by arrows) are attached to the surface of the I-MOF-5 crystal because of its affinity to the MOF crystals. Scale bar is 1 μm.

3-3. Preparation of samples for SEM and SMART-EM

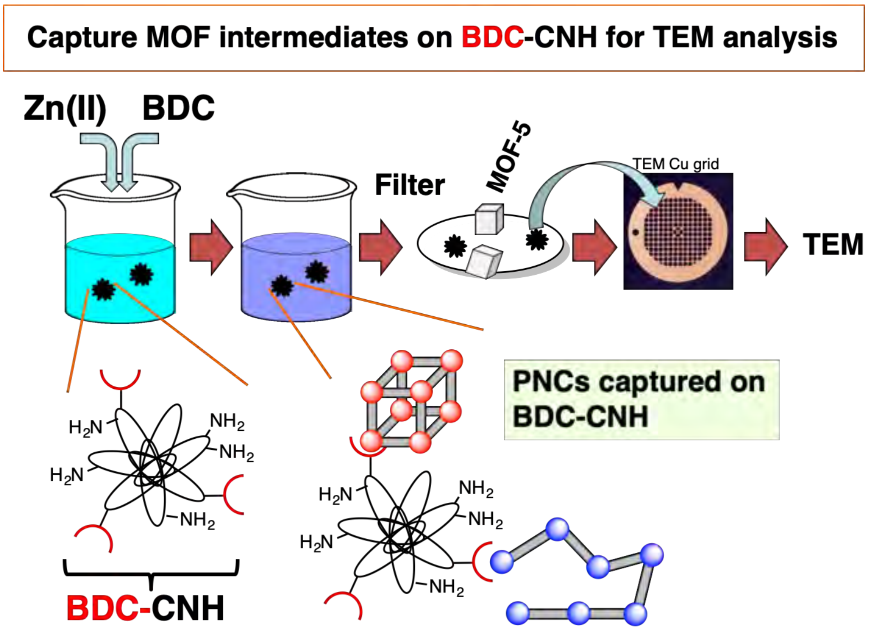

Supplementary Fig. 14. Schematic illustration of the procedure for in situ trapping of PNC on BDC-CNH fishhook for SMART-EM imaging.

3-4. Size distribution of PNCs in solution studied by DLS analysis of MOF-5 synthesis at c(Zn^{2+}) = 0.01 M

DLS sample was prepared by mixing $Zn(NO_3)_2$•$6H_2O$ (30.0 mg, 0.1 mmol) and H_2BDC (8.3 mg, 0.05 mmol) in 10 mL MDF in 20 mL flask. The sample was stirred (400 rpm) in room temperature for 30 min before heating. The time 0 is the time start heating. The oil bath is pre-heated before the flask is immersed. Upon heating the mixture at 120 °C, fine crystals started to form after approximately 30 min, and the yield of the crystals stopped to increase after approximately 4 h. An aliquot was removed periodically with a syringe, filtered with a Millipore filter (200 nm pore size), and the filtrate was analyzed at room temperature. Particle number average data are shown in Supplementary Fig. 15.

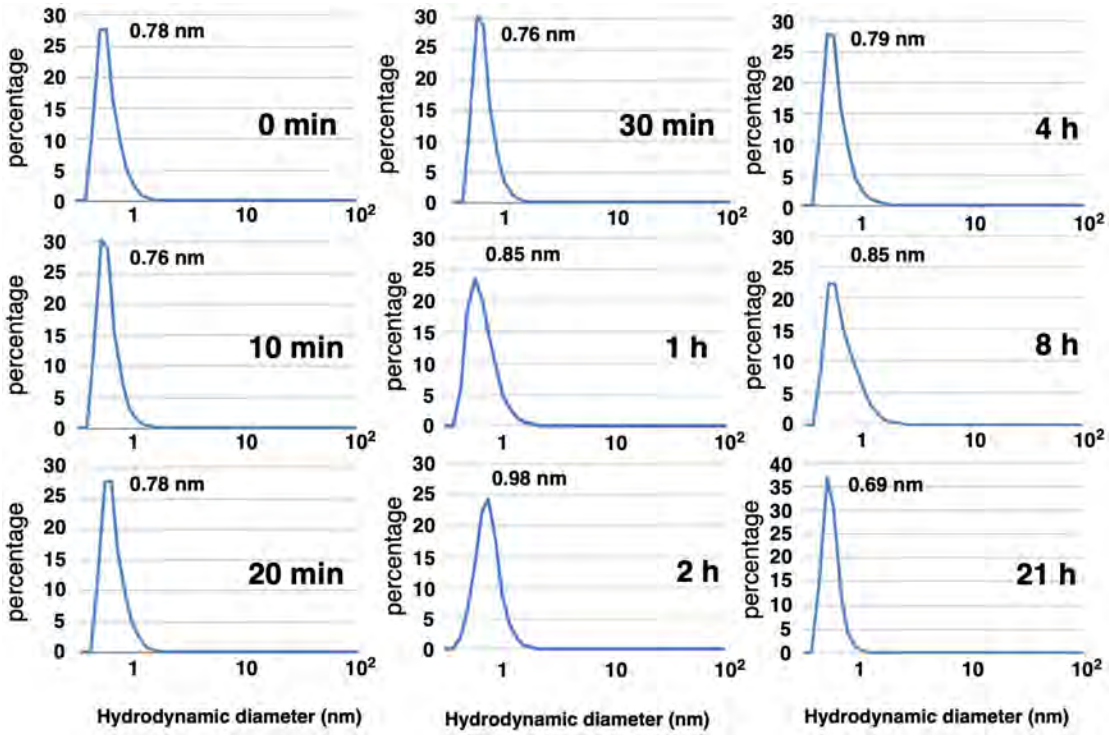

Supplementary Fig. 15. Number-averaged size distribution of PNCs in filtered MOF-5 reaction mixture obtained by DLS (0.01 M concentration of Zn^{2+}).

3-5. Size distribution of PNCs in solution studied by DLS analysis of MOF-2 synthesis at c(Zn^{2+}) = 0.01 M

DLS sample was prepared by mixing $Zn(NO_3)_2$•$6H_2O$ (30.0 mg, 0.1 mmol) and H_2BDC (8.3 mg, 0.05 mmol) in 10 mL MDF in 20 mL flask. The sample was stirred (400 rpm) in room temperature for 30 min before heating. The time 0 is the time start heating. The oil bath is pre-heated before the flask is immersed. Upon heating the mixture at 95 °C, fine crystals started to form after approximately 3 h. An aliquot was removed periodically with a syringe, filtered with a Millipore filter (200 nm pore size), and the filtrate was analyzed at room temperature. Particle number average data are shown in Supplementary Fig. 16.

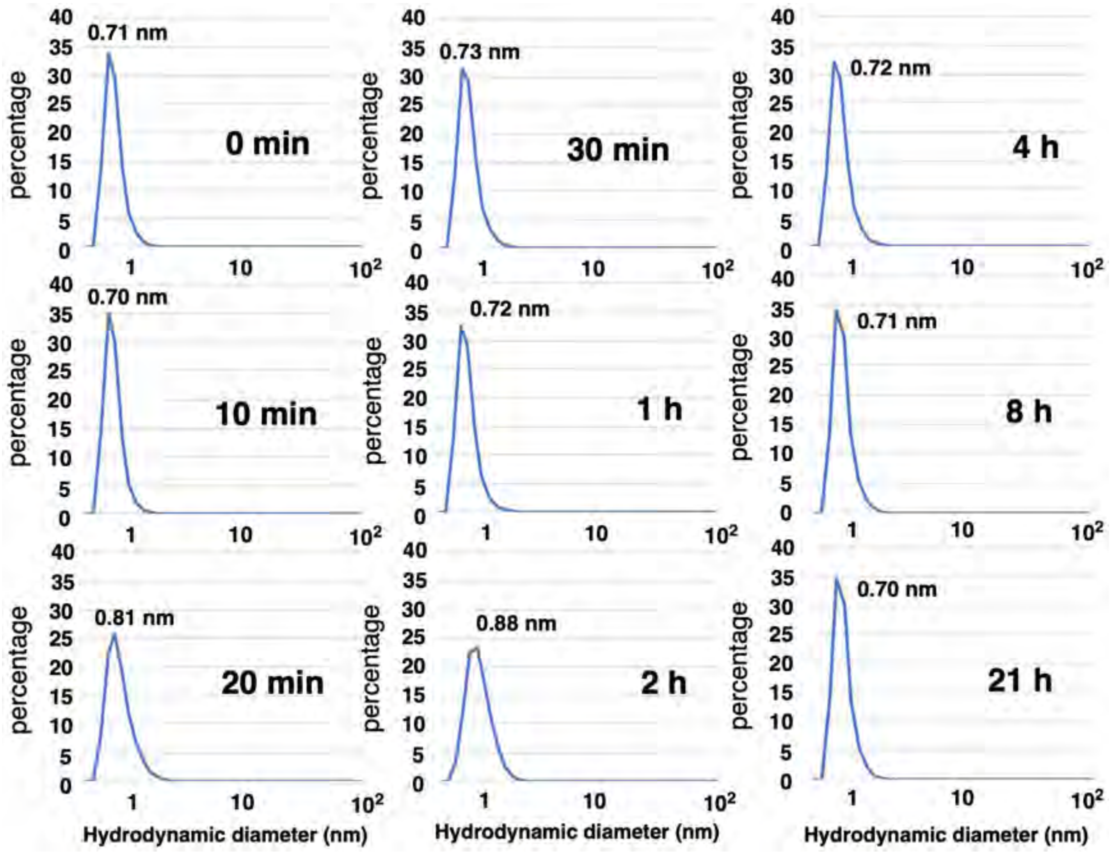

Supplementary Fig. 16. Number-averaged size distribution of PNCs in filtered MOF-2 reaction mixture obtained by DLS (0.01 M concentration of Zn^{2+}).

3-6. Size distribution of PNC on BDC-CNH studied by TEM

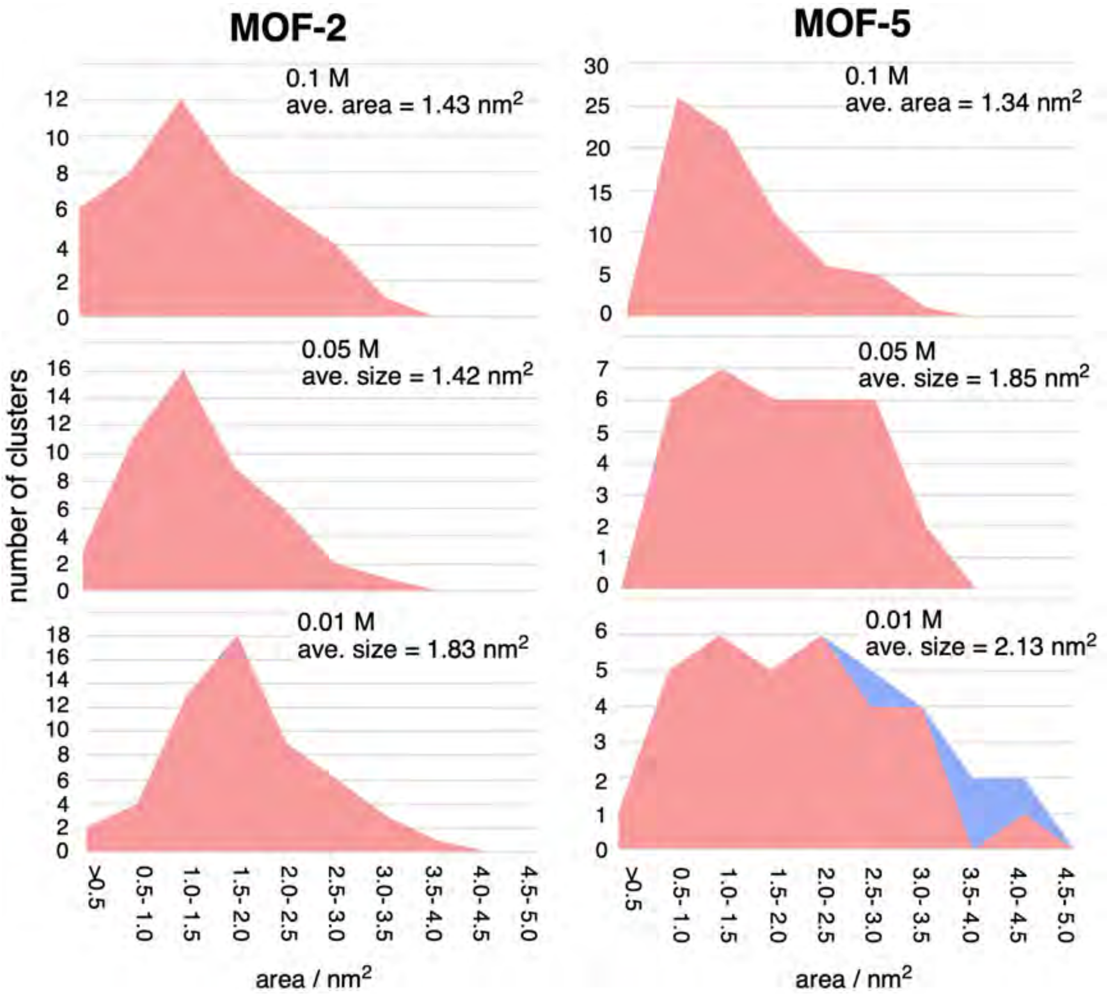

Supplementary Fig. 17. Size distribution (as analyzed for approximate projection area) of PNCs of MOF-2 and MOF-5 under different Zn^{2+} concentration (methanol wash). The reaction time is 21 h. Red indicates the lower order PNCs while blue indicates the cube and cube-like PNCs, which were found only in the MOF-5 synthesis under low concentration of 0.01 M.

Supplementary Figures

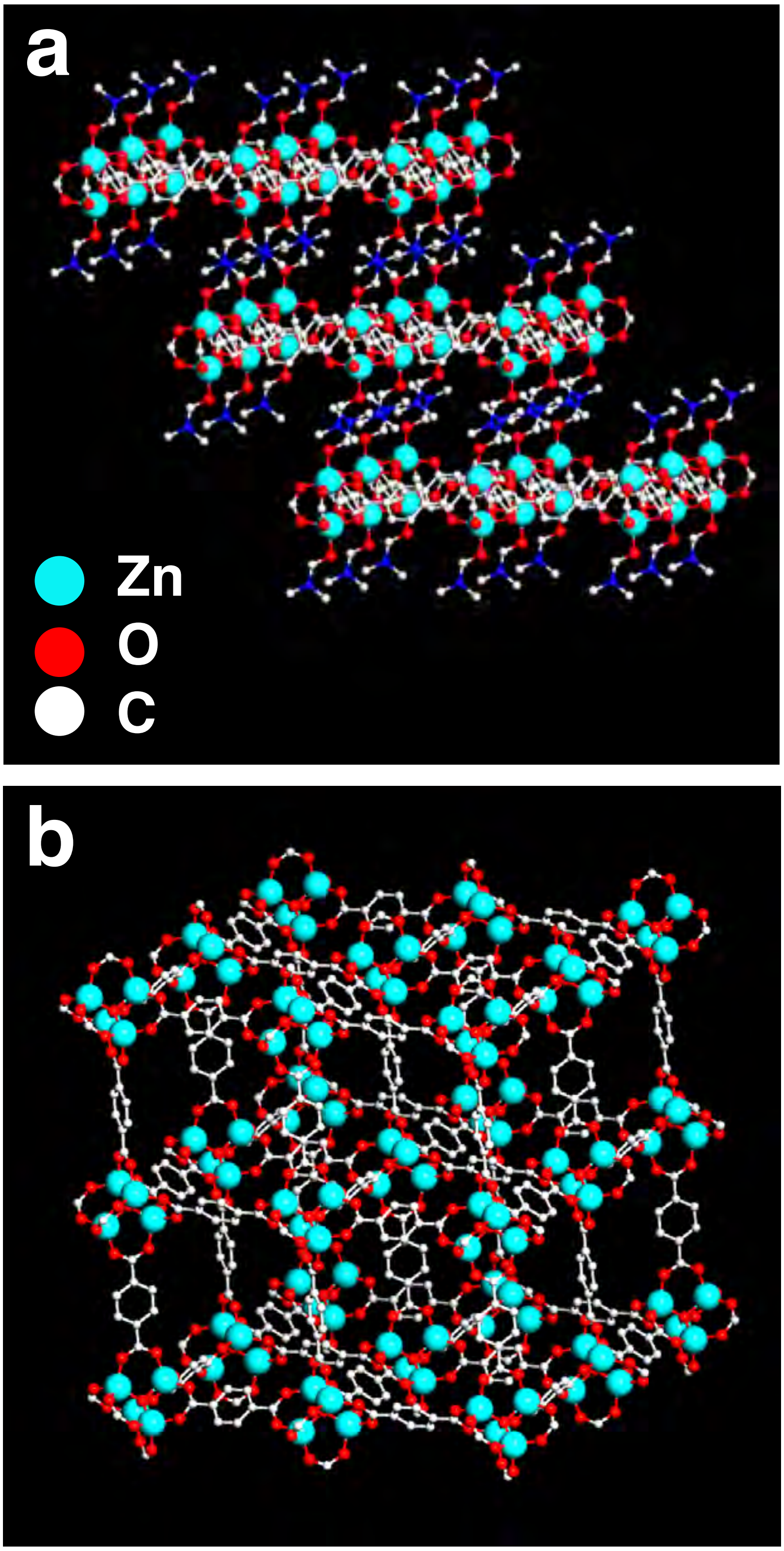

Supplementary Fig. 18. Crystal structure of MOF-2-DMF (a) and MOF-5 (b) shown as Z2A model. Hydrogen atoms are removed for clarity.

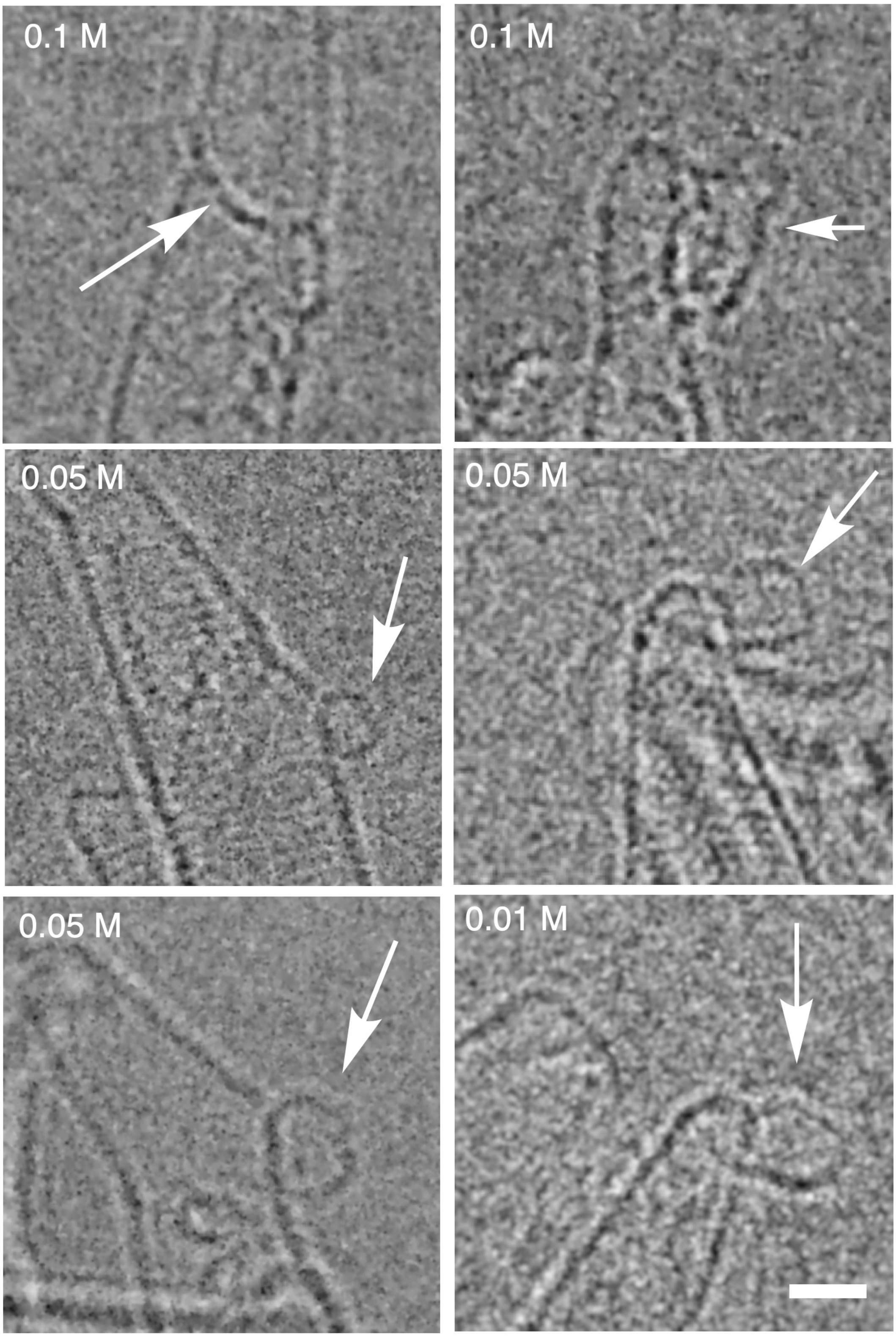

Supplementary Fig. 19. Representative TEM images of lower order PNCs of MOF-2 at different concentration of Zn^{2+} for 21 h (methanol wash) found in the reaction mixture. PNCs were identified by their motions while the CNH remained static. Concentration of Zn^{2+} is shown for each image. Scale bar is 1 nm.

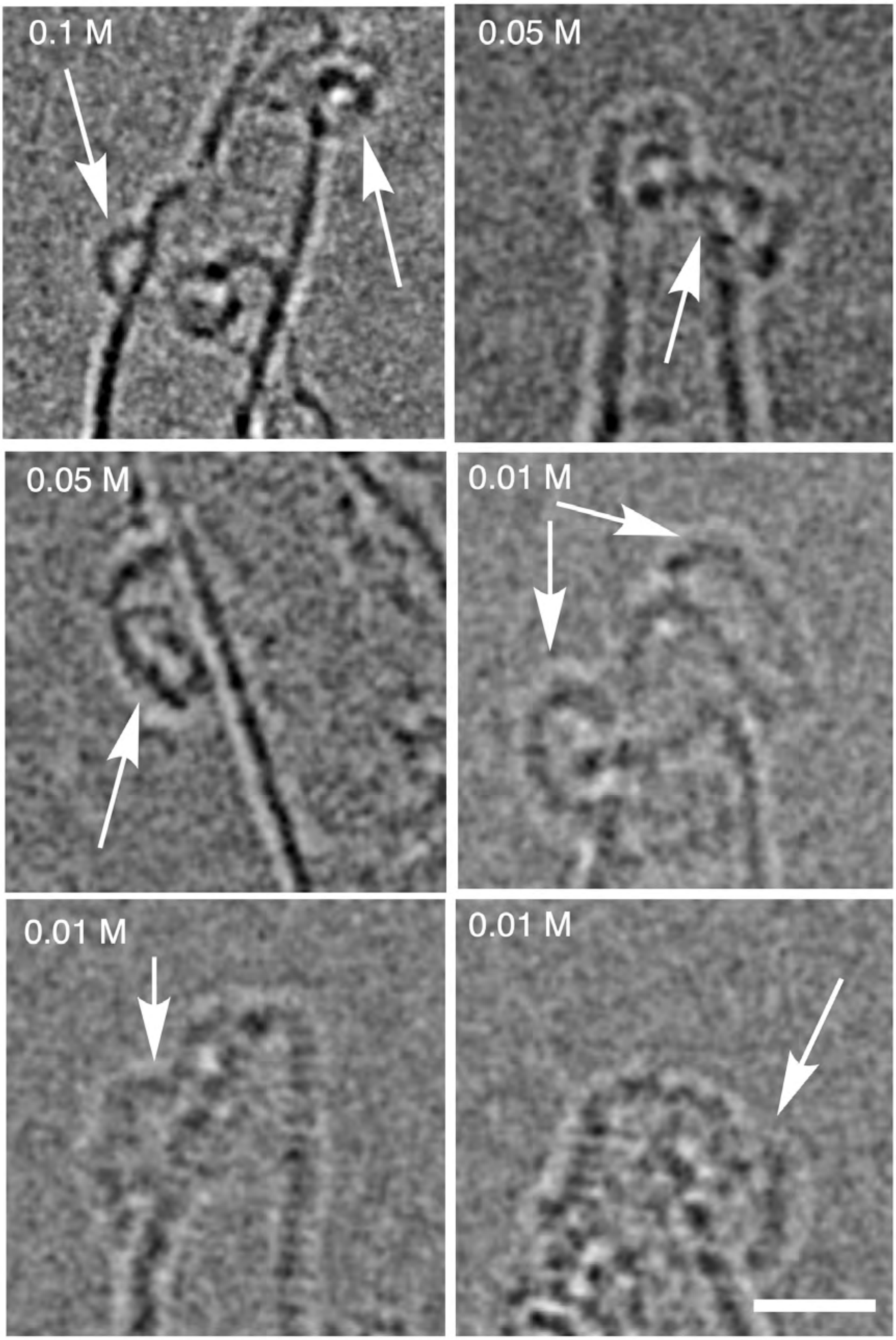

Supplementary Fig. 20. Representative TEM images of the lower order PNCs of MOF-5 at different concentration of Zn^{2+} for 21 h (methanol wash). PNCs were identified by their motions while the CNH remained static. Concentration of Zn^{2+} is shown for each image. Scale bar is 1 nm.

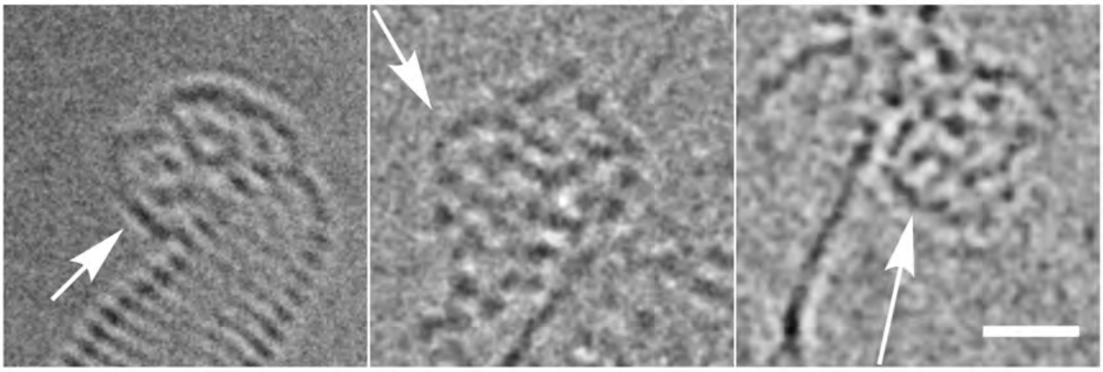

Supplementary Fig. 21. Representative TEM images of cube and cube-like 3-D PNCs of MOF-5 at 0.01 M of Zn^{2+} for 21 h (methanol wash). PNCs were identified by their motions while the CNH remained static. Scale bar is 1 nm.

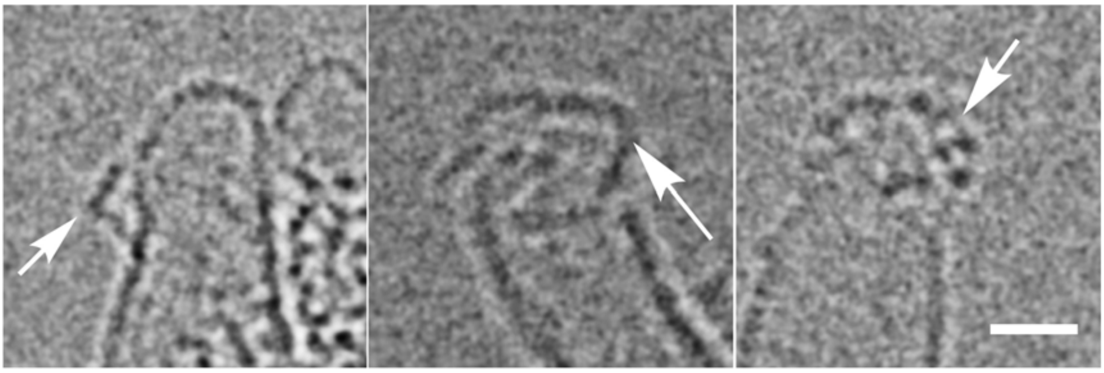

Supplementary Fig. 22. Representative TEM images of lower order PNCs of I-MOF-5 at 0.01 M of Zn^{2+} for 21 h (methanol wash). PNCs were identified by their motions while the CNH remained static. Scale bar is 1 nm.

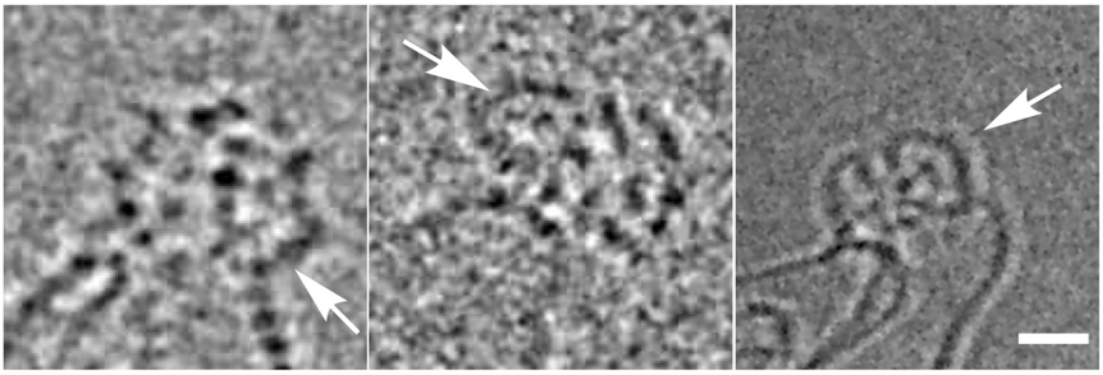

Supplementary Fig. 23. Representative TEM images of cube and cube-like 3-D PNCs of I-MOF-5 at 0.01 M of Zn^{2+} for 21 h (methanol wash). PNCs were identified by their motions while the CNH remained static. Scale bar is 1 nm.

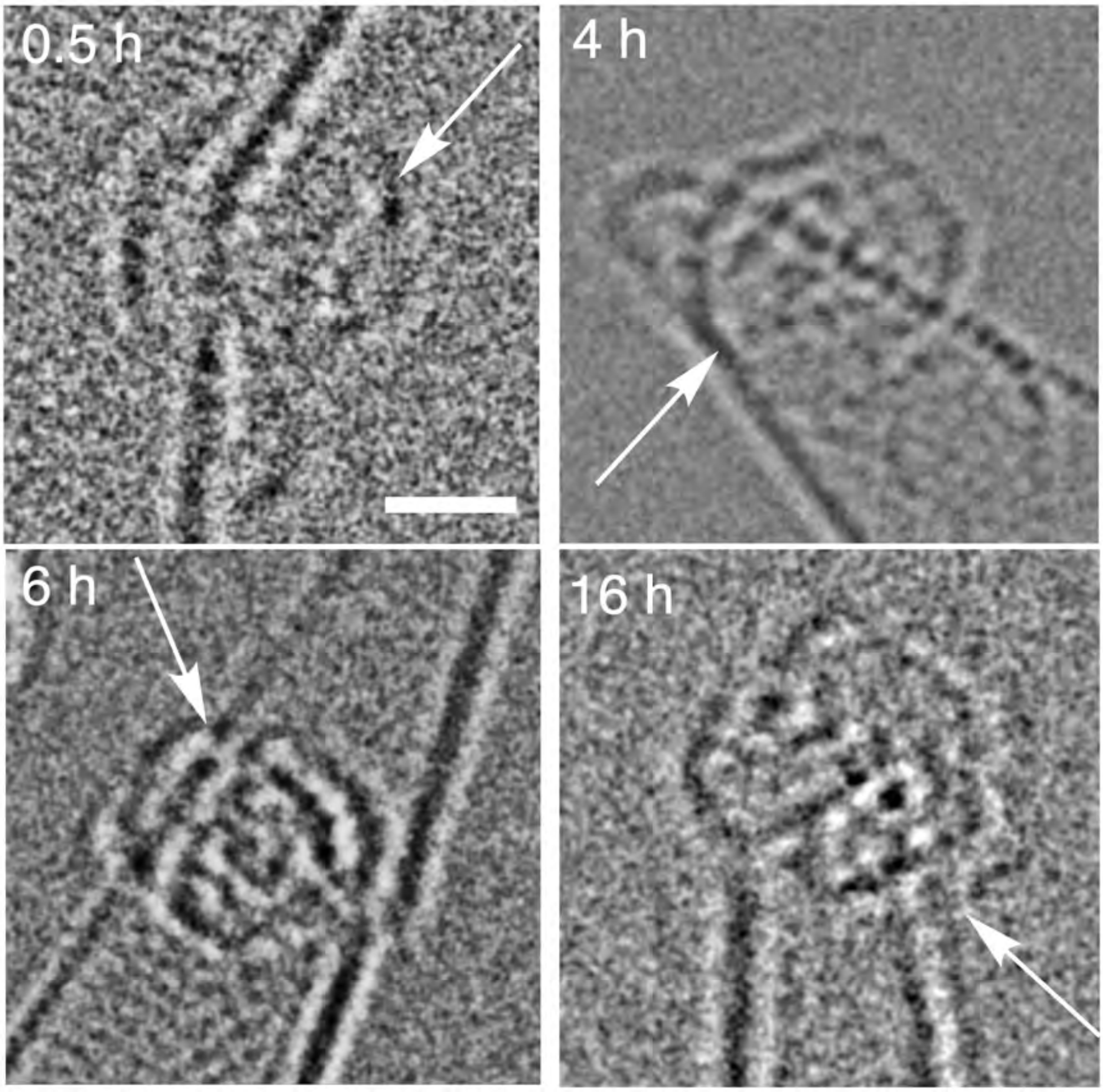

Supplementary Fig. 24. Representative TEM images of cube and cube-like 3-D PNCs of MOF-5 at 0.01 M of Zn^{2+} for different reaction time (methanol wash). PNCs were identified by their motions while the CNH remained static. Reaction time is shown for each image. Scale bar is 1 nm.

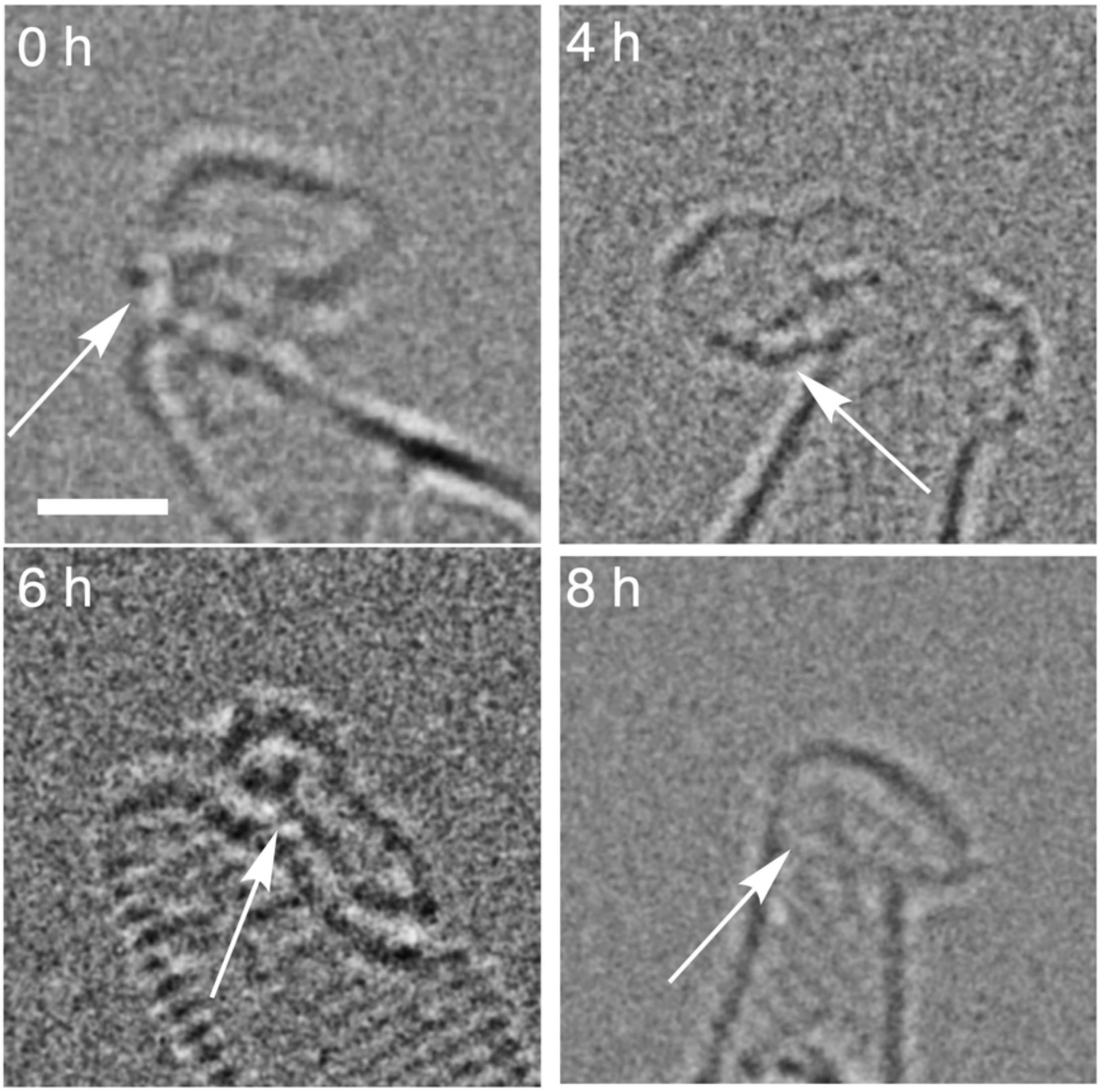

Supplementary Fig. 25. Representative TEM images of lower order PNCs of MOF-5 at 0.01 M of Zn^{2+} for different reaction time (methanol wash). PNCs were identified by their motions while the CNH remained static. Reaction time is shown for each image. Scale bar is 1 nm.

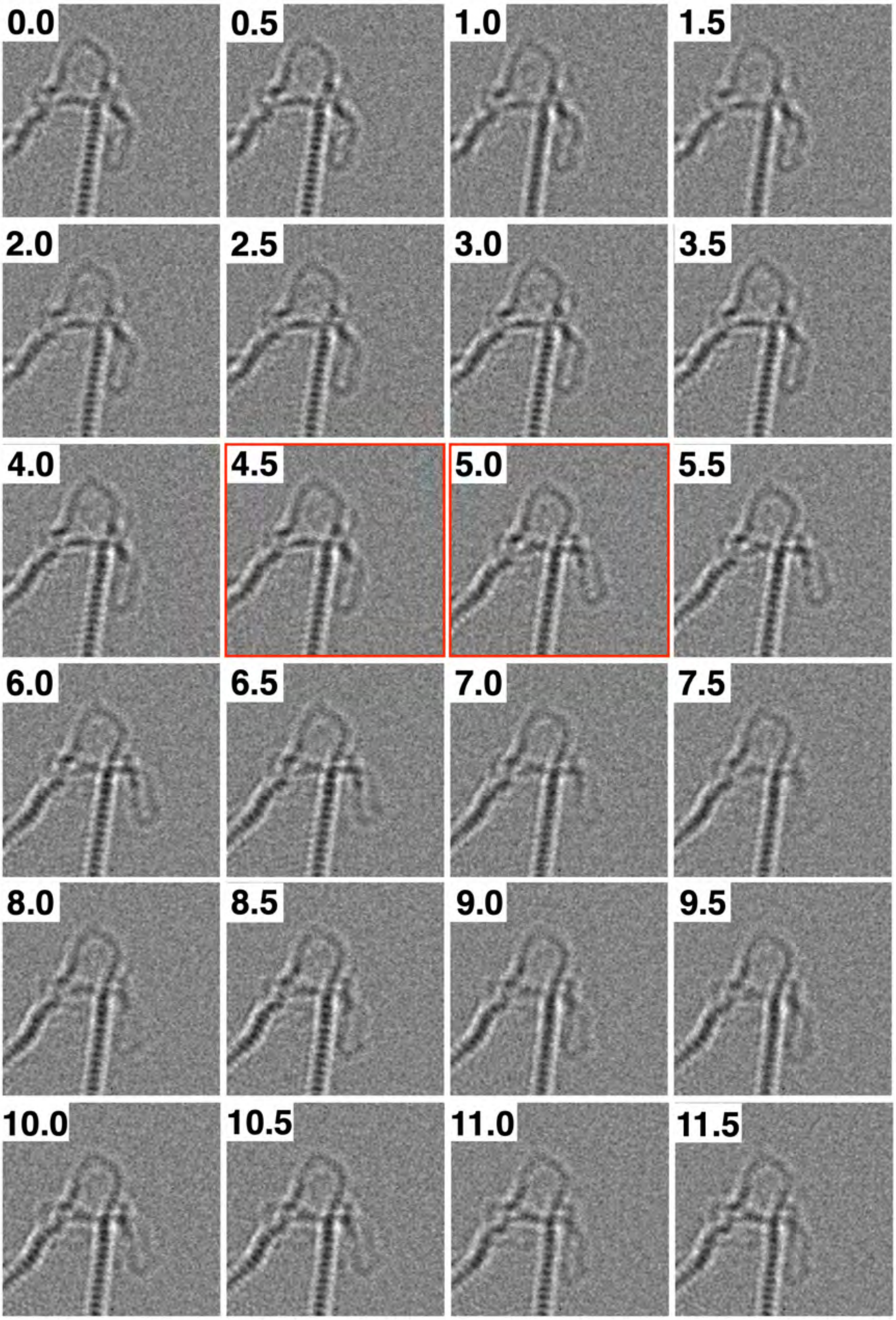
0.0
0.5
1.0
1.5
2.0
2.5
3.0
3.5
4.0
4.5
5.0
5.5
6.0
6.5
7.0
7.5
8.0
8.5
9.0
9.5
10.0
10.5
11.0
11.5

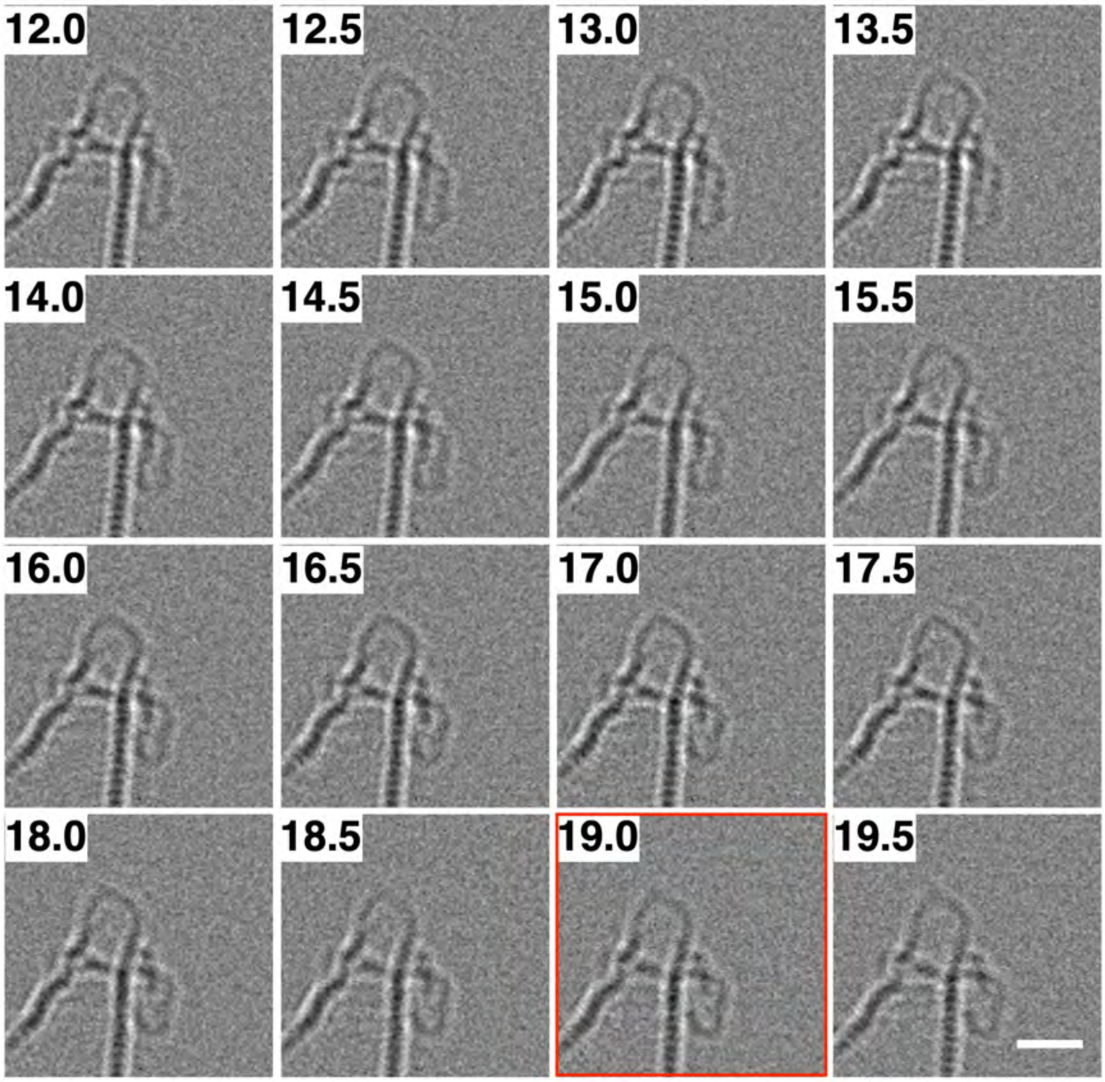

Supplementary Fig. 26. SMART-EM image sequences of a mobile square PNC of MOF-2 at 0.01 M of Zn^{2+} for 21 h (methanol wash) under an acceleration voltage of 80 kV and electron dose rate of 1.0×10^6 e^- nm^{-2} s^{-1}. The motion picture is shown in Supplementary Movie 1. The representative images used for structural analysis in Fig. 3d–f are highlighted in red squares. Numbers denote the time in second after starting video recording. Scale bar is 1 nm.

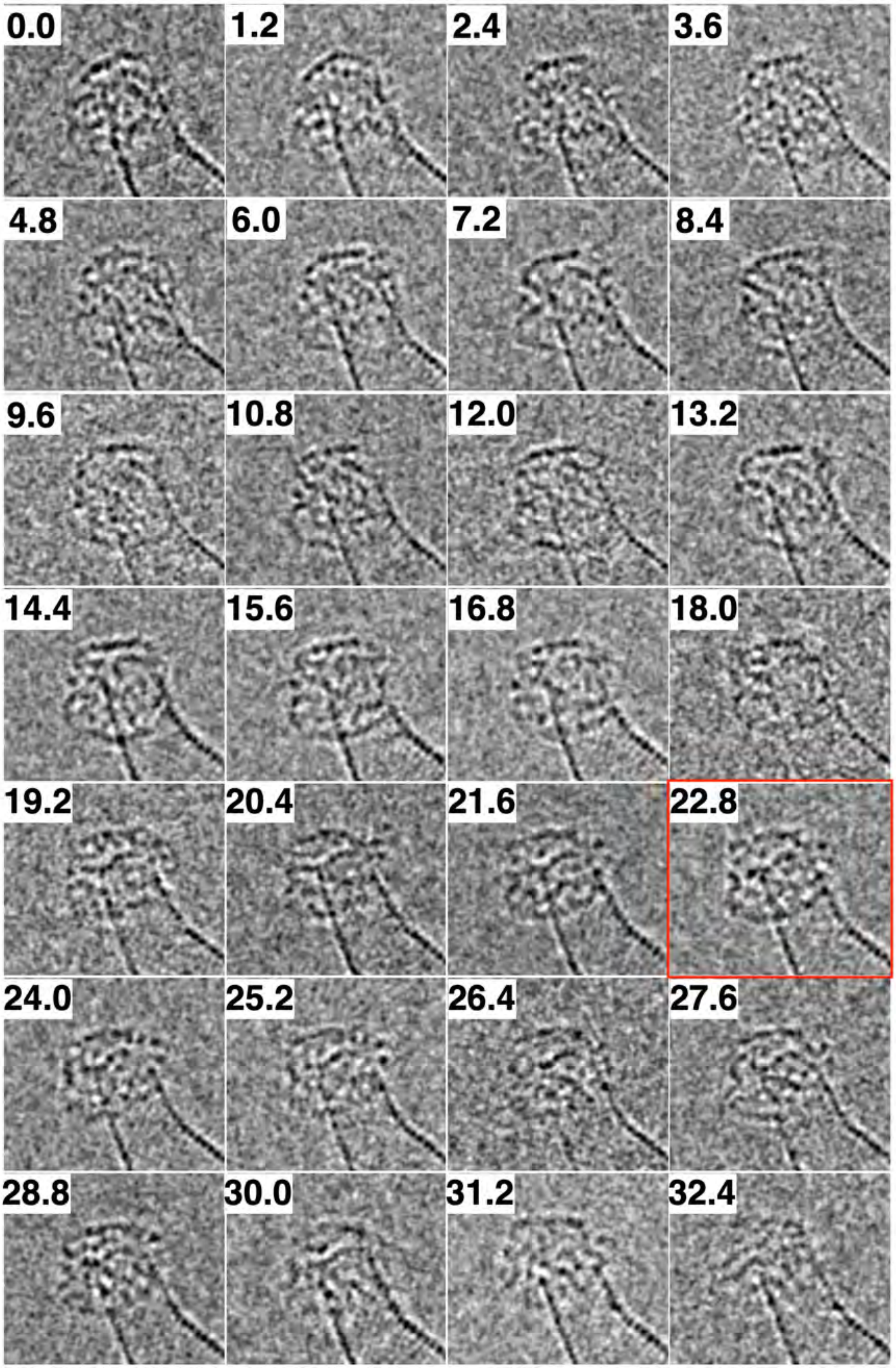
0.0
1.2
2.4
3.6
4.8
6.0
7.2
8.4
9.6
10.8
12.0
13.2
14.4
15.6
16.8
18.0
19.2
20.4
21.6
22.8
24.0
25.2
26.4
27.6
28.8
30.0
31.2
32.4

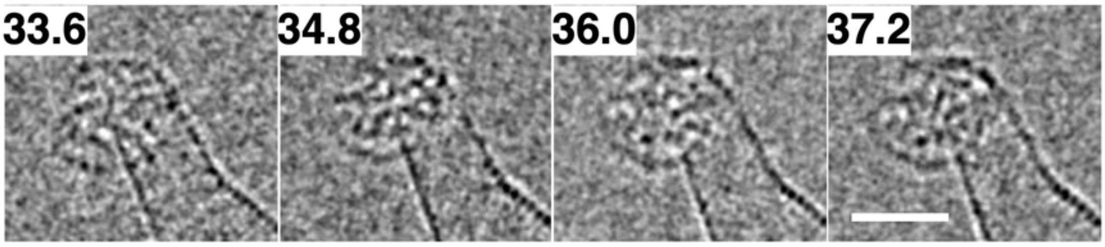

Supplementary Fig. 27. SMART-EM image sequences of a mobile cubic PNC of MOF-5 at 0.01 M of Zn^{2+} for 21 h (methanol wash) under an acceleration voltage of 120 kV and electron dose rate of 3.6×10^5 e^{-} nm^{-2} s^{-1}. The motion picture is shown in Supplementary Movie 2. The 22.8 s frame (highlighted by a red square) was subjected to structural analysis as shown in Fig. 5a–c. Numbers denote the time in second after starting video recording. Scale bar is 1 nm.

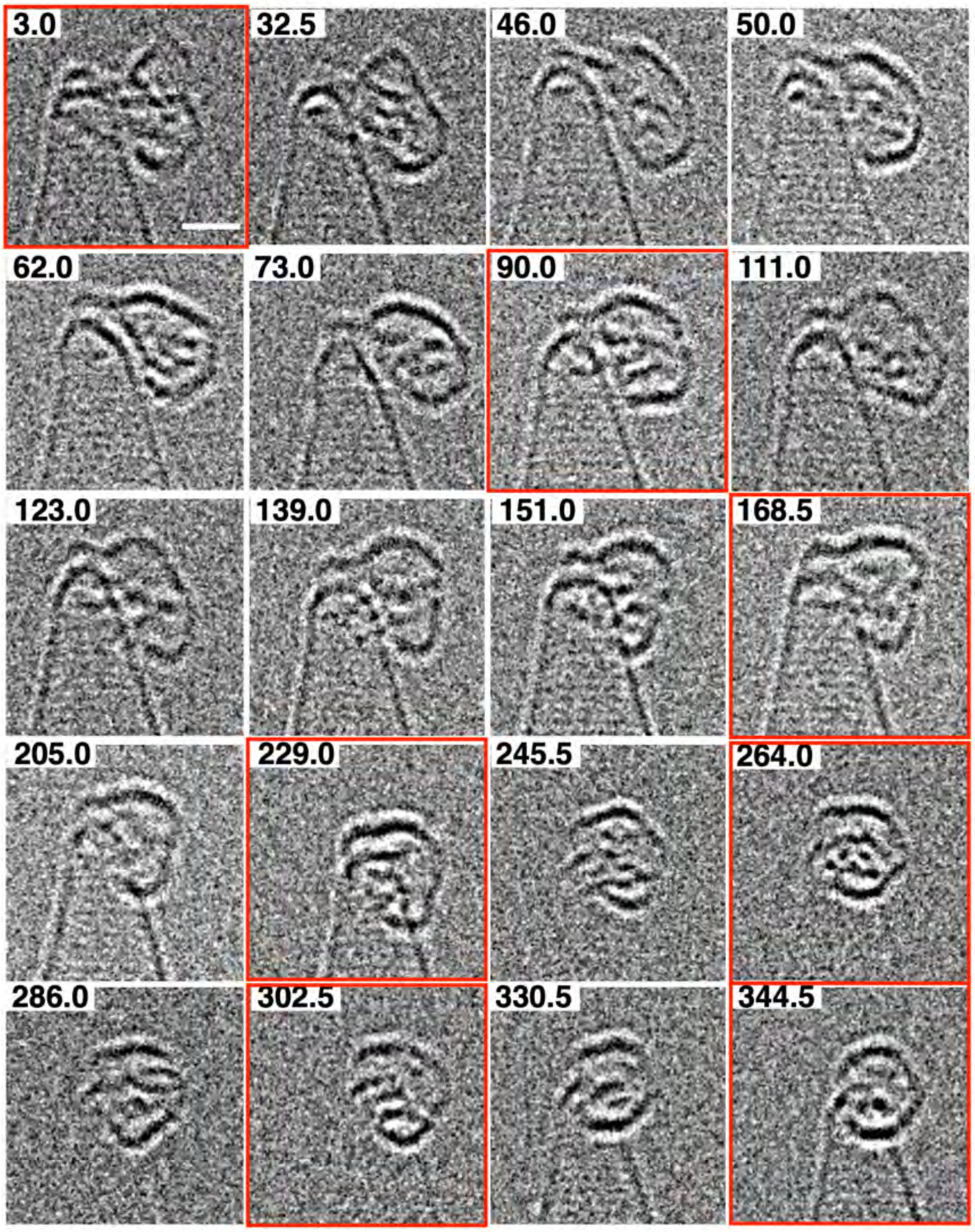

Supplementary Fig. 28. SMART-EM image sequences of structural reorganization of a PNC of I-MOF-5 at 0.01 M of Zn^{2+} for 21 h (dry transfer) under an acceleration voltage of 80 kV and electron dose rate of 1.5×10^6 e^- nm^{-2} s^{-1}. The motion picture is shown in Supplementary Movie 3. Numbers denote the time in second after starting video recording. The frames shown in Fig. 5d are highlighted in red squares. Scale bar is 1 nm.

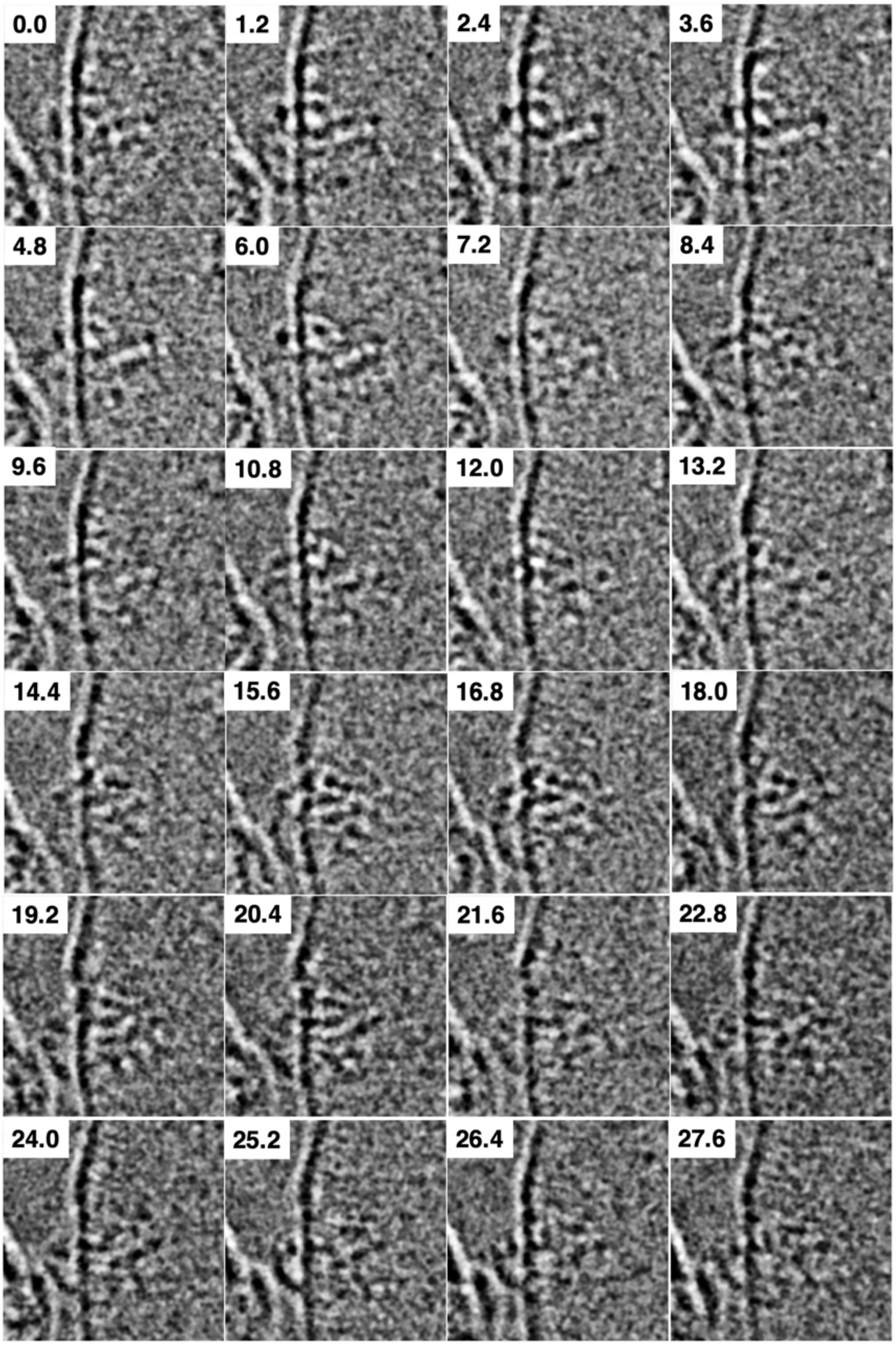
0.0
1.2
2.4
3.6
4.8
6.0
7.2
8.4
9.6
10.8
12.0
13.2
14.4
15.6
16.8
18.0
19.2
20.4
21.6
22.8
24.0
25.2
26.4
27.6

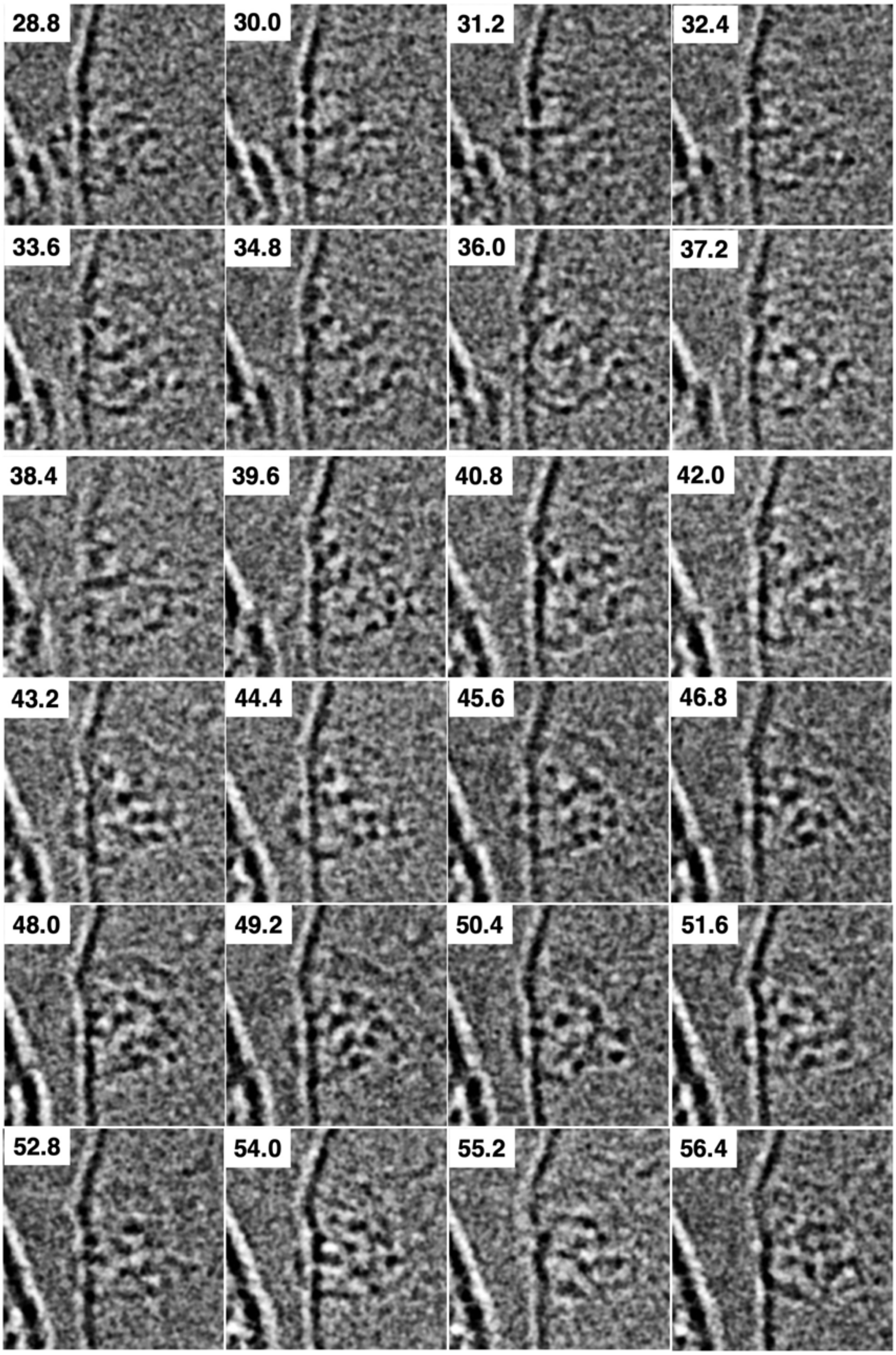
28.8
30.0
31.2
32.4
33.6
34.8
36.0
37.2
38.4
39.6
40.8
42.0
43.2
44.4
45.6
46.8
48.0
49.2
50.4
51.6
52.8
54.0
55.2
56.4

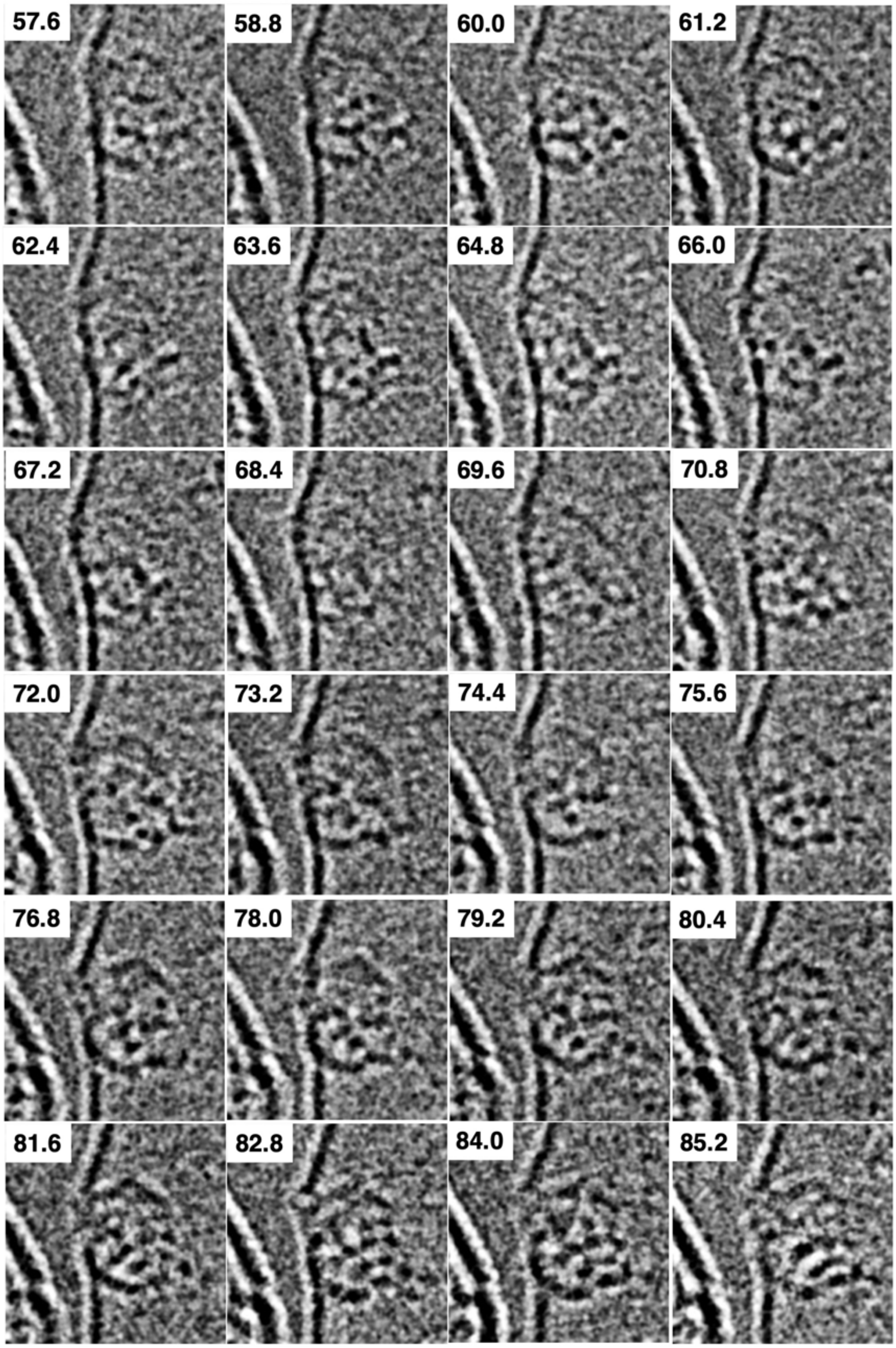
57.6
58.8
60.0
61.2
62.4
63.6
64.8
66.0
67.2
68.4
69.6
70.8
72.0
73.2
74.4
75.6
76.8
78.0
79.2
80.4
81.6
82.8
84.0
85.2

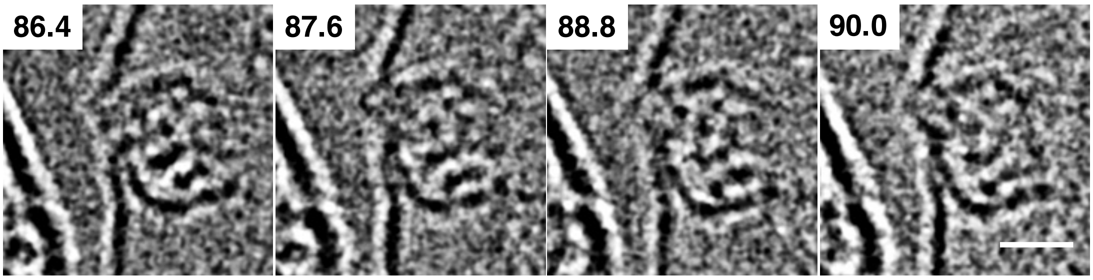

Supplementary Fig. 29. SMART-EM image sequences of structural reorganization of a PNC of I-MOF-5 at 0.01 M of Zn^{2+} for 21 h (methanol wash) under an acceleration voltage of 120 kV and electron dose rate of 5.4×10^5 e^{-} nm^{-2} s^{-1}. The motion picture is shown in Supplementary Movie 4. Numbers denote the time in second after starting video recording. Scale bar is 1 nm.

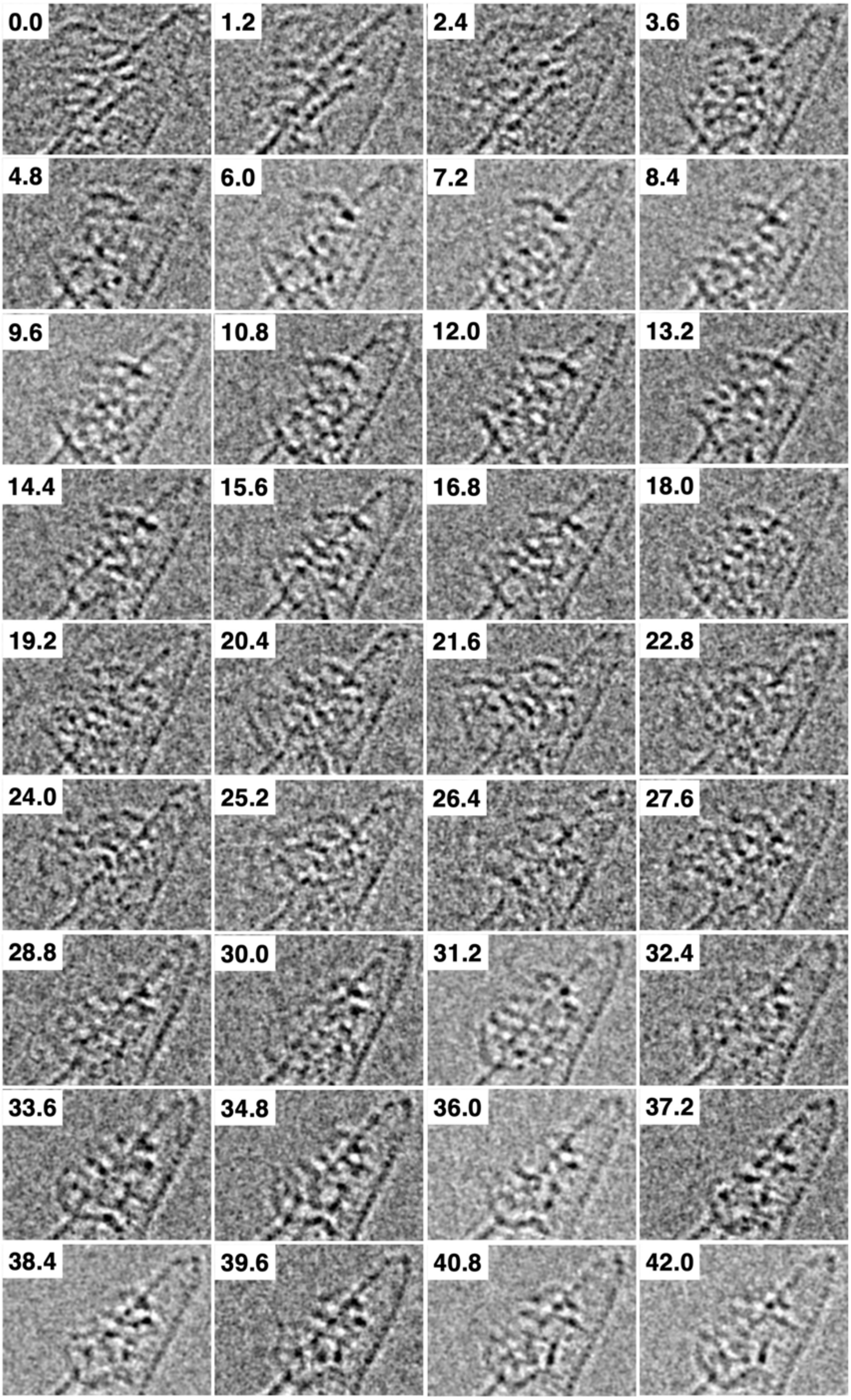
0.0
1.2
2.4
3.6
4.8
6.0
7.2
8.4
9.6
10.8
12.0
13.2
14.4
15.6
16.8
18.0
19.2
20.4
21.6
22.8
24.0
25.2
26.4
27.6
28.8
30.0
31.2
32.4
33.6
34.8
36.0
37.2
38.4
39.6
40.8
42.0

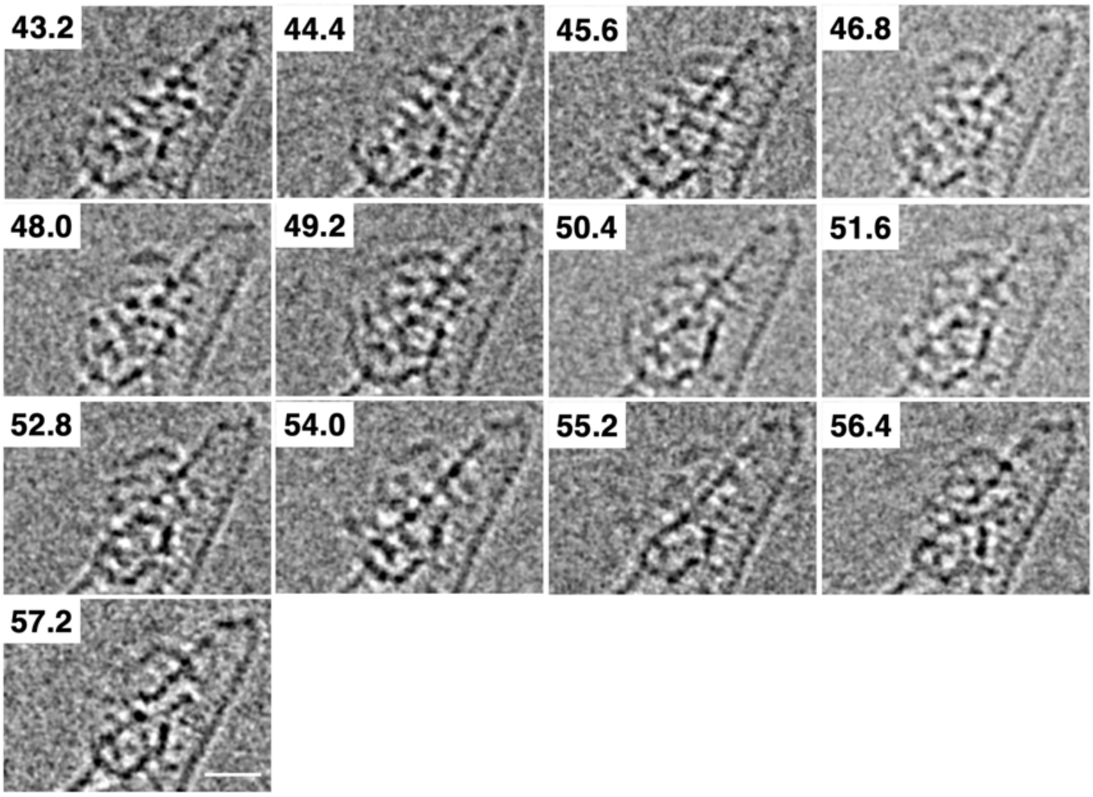

Supplementary Fig. 30. SMART-EM image sequences structural reorganization of a PNC of I-MOF-5 at 0.01 M of Zn^{2+} for 21 h (methanol wash) under an acceleration voltage of 120 kV and electron dose rate of 5.4×10^5 e^- nm^{-2} s^{-1}. The motion picture is shown in Supplementary Movie 5. Numbers denote the time in second after starting video recording. Scale bar is 1 nm.

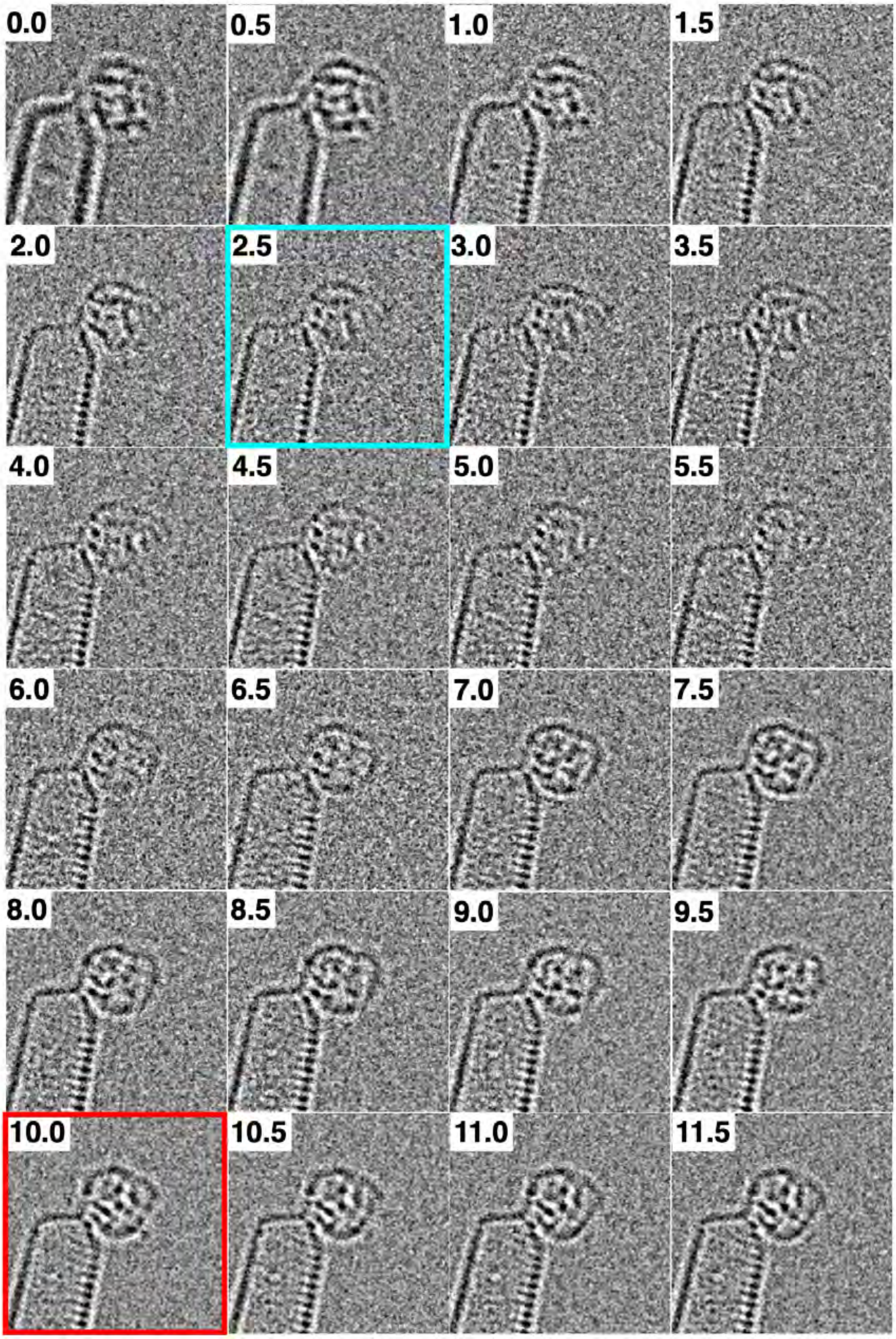
0.0
0.5
1.0
1.5
2.0
2.5
3.0
3.5
4.0
4.5
5.0
5.5
6.0
6.5
7.0
7.5
8.0
8.5
9.0
9.5
10.0
10.5
11.0
11.5

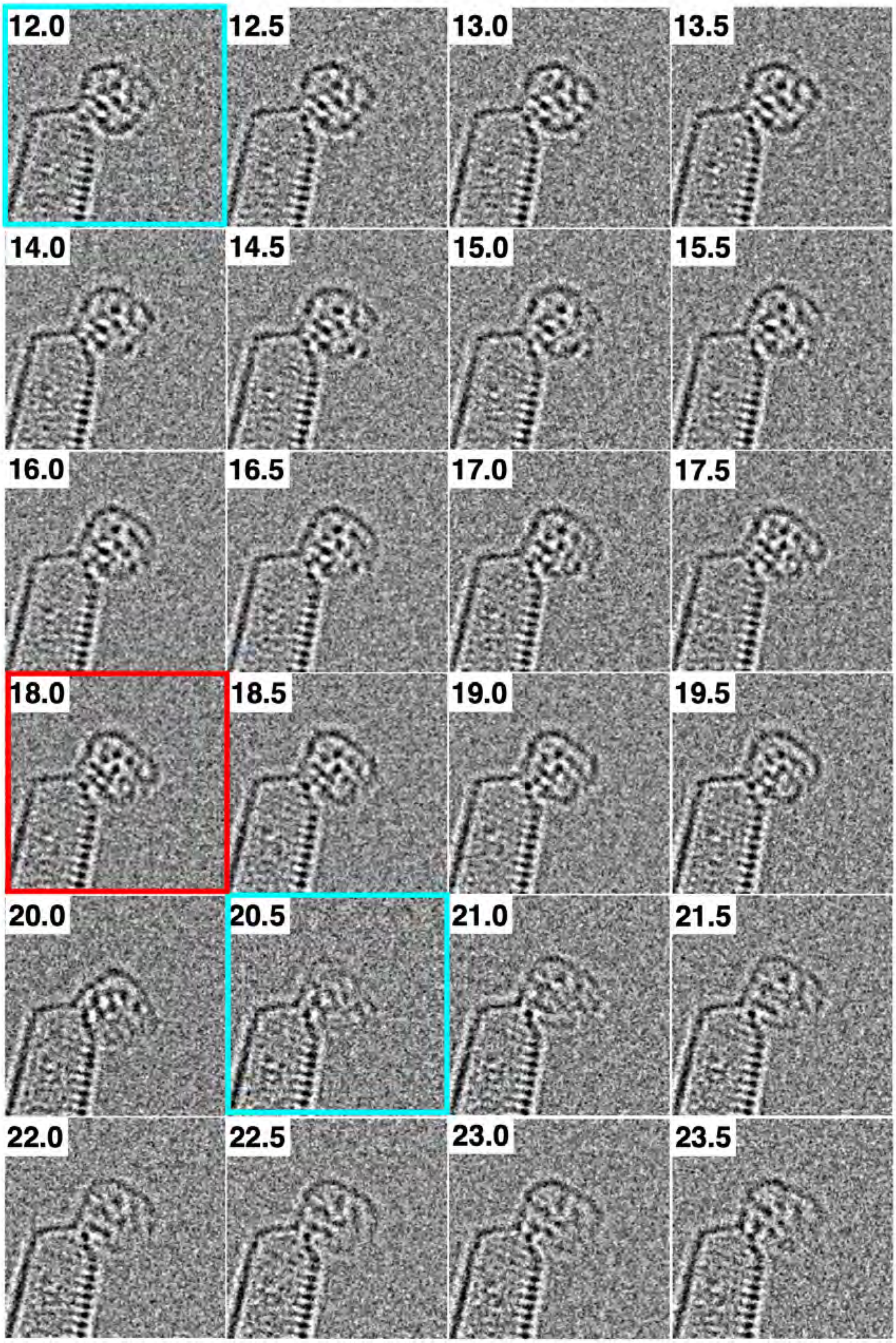

12.0
12.5
13.0
13.5
14.0
14.5
15.0
15.5
16.0
16.5
17.0
17.5
18.0
18.5
19.0
19.5
20.0
20.5
21.0
21.5
22.0
22.5
23.0
23.5

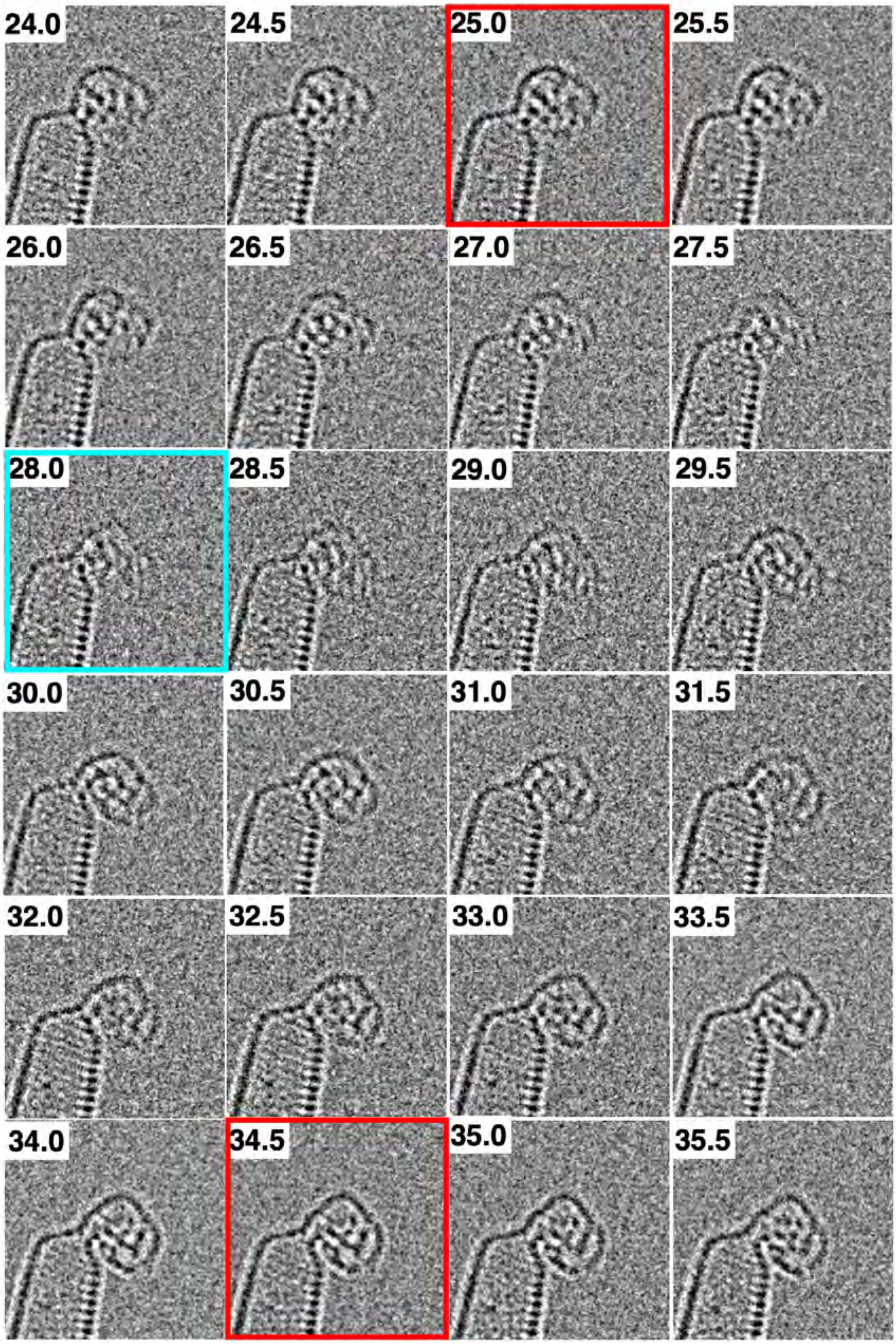
24.0
24.5
25.0
25.5
26.0
26.5
27.0
27.5
28.0
28.5
29.0
29.5
30.0
30.5
31.0
31.5
32.0
32.5
33.0
33.5
34.0
34.5
35.0
35.5

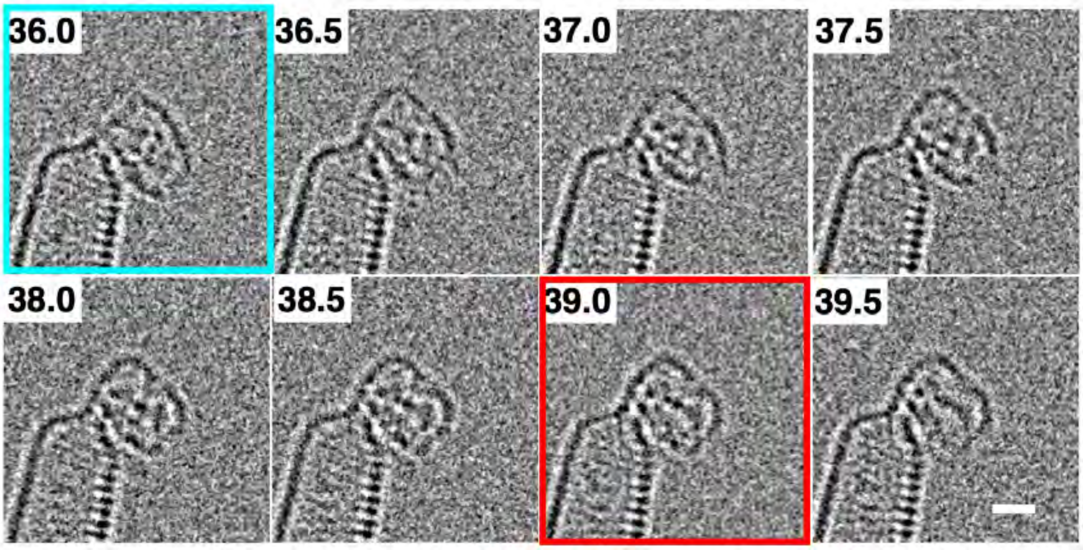

Supplementary Fig. 31. SMART-EM image sequences of a rotating PNC of I-MOF-5 at 0.01 M of Zn^{2+} for 21 h (methanol wash) under an acceleration voltage of 80 kV and electron dose rate of 1.1×10^6 e^- nm^{-2} s^{-1}. The motion picture is shown in Supplementary Movie 6. Five high contrast frames shown in Fig. 6a are highlighted by red squares, and five low contrast frames by light blue squares. Numbers denote the time in second after starting video recording. Scale bar is 1 nm.

Supplementary References

1. Williams, D. B. & Carter, C. B. *Transmission Electron Microscopy: A Textbook for Materials Science*, 2nd Ed. (Springer US, New York, 2009).
2. Clausen, H. F., Poulsen, R. D., Bond, A. D., Chevallier, M.-A. S. & Iversen, B. B. Solvothermal synthesis of new metal organic framework structures in the zinc-terephthalic acid-dimethyl formamide system. *J. Solid State Chem.* **178**, 3342–3351 (2005).
3. Tranchemontagne, D. J., Hunt, J. R. & Yaghi, O. M. Room temperature synthesis of metal-organic frameworks: MOF-5, MOF-74, MOF-177, MOF-199, and IRMOF-0. *Tetrahedron* **64**, 8553–8557 (2008).
4. Eddaoudi, M. et al. Systematic Design of Pore Size and Functionality in Isoreticular MOFs and Their Application in Methane Storage. *Science* **295**, 469-472 (2002).
5. Meek, S. T., Perry, J. J., Teich–McGoldrick, S. L., Greathouse, J. A. & Allendorf, M. D. Complete Series of Monohalogenated Isoreticular Metal-Organic Frameworks: Synthesis and the Importance of Activation Method. *Cryst. Growth Des.* **11**, 4309–4312 (2011).
