# Supplementary material for: Atomistic structures and dynamics of prenucleation clusters in MOF-2 and MOF-5 syntheses
Source: Nat Commun. 2019 Aug 23;10:3608. doi: 10.1038/s41467-019-11564-4 (PMC6707309; doi:10.1038/s41467-019-11564-4)
Supplement: Supplementary file 2 — Description of Additional Supplementary Files [file 41467_2019_11564_MOESM2_ESM.pdf]

Description of Additional Supplementary Files

File Name: Supplementary Movie 1
Description: **Motion of a PNC of MOF-2-DMF (80 kV).** The original video of the frames in Fig. 3d–f. Whole frames are shown in Supplementary Fig. 26. The experimental conditions are E = 80 kV, EDR = 10.3 × 10^5 e^- nm^{-2} s^{-1} and 0.5 s for exposure time for each frame. The video represents the motion five times faster than the actual time because the 0.5 s interval has been reduced to 0.1 s in the video.

File Name: Supplementary Movie 2
Description: **Motion of a PNC of MOF-5 (120 kV).** The original video of the frame in Fig. 5a. Whole frames are shown in Supplementary Fig. 27. The experimental conditions are E = 120 kV, EDR = 3.5 × 10^5 e^- nm^{-2} s^{-1} and 0.4 s for exposure time for each frame. The video represents the motion 12 times faster than the actual time because the 1.2 s interval has been reduced to 0.1 s in the video.

File Name: Supplementary Movie 3
Description: **Structural reorganization process of a PNC of I-MOF-5 (80 kV).** The original video of the frames in Fig. 5d. Representative frames are shown in Supplementary Fig. 28. The experimental conditions are E = 80 kV, EDR = 15.3 × 10^5 e^- nm^{-2} s^{-1} and 0.5 s for exposure time for each frame. The video represents the motion 50 times faster than the actual time because the 0.5 s interval has been reduced to 0.01 s in the video.

File Name: Supplementary Movie 4
Description: **Structural reorganization process of a PNC of I-MOF-5 (120 kV).** Whole frames are shown in Supplementary Fig. 29. The experimental conditions are E =120 kV, EDR = 5.4 × 10^5 e^- nm^{-2} s^{-1} and 0.4 s for exposure time for each frame. The video represents the motion 12 times faster than the actual time because the 1.2 s interval has been reduced to 0.1 s in the video.

File Name: Supplementary Movie 5
Description: **Structural reorganization process of a PNC of I-MOF-5 (120 kV).** Whole frames are shown in Supplementary Fig. 30. The experimental conditions are E =120 kV, EDR = 5.4 × 10^5 e^- nm^{-2} s^{-1} and 0.4 s for exposure time for each frame. The video represents the motion 12 times faster than the actual time because the 1.2 s interval has been reduced to 0.1 s in the video.

File Name: Supplementary Movie 6
Description: **Motion of a PNC of I-MOF-5 (80 kV).** The original video of the frames in Fig. 6a. Whole frames are shown in Supplementary Fig. 31. The experimental conditions are E = 80 kV, EDR = 10.9 × 10^5 e^- nm^{-2} s^{-1} and 0.5 s for exposure time for each frame. The video represents the motion five times faster than the actual time because the 0.5 s interval has been reduced to 0.1 s in the video.
