# Supplementary material for: Atomistic structures and dynamics of prenucleation clusters in MOF-2 and MOF-5 syntheses
Source: Nat Commun. 2019 Aug 23;10:3608. doi: 10.1038/s41467-019-11564-4 (PMC6707309; doi:10.1038/s41467-019-11564-4)
Supplement: Supplementary file 9 — Peer Review File [file 41467_2019_11564_MOESM9_ESM.pdf]

Reviewers' comments:

Reviewer #1 (Remarks to the Author):

The paper “Atomistic structures and dynamics of prenucleation clusters in MOF-2 and -5 syntheses” by Junfei Xing et al. pertains the visualization and identification of clusters present in the synthesis solutions of MOFs via high resolution TEM. The images and the level of detail in the structural determination of these clusters that can be obtained from them are absolutely spectacular! As one can see below, I have an issue with claiming that these clusters in the high vacuum of the TEM are the same as those in the heated synthesis solution. Yet, it is extremely hard to obtain this level of structural insight in prenucleation clusters with any other technique, and as such, pertaining to the importance of crystallization processes, this is quite a holy grail. If the authors can really claim that the differences in clusters originating from the different synthesis conditions leading to different MOFs is statistically meaningful, than I find these results quite meaningful and exciting.

In any case, though, some key experiments and data are missing.
Page 2, line 16-18, the authors claim: “ Here, we report that the MOF-2/-5 bifurcation occurs already at the prenucleation stage before crystal nucleation and growth, as suggested by the formation of square PNCs in the MOF-2 synthesis, and of cubic PNCs in the MOF-5 synthesis. “ This is the central claim of the paper. However, the PNC’s are only harvested after 21 hours of synthesis when the MOF crystal yield is already 70-80%, and reactants are significantly depleted. So how can one state that “the prenucleation stage before crystal nucleation and growth” is targeted? The DLS results show that even at synthesis time 0, particles of 1 nm are present. (On a side note: This time 0 is moreover ill-defined. I presume this is the time that the synthesis solution reaches it’s temperature. Can this be specified, as well as the heating rate?) In any case, for the authors to claim that these are prenucleation centres, they should also be identified before crystallization takes place, which becomes visible according to the authors at 30 minutes. So time 0 should be a good time to harvest the PNC’s. (Note: Time 0 could even be better defined by mixing two preheated solution that contain either the inorganic Zn^{2+} ions or the organic ligand). In any case, similar experiments need to be conducted at early times.

Another question that remains is how representative are these identified PNC’s of the the PNC’s present in the solution. I mean the fished out PNC’s are washed with a solvent and dried in vacuum, and then measured in the TEM in ultrahigh vacuum. Did the clusters not undergo changes in those processes? What are the arguments to state that they are the same clusters as those in the synthesis solution at elevated temperature?

A third issue related to “representativeness” is statistical representativeness. How representative are the shown TEM-images with PNC’s. Meaning how many CNT’s with PNC were imaged for a particular synthesis? How many of these PNC’s showed the same structure (e.g. cubic), how many showed other structures? How many showed unidentifiable structures? This information needs to be added. The information that is now provided is also confusing. In figure 1g, it shows that in MOF-5 synthesis, cubic PNC are hardly found (only as a minority in more concentrated syntheses), yet the text suggests that cubic PNC’s are all that is found in these syntheses.

At this moment the paper reads as a collection of individual observation, rather than a consistent story, I found it quite hard to read. Simply reading the supporting information was far more informative. This should be improved.

Some small comments:
• In the Materials and Methods, there is no mention of whether the synthesis is done in an open versus closed vessel, or the material of the vessel (Teflon, glass?) and the degree of filling of the vessel.
• The yield table in the supplementary info is impossible to understand. What does the line DMF

removed refer to (which synthesis)? And after which step is the yield determined in each case? And how can one determine yield while solvent molecules are not removed? This isn't meaningful. Determining the yield is only sensible for each activated sample so that you do not end up with values above 100%.
• The synthesis conditions for the MOFs without the carbon nanotubes present are not given.
• For many of the data (graphs, images, figures) it is not indicated to which experimental conditions they exactly pertain, specifically the concentration of the reactants is not mentioned while for each different MOF material a series of syntheses with different concentrations are performed

Reviewer #2 (Remarks to the Author):

This manuscript describes some very interesting work relating to structure elucidation of pre-nucleating units in the synthesis of two MOFs. The work, particularly techniques involved, certainly merit publication in this journal and the manuscript should be accepted after minor/ major revisions that mainly affect the clarity of the manuscript.
Suggestions listed below:
(i)Use the term "node" instead of "knot" throughout manuscript as typically used in the MOF literature
(ii) The Introduction seems to run into the "Results and discussion" section - page 3 and 4 of manuscript appear to mainly be introduction and not results. Some revision/ rewriting of these early pages needs doing.
(iii)Is there DLS data for the synthesis of MOF2 - if so it should be included in SI - if not why not?
(iv) CNH needs to be defined early on in the text, I assume it stands for carbon nanohorn
(v)More detailed crystal structure diagrams for MOF-2 and MOF-5 need to be given - probably in SI
(v)in Fig 1b - should there be arrows between lower order 120C PNC and the semicube
(vi) in fig 1c - should include + or - L (ligands) in each equilibria. Also not clear where the charges have come from in the structural figures next to each knot in the bottom of Fig 1c - why are some O atoms coloured pink and some charges for O are +2 and the Zn are -1 - clarify meaning here.
(vii) Were TEM images and simulations able to be determined for the lower order species? Images are given in the SI but were there attempts to simulate these.

Reviewer #3 (Remarks to the Author):

This manuscript reports the use of an electron microscopy technique, SMART-EM, to measure the structure of intermediate stages during liquid phase crystallization of MOF structures. Correlation with light scattering was used to identify the presence of 1nm clusters. The structure of the clusters was identified with SMART-EM and used to infer mechanisms of MOF crystallization.
The SMART-EM technique is well established at this point, as the authors have imaged a number of other organic molecules tethered to carbon nanohorns (CNH) or within carbon nanotubes, and have discussed both the unexpected resilience of many molecules to electron beam damage and the motion of the molecules as a series of jumps between relatively static configurations. This manuscript applies the SMART technique to attempt to clarify the liquid-phase MOF formation mechanism. The images presented are interesting and a large amount of data is shown. However I have questions about the interpretation and I believe that the data presented does not unambiguously support the discussion of the formation mechanism.

(1) In the experiment, the MOF crystallization procedure takes place in the presence of CNHs that are functionalized with BDH that forms sites for PNCs. The authors mention that dynamic light scattering is definitive in showing only one type of PNC in the solution. But the authors need to justify that the PNCs that they measure with SMART, the ones that attached to the CNHs, are the

same structure as the ones in solution measured by light scattering. This is not obvious to me as a non-expert in MOF chemistry, since these PNCs have formed in a different environment (in proximity to the “substrate” CNH, with a tethered BDH molecule).

(1b) A related question is whether the PNC changed their structure during the drying process, compared to the structure measured by light scattering that is still in the liquid phase.

(2) “..washing of the CNH specimens with methanol suppressed … motions of the CNHs and the PNCs, and allowed us to reproducibly obtain atomic-resolution TEM images without apparent change in the appearance of PNCs…”
The mechanism of this methanol-induced stability needs to be explained better, since the highest quality data is from the immobilized structures making an assumption that they are the same as in the initial liquid sample.

(3) In previous publications, the authors have discussed the stability under the beam of individual molecules in SMART imaging. Here, they show that changes do occur under the beam, such as the change from lower to higher order PNC in Figure 3. Given that changes can occur, the authors need to justify that some rapid change has not occurred at say the onset of imaging that could affect the interpretation.

(4) The iodine experiment is a good strategy because it allows more detailed analysis of the images. However, a superficial comparison of experiment and model in fig 4g and h still shows somewhat poor agreement in the positions of the dark spots. For example atom 8 does not show in the experimental image, and the 1A microscope resolution would not account for a situation where a dark spot is missing. It is not clear what analysis was carried out for other cube PNCs such as those shown in Supplementary Figures 17 and 19. Since the structure of the cube is a central finding of the manuscript, the argument would be more convincing if several PNCs could be analyzed and confidence levels presented to show that one structure fits all data best; if I understand the text correctly, only one cube structure was fully analyzed and this analysis was only based on a handful of video frames.

(5) The image matching procedure, described throughout the manuscript and the Supplementary Information, appears to be qualitative in relying on a visual estimate of the similarity of simulation and image. Only high-contrast movie frames are analyzed. This is based on a reasonable argument about motion, but it is hard to imagine zero motion over 0.4 or 0.5sec in even the high contrast frames. The results would be more convincing if all data were to be included in the analysis with a process that gives less weight to images with less information content, as is common in analysis of cryoelectron microscopy data.

(A very minor point: In fig 1d and Supplementary Figure 12 I was not sure what the large ovals show.)

Point-by-point response to the reviewers
(The reviewers' comments are highlighted in blue.)

Reviewer #1 (Remarks to the Author):
The paper "Atomistic structures and dynamics of prenucleation clusters in MOF-2 and -5 syntheses" by Junfei Xing et al. pertains the visualization and identification of clusters present in the synthesis solutions of MOFs via high resolution TEM. The images and the level of detail in the structural determination of these clusters that can be obtained from them are absolutely spectacular! As one can see below, I have an issue with claiming that these clusters in the high vacuum of the TEM are the same as those in the heated synthesis solution. Yet, it is extremely hard to obtain this level of structural insight in prenucleation clusters with any other technique, and as such, pertaining to the importance of crystallization processes, this is quite a holy grail. If the authors can really claim that the differences in clusters originating from the different synthesis conditions leading to different MOFs is statistically meaningful, than I find these results quite meaningful and exciting.

The reviewer raised chemical and statistical issues, and I will answer them below. What we have discovered in the past months is the complexity of the reaction itself previously not well recognized by the MOF community, as described below.

In any case, though, some key experiments and data are missing. Page 2, line 16-18, the authors claim: " Here, we report that the MOF-2/-5 bifurcation occurs already at the prenucleation stage before crystal nucleation and growth, as suggested by the formation of square PNCs in the MOF-2 synthesis, and of cubic PNCs in the MOF-5 synthesis. " This is the central claim of the paper. However, the PNC's are only harvested after 21 hours of synthesis when the MOF crystal yield is already 70-80%, and reactants are significantly depleted. So how can one state that "the prenucleation stage before crystal nucleation and growth" is targeted? The DLS results show that even at synthesis time 0, particles of 1 nm are present. (On a side note: This time 0 is moreover ill-defined. I presume this is the time that the synthesis solution reaches it's temperature. Can this be specified, as well as the heating rate?) In any case, for the authors to claim that these are prenucleation centres, they should also be identified before crystallization takes place, which becomes visible according to the authors at 30 minutes. So time 0 should be a good time to harvest the PNC's. (Note: Time 0 could even be better defined by mixing two preheated solution that contain either the inorganic Zn2+ ions or the organic ligand). In any case, similar experiments need to be conducted at early times.

This is an extremely important suggestion. The reaction initially gave no MOF-5 at all, as reported in *Cryst. Growth Des.* **2015**, *15*, 4781−4786.

Instead, a large portion of Zn and BDC precipitated (we call this as X), and X had a composition of Zn:BDC = 1:1 (the same as MOF-2). After 4 hours, X underwent change to a mixture of MOF-5 nanocrystallites and amorphous material as studied by PXRD. Further conversion to MOF-5 cubic polycrystals via Ostwald ripening requires additional 20-40 hours, as reported in *CrystEngComm*, **2014**, *16*, 1064. Thus, we focused on the 21-hour time where PNCs and MOF-5 crystals are equilibrating with each other during the Ostwald ripening without the presence of X.

In the past months, we carefully examined the time course (0–21 h) of the change of the structure of PNCs captured on BDC-CNH, and of the pH of the reaction mixture after water quenching. This data is shown now in Fig. 1h. We learn here that the pH steadily increased and X started to convert to MOF-5 when the solution became basic. Likewise, higher order PNCs (cube and cube-like) increased steadily, as pH increased.

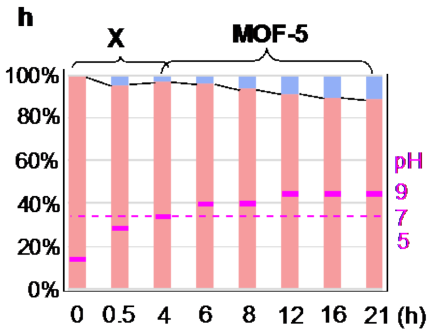

Fig. 1h, The ratio between LO (pink) and HO (blue) clusters in the formation of **X** and MOF-5 at 120 °C. The data in purple indicate the pH of reaction mixture after dilution with water. The total number of the PNCs examined in the eight experiments shown are the following from the left to the right: 47, 27, 132, 133, 67, 58, 69, 36.

This data in Fig. 1h make the chemistry much clearer for us. Initially, the solution was acidic ($Zn(NO_3)_2 + 2 \bullet RCOOH = Zn(RCOO)_2 + 2 \bullet HNO_3$), and only X formed (PXRD). As DMF decomposes, O^{2-} gradually formed to change the lower order PNCs (Zn:BDC = 1:1) into the higher order PNCs (O^{2-} incorporated). Once the solution became basic, the phase change from X to MOF-5 took place and consumed much of O^{2-}. After 8 hours, Ostwald ripening of MOF-5 crystals started, as the amount of higher order PNCs increased.

In the 0-min experiment that the reviewer suggested, we found PNCs essentially the same as those found in the MOF-2 synthesis. This is not unexpected since the solution was acidic and the only difference was the temperature (95 °C vs. 120 °C). We saw square PNCs as shown below (and included in SI, Supplementary Fig. 25).

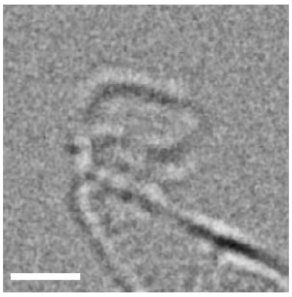
Supplementary Figure 25. A square cluster captured on BDC-CNH at 0 min. Scale bar: 1 nm.

Another question that remains is how representative are these identified PNC's of the the PNC's present in the solution. I mean the fished out PNC's are washed with a solvent and dried in vacuum, and then measured in the TEM in ultrahigh vacuum. Did the clusters not undergo changes in those processes? What are the arguments to state that they are the same clusters as those in the synthesis solution at elevated temperature?

What we want to report are the following: Our first finding is on the cluster size to be similar to that in solution, the second is that the PNCs fished out from the MOF-2 synthesis are different from those from the MOF-5 synthesis, and the third is that the square and the cubic structures of the isolated PNCs are closely related to those in the MOF-2 and -5 crystal lattice.

Some details are given. First, our terephthalic acid "fishhook" is a part structure of MOF crystals, and hence should be able to effectively capture PNCs in solution. Second, we expect that the chemical affinity of our terephthalic acid "fishhook" to PNCs should be roughly equal for various PNCs in MOF-2 and -5 syntheses, and hence that the ratios of clusters reflect those in solution reasonably well. Third, the similarity in size (1 nm) between the DLS data and the clusters captured from solution suggests that we captured right kind of species. Fourth, we believe that the salient features of the EM-observed structures reflect those in solution, because the captured clusters were removed from the reaction mixture by quick filtration and drying in vacuo (dry transfer). Specimens obtained by dry transfer and by MeOH wash shared the same characteristics, and hence we consider that the information obtained by MeOH-wash samples also reflects the structural features of PNCs in the reaction.

That being said, the structures that we saw on EM cannot be exactly the same as those in solution including ligands on metals and conformation of the BDC bridges. However, we consider that the observed square and the cube clusters are reasonable as PNCs to MOF-2 and -5, respectively.

A third issue related to "representativeness" is statistical representativeness. How representative are the shown TEM-images with PNC's. Meaning how many CNT's with PNC were imaged for a particular synthesis? How many of these PNC's showed the same structure (e.g. cubic), how many showed other structures? How many showed unidentifiable structures? This information needs to be added. The information that is now provided is also confusing. In figure 1g, it shows that in MOF-5 synthesis, cubic PNC are hardly found (only as a minority in more concentrated syntheses), yet the text suggests that cubic PNC's are all that is found in these syntheses.

We wish to remind the reviewer that we showed an answer to the question "How many CNT's with PNC were imaged for a particular synthesis?" already in the original Supplementary Information (new Supplementary Fig. 17 and Supplementary Table 3).

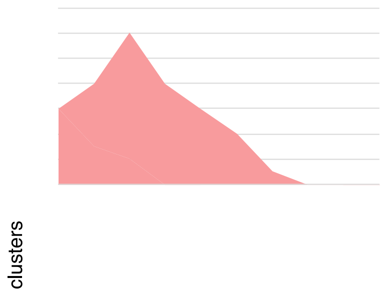

Our “fishing strategy” somewhat resembles “Census of Marine Life Project”, relying on the statistics. As has been shown in new Fig. 1h, i caption and in the new Supplementary Fig. 17 and Supplementary Table 3, we examined 27–133 samples in each analysis of clusters, meaning 8–19% statistical error. We consider therefore that our conclusion is statistically reliable enough to be published in this first report of the kind.

At this moment the paper reads as a collection of individual observation, rather than a consistent story, I found it quite hard to read. Simply reading the supporting information was far more informative. This should be improved.

We apologize for the confusing introductory part and rewrote the early pages.

Some small comments: • In the Materials and Methods, there is no mention of whether the synthesis is done in an open versus closed vessel, or the material of the vessel (Teflon, glass?) and the degree of filling of the vessel.

We added these details of the experimental procedure in SI.

• The yield table in the supplementary info is impossible to understand. What does the line DMF removed refer to (which synthesis)? And after which step is the yield determined in each case? And how can one determine yield while solvent molecules are not removed? This isn’t meaningful. Determining the yield is only sensible for each activated sample so that you do not end up with values above 100%.

We were careless about this. We reexamined the experiments again and obtained yields after removal of DMF that conform to the literature data (*Tetrahedron*, **2008**, *64*, 8553).

• The synthesis conditions for the MOFs without the carbon nanotubes present are not given.

We inadvertently omitted this data, which is now added in the experimental section. The conversion of X to MOF-5 took place about one to two hours earlier with BDC-CNHs than without, suggesting that BDC-CNHs participates in heterogeneous nucleation of MOF-5 formation.

• For many of the data (graphs, images, figures) it is not indicated to which experimental conditions they exactly pertain, specifically the concentration of the reactants is not mentioned while for each different MOF material a series of syntheses with different concentrations are performed

We added this information in all the figure captions in the text and Supplementary information.

Reviewer #2 (Remarks to the Author):
This manuscript describes some very interesting work relating to structure elucidation of pre-nucleating units in the synthesis of two MOFs. The work, particularly techniques involved, certainly merit publication in this journal and the manuscript should be accepted after minor/ major revisions that mainly affect the clarity of the manuscript.

Suggestions listed below:
(i)Use the term "node" instead of "knot" throughout manuscript as typically used in the MOF literature

We replaced “knot” with “node” in revised manuscript.

(ii) The Introduction seems to run into the "Results and discussion" section - page 3 and 4 of manuscript appear to mainly be introduction and not results. Some revision/ rewriting of these early pages needs doing.

We are sorry for the confusion, and rewrote the early pages as suggested, including the formation of X before formation of MOF-5, which we omitted in the original version.

(iii)Is there DLS data for the synthesis of MOF2 - if so it should be included in SI - if not why not?

We inadvertently omitted the MOF-2 data set in the original submission, and have added it in SI (Supplementary Fig. 16). Like MOF-5, we only observed 1-nm-sized clusters in MOF-2 synthesis.

(iv) CNH needs to be defined early on in the text, I assume it stands for carbon nanohorn

Done.

(v)More detailed crystal structure diagrams for MOF-2 and MOF-5 need to be given - probably in SI

We added crystal structure models in SI (Supplementary Fig. 18).

(v)in Fig 1b - should there be arrows between lower order 120C PNC and the semicube

Arrows are added.

(vi) in fig 1c - should include + or - L (ligands) in each equilibria.

We added – RCOO– below the equilibria.

Also not clear where the charges have come from in the structural figures next to each knot in the bottom of Fig 1c - why are some O atoms coloured pink and some charges for O are +2 and the Zn are -1 - clarify meaning here.

We removed the figures in the bottom of the old Fig. 1c mechanism to avoid confusion. The pink-colored oxygen indicates that it comes from Zn-O.

(vii) Were TEM images and simulations able to be determined for the lower order species? Images are given in the SI but were there attempts to simulate these.

For linear object, details could be discerned because of thermal vibration. Square and cubes vibrated much less and hence could be analyzed for some details.

Reviewer #3 (Remarks to the Author):
This manuscript reports the use of an electron microscopy technique, SMART-EM, to measure the structure of intermediate stages during liquid phase crystallization of MOF structures. Correlation with light scattering was used to identify the presence of 1nm clusters. The structure of the clusters was identified with SMART-EM and used to infer mechanisms of MOF crystallization.
The SMART-EM technique is well established at this point, as the authors have imaged a number of other organic molecules tethered to carbon nanohorns (CNH) or within carbon nanotubes, and have discussed both the unexpected resilience of many molecules to electron beam damage and the motion of the molecules as a series of jumps between relatively static configurations. This manuscript applies the SMART technique to attempt to clarify the liquid-phase MOF formation mechanism. The images presented are interesting and a large amount of data is shown.

However I have questions about the interpretation and I believe that the data presented does not unambiguously support the discussion of the formation mechanism.

The issues raised here are both chemical and statistical, and I will answer them below.

(1) In the experiment, the MOF crystallization procedure takes place in the presence of CNHs that are functionalized with BDH that forms sites for PNCs. The authors mention that dynamic light scattering is definitive in showing only one type of PNC in the solution. But the authors need to justify that the PNCs that they measure with SMART, the ones that attached to the CNHs, are the same structure as the ones in solution measured by light scattering. This is not obvious to me as a non-expert in MOF chemistry, since these PNCs have formed in a different environment (in proximity to the “substrate” CNH, with a tethered BDH molecule).

Our “fishing strategy” somewhat resembles “Census of Marine Life Project”, whose validity relies on the sampling technique. We believe that we did by far the best among what has been done to identify PNCs in the MOF synthesis. First, our terephthalic acid “fishhook” to PNCs is a chemically powerful fishhook, because this acid is a part structure of MOF crystals, and hence should be able effectively capture PNCs in solution. Second, we expect that the chemical affinity of our terephthalic acid “fishhook” to PNCs should be roughly equal for various PNCs in MOF-2 and -5 syntheses, and hence that the ratios of clusters reflect those in solution. Third, the similarity in size (1 nm) between the DLS data and the clusters captured from solution suggests that we captured right kind of species. Finally, the square and cube PNCs that we captured are the type of PNCs that people expect for MOF-2 and MOF-5 crystal growth.

(1b) A related question is whether the PNC changed their structure during the drying process, compared to the structure measured by light scattering that is still in the liquid phase.

That being said, the structures that we saw cannot be exactly the same as those in solution, like dried fish vs. live fish. However, we believe that the salient features of the EM-observed structures like linear, square vs. cubic reflect those in solution,

because we quickly filtered the captured clusters off from the reaction mixture and immediately dried in vacuo for EM imaging (dry transfer method).

(2) “..washing of the CNH specimens with methanol suppressed ... motions of the CNHs and the PNCs, and allowed us to reproducibly obtain atomic-resolution TEM images without apparent change in the appearance of PNCs...” The mechanism of this methanol-induced stability needs to be explained better, since the highest quality data is from the immobilized structures making an assumption that they are the same as in the initial liquid sample.

In the dry transfer samples, PNCs sometimes formed a more stable conformation from a less stable one (cf. Fig. 3e). The methanol wash samples were seen to be conformationally more stable, possibly because methanol changed the coordination environment on the zinc atoms (e.g., ligand exchange from DMF to MeOH).

(3) In previous publications, the authors have discussed the stability under the beam of individual molecules in SMART imaging. Here, they show that changes do occur under the beam, such as the change from lower to higher order PNC in Figure 3. Given that changes can occur, the authors need to justify that some rapid change has not occurred at say the onset of imaging that could affect the interpretation.

The rapid conformational changes such as in Fig. 3e are exceptional (and hence highlighted in Fig. 3), and most of the PNCs did not change their morphology during observation. We consider that these exceptional cases occurred because of thermal vibration of CNH, rather than the cleavage of C-C bonds.

(4) The iodine experiment is a good strategy because it allows more detailed analysis of the images. However, a superficial comparison of experiment and model in fig 4g and h still shows somewhat poor agreement in the positions of the dark spots. For example atom 8 does not show in the experimental image, and the 1A microscope resolution would not account for a situation where a dark spot is missing. It is not clear what analysis was carried out for other cube PNCs such as those shown in Supplementary Figures 17 and 19. Since the structure of the cube is a central finding of the manuscript, the argument would be more convincing if several PNCs could be analyzed and confidence levels presented to show that one structure fits all data best; if I understand the text correctly, only one cube structure was fully analyzed and this analysis was only based on a handful of video frames.

Please note that the (iodo)phenylene groups in the cube are “molecular rotors” and rotated during imaging to produce slightly blurred images. It is the case for atom 8. As indicated by a white arrow below, atom 8 moved during the 0.5 second observation time, and its image merged with the image of atom 1 (which may have rotated toward atom 8).

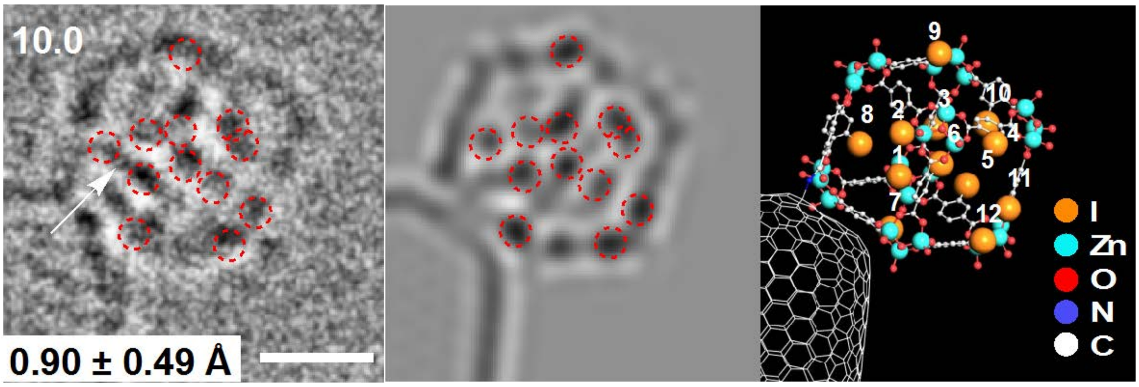

We added the following figure to SI (Supplementary Fig. 5) to show our assignment of the position of iodine atoms of in the TEM and simulated images.

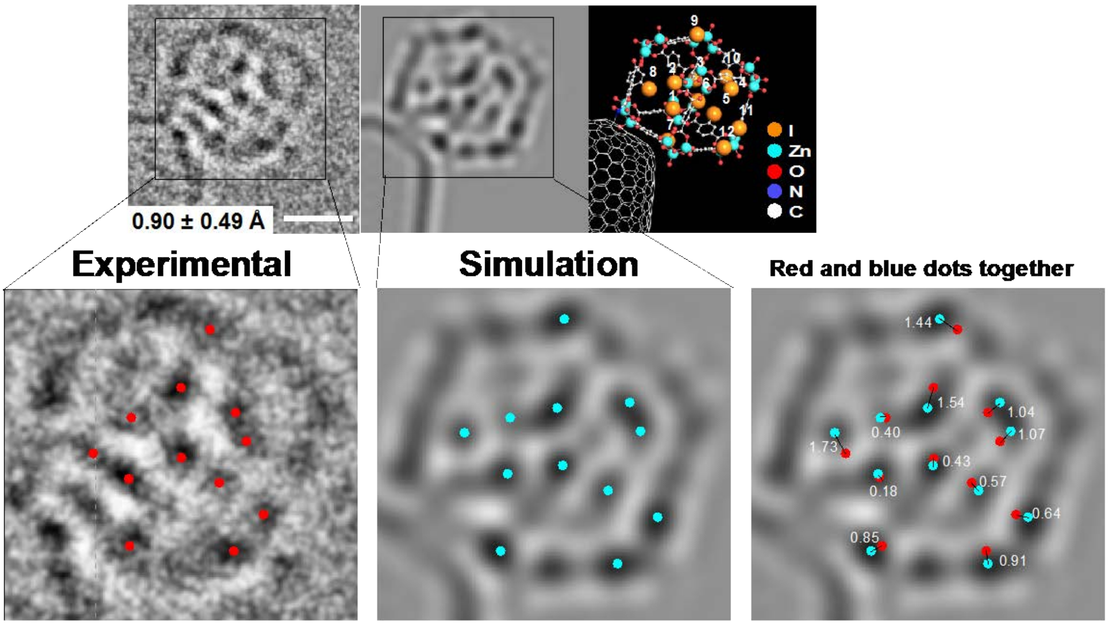

Supplementary Fig. 5. Measurement of average deviation in Å between simulation and TEM image shown in Fig. 4d and 4e. The centers of dark spots in TEM image are shown as red spots, and the centers of dark spots in the simulation image with light blue spots.

(5) The image matching procedure, described throughout the manuscript and the Supplementary Information, appears to be qualitative in relying on a visual estimate of the similarity of simulation and image. Only high-contrast video frames are analyzed. This is based on a reasonable argument about motion, but it is hard to imagine zero motion over 0.4 or 0.5sec in even the high contrast frames. The results would be more convincing if all data were to be included in the analysis with a process that gives less weight to images with less information content, as is common in analysis of cryoelectron microscopy data.

Please note that the PNC molecules underwent bond rotation during observation, and all images we see represent different structural isomers, and hence they are different. Therefore we cannot mix it together with other images.

As the reviewer must be aware of, the ability of SMART to study structural change is a big asset over cryoEM that can analyzed only static molecules frozen in ice.

(A very minor point: In fig 1d and Supplementary Figure 12 I was not sure what the large ovals show.)

These large ovals indicated CNH. We modified this cartoon.

Reviewers' comments:

Reviewer #1 (Remarks to the Author):

The authors have addressed all my concerns regarding the chemistry and statistics in a meaningful manner. By repeating the SMART-EM experiments across the entire reaction time (0-21 hours) and monitoring the pH the manuscript has drastically improved. I find that the finding are very exciting, and recommend to publish this article.
I have only a few minor comments for the authors to address:
- they state "With 27–133 samples examined in each analysis, we expect 8–19% statistical error for the reported HO/LO ratio " Could the authors explain how they calculated this statistical error?
- In my original review I wrote:
"Another question that remains is how representative are these identified PNC's of the the PNC's present in the solution. I mean the fished out PNC's are washed with a solvent and dried in vacuum, and then measured in the TEM in ultrahigh vacuum. Did the clusters not undergo changes in those processes? What are the arguments to state that they are the same clusters as those in the synthesis solution at elevated temperature?"

In their response letter the authors wrote:
"What we want to report are the following: Our first finding is on the cluster size to be similar to that in solution, the second is that the PNCs fished out from the MOF-2 synthesis are different from those from the MOF-5 synthesis, and the third is that the square and the cubic structures of the isolated PNCs are closely related to those in the MOF-2 and -5 crystal lattice.
Some details are given. First, our terephthalic acid "fishhook" is a part structure of MOF crystals, and hence should be able to effectively capture PNCs in solution. Second, we expect that the chemical affinity of our terephthalic acid "fishhook" to PNCs should be roughly equal for various PNCs in MOF-2 and -5 syntheses, and hence that the ratios of clusters reflect those in solution reasonably well. Third, the similarity in size (1 nm) between the DLS data and the clusters captured from solution suggests that we captured right kind of species. Fourth, we believe that the salient features of the EM-observed structures reflect those in solution, because the captured clusters were removed from the reaction mixture by quick filtration and drying in vacuo (dry transfer). Specimens obtained by dry transfer and by MeOH wash shared the same characteristics, and hence we consider that the information obtained by MeOH-wash samples also reflects the structural features of PNCs in the reaction.
That being said, the structures that we saw on EM cannot be exactly the same as those in solution including ligands on metals and conformation of the BDC bridges. However, we consider that the observed square and the cube clusters are reasonable as PNCs to MOF-2 and -5, respectively."

I agree with the reasoning of the authors, however, this point (that the structures in EM won't be exactly the same as in solution, but that there are good reasons to consider them as good approximations of the structure in solution) is missing in the manuscript itself. This should be added.

Reviewer #3 (Remarks to the Author):

I appreciate the authors' effort in responding to numerous referee comments. However, the amended manuscript still does not answer my specific comments. If the authors could respond to these points more quantitatively and also make sure the points are answered in the manuscript and not just the rebuttal letter, I believe the result would be stronger.

(1) The response is qualitative with statements such as
We believe that we did by far the best among what has been done…
[the fishhook] should be able effectively capture…
we expect that the chemical affinity… should be roughly equal…

I was hoping for a more precise response that would convince non-experts, and in addition, that the authors would make this point clear to readers by changing the manuscript specifically to respond to this.

(1b) In their response the authors state
...we believe that the salient features of the EM-observed structures like linear, square vs. cubic reflect those in solution, because we quickly filtered the captured clusters off from the reaction mixture and
immediately dried in vacuo for EM imaging (dry transfer method).

It is difficult for me to imagine that the speed of filtering and drying is sufficient to avoid a chemical structural change.

(2) As a non-expert this response seems to raise more questions than it answers. If methanol changes the coordination of the Zn atoms, it needs to be explained to what extent the measurements are representative of the original solution.

(3) A more precise response would be helpful. How many seconds between events would it take for an event to be described as “exceptional”?

(4) If the groups are molecular rotors one would think that all their images would be blurred. If the image matching procedure is allowed to include the possibility that any one of the groups has rotated, it is harder to make a convincing assignment of structure.

(5) I agree that the SMART technique has large benefits over cryoEM and that this paper shows interesting results. However the importance of the manuscript means that the authors need to be especially careful to include the effect on their analysis of motion during imaging.

Point-by-point response to Reviewers' comments:

(Our response is highlighted in light blue)

Reviewer #1 (Remarks to the Author):

The authors have addressed all my concerns regarding the chemistry and statistics in a meaningful manner. By repeating the SMART-EM experiments across the entire reaction time (0-21 hours) and monitoring the pH the manuscript has drastically improved. I find that the finding are very exciting, and recommend to publish this article.

I have only a few minor comments for the authors to address:

- they state "With 27–133 samples examined in each analysis, we expect 8–19% statistical error for the reported HO/LO ratio " Could the authors explain how they calculated this statistical error?

The calculation method is shown as below. We put this reference as reference 31. Cochran's formula (Cochran, W. G. *Sampling techniques*; John Wiley & Sons: New York, 1977.):

$$d = \sqrt{\frac{p * (1 - p) * t^2}{n_0}}$$

where

d = confidence interval, expressed as decimal (e.g., .04 = ±4%)

p = the estimated proportion of an attribute that is present in the population, expressed as decimal (0.5 is used here)

t = the selected critical value of desired confidence level (e.g. 1.96 for 95% confidence level)

n_0 = sample size for infinite population

- In my original review I wrote:

"Another question that remains is how representative are these identified PNC's of the

the PNC's present in the solution. I mean the fished out PNC's are washed with a solvent and dried in vacuum, and then measured in the TEM in ultrahigh vacuum. Did the clusters not undergo changes in those processes? What are the arguments to state that they are the same clusters as those in the synthesis solution at elevated temperature?"

In their response letter the authors wrote:
"What we want to report are the following: Our first finding is on the cluster size to be similar to that in solution, the second is that the PNCs fished out from the MOF-2 synthesis are different from those from the MOF-5 synthesis, and the third is that the square and the cubic structures of the isolated PNCs are closely related to those in the MOF-2 and -5 crystal lattice.
Some details are given. First, our terephthalic acid "fishhook" is a part structure of MOF crystals, and hence should be able to effectively capture PNCs in solution. Second, we expect that the chemical affinity of our terephthalic acid "fishhook" to PNCs should be roughly equal for various PNCs in MOF-2 and -5 syntheses, and hence that the ratios of clusters reflect those in solution reasonably well. Third, the similarity in size (1 nm) between the DLS data and the clusters captured from solution suggests that we captured right kind of species. Fourth, we believe that the salient features of the EM-observed structures reflect those in solution, because the captured clusters were removed from the reaction mixture by quick filtration and drying in vacuo (dry transfer). Specimens obtained by dry transfer and by MeOH wash shared the same characteristics, and hence we consider that the information obtained by MeOH-wash samples also reflects the structural features of PNCs in the reaction.
That being said, the structures that we saw on EM cannot be exactly the same as those in solution including ligands on metals and conformation of the BDC bridges. However, we consider that the observed square and the cube clusters are reasonable as PNCs to MOF-2 and -5, respectively."

I agree with the reasoning of the authors, however, this point (that the structures in EM won't be exactly the same as in solution, but that there are good reasons to consider

them as good approximations of the structure in solution) is missing in the manuscript itself. This should be added.

We appreciate referee’s comment. We added the following to the Conclusions.
“The data set provided reasons for us to consider that the covalent skeleton of the clusters captured on the CNH fishhooks represents a good approximation of their structure in solution, while they likely differ in details as to the rotational isomerism of the BDC rotors, and the ligands weakly coordinated on the zinc atoms.”

Reviewer #3 (Remarks to the Author):
Comment on first time
Response on first time
Comment on second time
Response on second time

I appreciate the authors’ effort in responding to numerous referee comments. However, the amended manuscript still does not answer my specific comments. If the authors could respond to these points more quantitatively and also make sure the points are answered in the manuscript and not just the rebuttal letter, I believe the result would be stronger.

Comment: (1) In the experiment, the MOF crystallization procedure takes place in the presence of CNHs that are functionalized with BDH that forms sites for PNCs. The authors mention that dynamic light scattering is definitive in showing only one type of PNC in the solution. But the authors need to justify that the PNCs that they measure with SMART, the ones that attached to the CNHs, are the same structure as the ones in solution measured by light scattering. This is not obvious to me as a non-expert in MOF chemistry, since these PNCs have formed in a different environment (in proximity to the “substrate” CNH, with a tethered BDH molecule).

Response: Our “fishing strategy” somewhat resembles “Census of Marine Life Project”, whose validity relies on the sampling technique. We believe that we did by far the best among what has been done to identify PNCs in the MOF synthesis. First, our terephthalic acid “fishhook” to PNCs is a chemically powerful fishhook, because this acid is a part structure of MOF crystals, and hence should be able effectively capture PNCs in solution. Second, we expect that the chemical affinity of our terephthalic acid “fishhook” to PNCs should be roughly equal for various PNCs in MOF-2 and -5 syntheses, and hence that the ratios of clusters reflect those in solution. Third, the similarity in size (1 nm) between the DLS data and the clusters captured from solution suggests that we captured right kind of species. Finally, the square and cube PNCs that we captured are the type of PNCs that people expect for MOF-2 and MOF-5 crystal growth.

Comment: (1) The response is qualitative with statements such as

We believe that we did by far the best among what has been done…

[the fishhook] should be able effectively capture…

we expect that the chemical affinity… should be roughly equal…

I was hoping for a more precise response that would convince non-experts, and in addition, that the authors would make this point clear to readers by changing the manuscript specifically to respond to this.

Response: Upon reading this and the following questions, I realize that the reviewer requires a chemically precise answer stated in the main text. To answer this first question, we added a response on page 7.

“Being a part structure of the covalent network of MOF crystals, the BDC group on CNH serves as a chemically powerful “fishhook” for capturing PNCs in solution so that we can study their structure one by one by EM.”

Please refer to our answer for your comment (2) as to the covalency of the Zn-O bonds.

Comment: (1b) A related question is whether the PNC changed their structure during the drying process, compared to the structure measured by light scattering that is still in the liquid phase.

Response: That being said, the structures that we saw cannot be exactly the same as those in solution, like dried fish vs. live fish. However, we believe that the salient features of the EM-observed structures like linear, square vs. cubic reflect those in solution, because we quickly filtered the captured clusters off from the reaction mixture and immediately dried in vacuo for EM imaging (dry transfer method).

Comment: (1b) In their response the authors state

…we believe that the salient features of the EM-observed structures like linear, square vs. cubic reflect those in solution, because we quickly filtered the captured clusters off from the reaction mixture and immediately dried in vacuo for EM imaging (dry transfer method).

It is difficult for me to imagine that the speed of filtering and drying is sufficient to avoid a chemical structural change.

Response: In response to this request, we added the following also on page 7.

"To stop the MOF forming reactions taking place at high temperature, we cooled down the reaction mixture quickly to 25 °C, immediately followed by removal by filtration of the solvent and the soluble materials to prevent further reactions."

Please note that the MOF synthesis requires long heating at 95—120 °C of a mixture of reactants (Zn + BDC) and solvent (DMF), because the reaction is slow and does not proceed at 25 °C. Therefore, we stopped the reaction by quick cooling down to 25 °C. Filtration then removes solvent and remaining reactants, and hence completely stops further reactions. This is a common procedure that chemists utilize to isolate reaction intermediates to study the mechanism. Thus, we expect a priori that the clusters on CNH seen by EM have the same stoichiometry and covalent structure as those in the hot solution.

Comment: (2) "..washing of the CNH specimens with methanol suppressed ... motions of the CNHs and the PNCs, and allowed us to reproducibly obtain atomic-resolution TEM images without apparent change in the appearance of PNCs..." The mechanism of this methanol-induced stability needs to be explained better, since the highest quality

data is from the immobilized structures making an assumption that they are the same as in the initial liquid sample.

Response: In the dry transfer samples, PNCs sometimes formed a more stable conformation from a less stable one (cf. Fig. 3e). The methanol wash samples were seen to be conformationally more stable, possibly because methanol changed the coordination environment on the zinc atoms (e.g., ligand exchange from DMF to MeOH).

Comment: (2) As a non-expert this response seems to raise more questions than it answers. If methanol changes the coordination of the Zn atoms, it needs to be explained to what extent the measurements are representative of the original solution.

Response: We inadvertently omitted to refer to the inertness of MOF-5 (i.e., the covalent network made of C-C and Zn-O bonds) to methanol (*JACS*, **2007**, *129*, 14176-14177), and to answer this question, we added a sentence on page 7-8.

"Methanol being an inert solvent for MOF-5, the methanol wash did not wash away the clusters made of a covalent Zn-O network, while it may have replaced the weakly coordinated ligands on zinc (such as DMF and nitrate anions, L in Fig. 1d)."

I am a little afraid that there was a lack of consensus among us on what "coordination" means. Water molecules in $Zn(OAc)_2 \cdot 2H_2O$ are weakly "coordinated" to zinc (e.g., L in Fig. 1d), while acetate anions are covalently bonded to zinc. The Zn-O bond is as stable as C-C bonds and is not cleaved in water: Zn-O = 284 kJ/mol and C-C = 300-350 kJ/mol.

Comment: (3) In previous publications, the authors have discussed the stability under the beam of individual molecules in SMART imaging. Here, they show that changes do occur under the beam, such as the change from lower to higher order PNC in Figure 3. Given that changes can occur, the authors need to justify that some rapid change has not occurred at say the onset of imaging that could affect the interpretation.

Response: The rapid conformational changes such as in Fig. 3e are exceptional (and hence highlighted in Fig. 3), and most of the PNCs did not change their morphology

during observation. We consider that these exceptional cases occurred because of thermal vibration of CNH, rather than the cleavage of C-C bonds.

Comment: (3) A more precise response would be helpful. How many seconds between events would it take for an event to be described as “exceptional”?

Response: To avoid confusion, we rewrote a part of the paragraph:

“Particularly noteworthy was spontaneous conversion to what appeared to be cube-like structures as observed for three cases out of several tens of clusters observed clearly. In one example shown in Fig. 3e, a few large rings identifiable in the 3.0-s image started to move after 25 s, forming a stable 3-D structure after 264.0 s (Supplementary Fig. 28) ... We found two additional examples of such conversion for samples after methanol wash (Supplementary Fig. 29 and 30).”

We always performed EM analysis for about 300 seconds, and we saw only three cases of such conversion from mobile to immobile clusters.

Comment: (4) The iodine experiment is a good strategy because it allows more detailed analysis of the images. However, a superficial comparison of experiment and model in fig 4g and h still shows somewhat poor agreement in the positions of the dark spots. For example atom 8 does not show in the experimental image, and the 1A microscope resolution would not account for a situation where a dark spot is missing. It is not clear what analysis was carried out for other cube PNCs such as those shown in Supplementary Figures 17 and 19. Since the structure of the cube is a central finding of the manuscript, the argument would be more convincing if several PNCs could be analyzed and confidence levels presented to show that one structure fits all data best; if I understand the text correctly, only one cube structure was fully analyzed and this analysis was only based on a handful of video frames.

Response: Please note that the (iodo)phenylene groups in the cube are “molecular rotors” and rotated during imaging to produce slightly blurred images. It is the case for atom 8. As indicated by a white arrow below, atom 8 moved during the 0.5 second observation time, and its image merged with the image of atom 1 (which may have rotated toward atom 8).

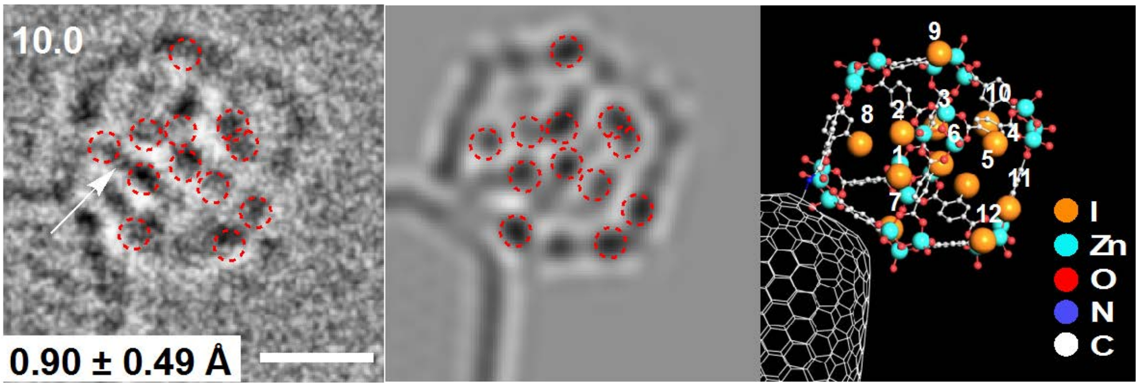

We added the following figure to SI (Supplementary Fig. 5) to show our assignment of the position of iodine atoms of in the TEM and simulated images.

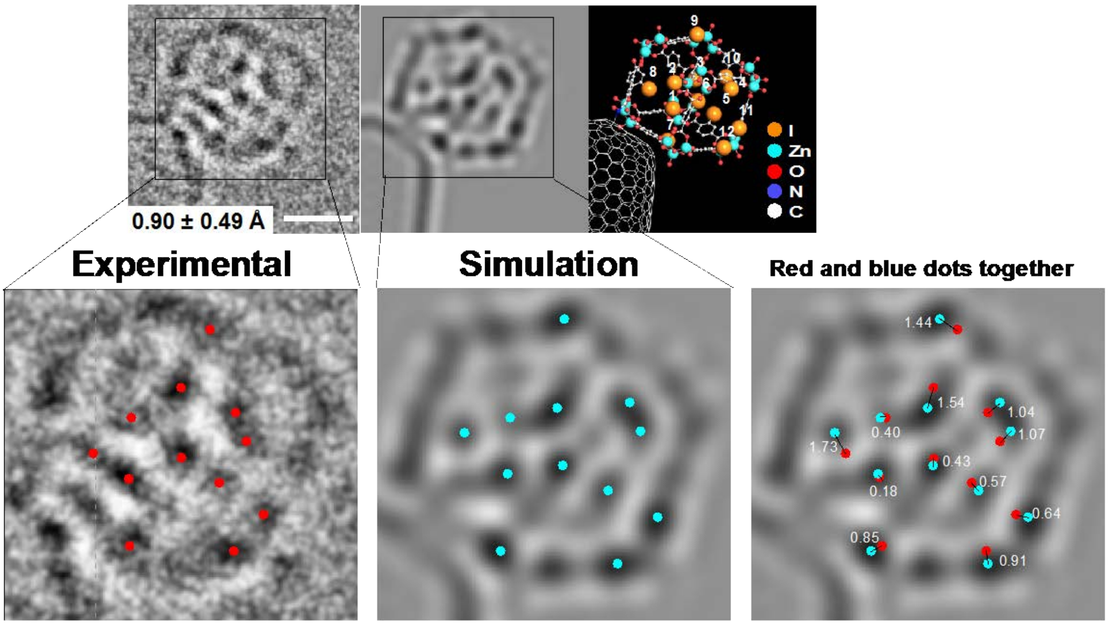

Supplementary Fig. 5. Measurement of average deviation in Å between simulation and TEM image shown in Fig. 4d and 4e. The centers of dark spots in TEM image are shown as red spots, and the centers of dark spots in the simulation image with light blue spots.

Comment: (4) If the groups are molecular rotors one would think that all their images would be blurred. If the image matching procedure is allowed to include the possibility that any one of the groups has rotated, it is harder to make a convincing assignment of structure.

Response: We modified the original statement on P. 14 as below, to avoid misunderstanding that “rotation” means “fast 360° rotation”, which is in reality a

rotational motions by a small angle during the 0.5-s exposure time, as discussed in Supplementary Fig. 5.

“During the rotation of the whole molecule, a few of the IBDC linkers also underwent a small rotational motion during the 0.5-s observation time to cause blurring of some iodine images. (Supplementary Fig. 5).”

Comment: (5) The image matching procedure, described throughout the manuscript and the Supplementary Information, appears to be qualitative in relying on a visual estimate of the similarity of simulation and image. Only high-contrast video frames are analyzed. This is based on a reasonable argument about motion, but it is hard to imagine zero motion over 0.4 or 0.5sec in even the high contrast frames. The results would be more convincing if all data were to be included in the analysis with a process that gives less weight to images with less information content, as is common in analysis of cryoelectron microscopy data.

Response: Please note that the PNC molecules underwent bond rotation during observation, and all images we see represent different structural isomers, and hence they are different. Therefore we cannot mix it together with other images.

As the reviewer must be aware of, the ability of SMART to study structural change is a big asset over cryoEM that can analyzed only static molecules frozen in ice.

Comment: (5) I agree that the SMART technique has large benefits over cryoEM and that this paper shows interesting results. However the importance of the manuscript means that the authors need to be especially careful to include the effect on their analysis of motion during imaging.

Response: We thank the reviewer for this important suggestion. We are hoping that the use of the state-of-the-art camera now available can solve the blurring problem caused by motions.

REVIEWERS' COMMENTS:

Reviewer #3 (Remarks to the Author):

The manuscript is improved since the previous version although unfortunately I still find deficiencies with the authors' response where the authors respond in the rebuttal letter but do not make adequate changes in the manuscript. The important audience is the reader rather than the reviewers.

(1) The authors added:
"Being a part structure of the covalent network of MOF crystals, the BDC group on CNH serves as a chemically powerful "fishhook" for capturing PNCs in solution so that we can study their structure one by one by EM."
Stating that the BDC group is a part structure of the MOF network does not give me, a non-MOF expert, a scientifically precise justification of why the measured PNCs that attached to the CNHs are the same structure as the ones in solution measured by light scattering. Can the authors make an effort to explain this more precisely to readers in the manuscript.

(1b) It is good to have a clear statement that the reaction is slow at 25C so it is not expected to progress once cooled. But it is still not justified that no chemical change will occur on drying. Can the authors cite in the text support for their statement "we expect a priori that the clusters on CNH seen by EM have the same stoichiometry and covalent structure as those in the hot solution."

The authors responded to Referee 1 on a similar point by adding a sentence in the conclusion but it is far less convincing than the long paragraph in the rebuttal letter.

(2, 3) are answered acceptably.

(4, 5) The authors state "a few of the IBDC linkers also underwent a small rotational motion"
Can you explain in the manuscript the justification for knowing the amount of rotation that is possible. Also, in my response to (5) I asked the authors "to be especially careful to include the effect on their analysis of motion during imaging". Readers of the paper will not find this effect is included by the statement in the rebuttal letter. Even the best cameras that achieve say 1000 fps will still not resolve molecular motion. Overall I am still not convinced from the material in the manuscript that the image matching process is sufficiently justified.

Point-by-point response to reviewer's comments

(The reviewer's comments are shown in blue.)

Reviewer #3 (Remarks to the Author):

The manuscript is improved since the previous version although unfortunately I still find deficiencies with the authors' response where the authors respond in the rebuttal letter but do not make adequate changes in the manuscript. The important audience is the reader rather than the reviewers.

Thank you very much for taking time for the careful review on our manuscript. We took the reviewer's concern into account and further revised the manuscript as follows.

(1) The authors added:
"Being a part structure of the covalent network of MOF crystals, the BDC group on CNH serves as a chemically powerful "fishhook" for capturing PNCs in solution so that we can study their structure one by one by EM."
Stating that the BDC group is a part structure of the MOF network does not give me, a non-MOF expert, a scientifically precise justification of why the measured PNCs that attached to the CNHs are the same structure as the ones in solution measured by light scattering. Can the authors make an effort to explain this more precisely to readers in the manuscript.

(1b) It is good to have a clear statement that the reaction is slow at 25C so it is not expected to progress once cooled. But it is still not justified that no chemical change will occur on drying. Can the authors cite in the text support for their statement "we expect a priori that the clusters on CNH seen by EM have the same stoichiometry and covalent structure as those in the hot solution."

The authors responded to Referee 1 on a similar point by adding a sentence in the conclusion but it is far less convincing than the long paragraph in the rebuttal letter.

We added the following sentence in conclusion part to indicate the covalent scaffold made of Zn and BDC is maintained after drying due to the lack of materials transfer.

"namely, we expect a priori that the clusters on BDC-CNH seen by EM have stoichiometry and covalent structure closely related to those in the reaction mixture."

(2, 3) are answered acceptably.

(4, 5) The authors state "a few of the IBDC linkers also underwent a small rotational motion"
Can you explain in the manuscript the justification for knowing the amount of

rotation that is possible. Also, in my response to (5) I asked the authors “to be especially careful to include the effect on their analysis of motion during imaging”. Readers of the paper will not find this effect is included by the statement in the rebuttal letter. Even the best cameras that achieve say 1000 fps will still not resolve molecular motion. Overall I am still not convinced from the material in the manuscript that the image matching process is sufficiently justified.

According to the suggestion, we mentioned the motion blur of PNC in the I-MOF-5 subsection.

“During the rotation of the whole molecule, a few of the IBDC linkers also underwent a small rotational motion during the 0.5-s observation time to cause blurring of some iodine images, allowing us to assign a cube structure having 12 iodine atoms to the cluster, and the regiochemistry of the iodine atoms on the BDC linker, but not the structural details of the rotational isomerism.”
